# Numerous new records of tropical non-indigenous species in the Eastern Mediterranean highlight the challenges of their recognition and identification

**DOI:** 10.3897/zookeys.1010.58759

**Published:** 2021-01-13

**Authors:** Paolo G. Albano, Jan Steger, Piet A.J. Bakker, Cesare Bogi, Marija Bošnjak, Tamar Guy-Haim, Mehmet Fatih Huseyinoglu, Patrick I. LaFollette, Hadas Lubinevsky, Martina Mulas, Martina Stockinger, Michele Azzarone, Bruno Sabelli

**Affiliations:** 1 Department of Palaeontology, University of Vienna, Althanstrasse 14, 1090, Vienna, Austria University of Vienna Vienna Austria; 2 Naturalis Biodiversity Center, Darwinweg 2, 2333, CR Leiden, The Netherlands Naturalis Biodiversity Center Leiden Netherlands; 3 Gruppo Malacologico Livornese, c/o Museo di Storia Naturale del Mediterraneo, via Roma 234, 57127, Livorno, Italy Museo di Storia Naturale del Mediterraneo Livorno Italy; 4 Croatian Natural History Museum, Demetrova 1, Zagreb, Croatia Croatian Natural History Museum Zagreb Croatia; 5 National Institute of Oceanography, Israel Oceanographic and Limnological Research (IOLR), Haifa 3108001, Israel Israel Oceanographic and Limnological Research Haifa Israel; 6 Faculty of Maritime Studies, University of Kyrenia, Karakum, Girne, Turkish Republic of Northern Cyprus University of Kyrenia Girne Cyprus; 7 Malacology Section, Natural History Museum of Los Angeles County, 900 Exposition Blvd., Los Angeles, CA 90007, USA Natural History Museum of Los Angeles County Los Angeles United States of America; 8 The Leon H. Charney School of Marine Sciences, University of Haifa, 199 Aba Khoushy Ave., Mt. Carmel, Haifa 3498838, Israel University of Haifa Haifa Israel; 9 Museo di Zoologia dell’Università di Bologna, via Selmi 3, 40126, Bologna, Italy Università di Bologna Bologna Italy

**Keywords:** Cerithiopsidae, invasion biology, Lessepsian invasion, Mollusca, new species, Red Sea, taxonomy, Triphoridae

## Abstract

New data on 52 non-indigenous mollusks in the Eastern Mediterranean Sea is reported. *Fossarus* sp. (aff. aptus sensu Blatterer 2019), *Coriophora lessepsiana* Albano, Bakker & Sabelli, **sp. nov.**, Cerithiopsis sp. aff. pulvis, *Joculator problematicus* Albano & Steger, **sp. nov.**, *Cerithiopsis* sp., *Elachisina* sp., Iravadia aff. elongata, *Vitrinella* aff. *Vitrinella* sp. 1 (sensu Blatterer 2019), *Melanella orientalis*, Parvioris aff. dilecta, Odostomia cf. dalli, *Oscilla virginiae*, *Parthenina cossmanni*, *Parthenina typica*, *Pyrgulina craticulata*, *Turbonilla funiculata*, *Cylichna collyra*, *Musculus coenobitus*, Musculus aff. viridulus, *Chavania erythraea*, Scintilla cf. violescens, *Iacra seychellarum* and *Corbula erythraeensis* are new records for the Mediterranean. An unidentified gastropod, Skeneidae indet., *Triphora* sp., *Hypermastus* sp., *Sticteulima* sp., Vitreolina cf. philippi, *Odostomia* (s.l.) sp. 1, *Henrya* (?) sp., and Semelidae sp. are further potential new non-indigenous species although their status should be confirmed upon final taxonomic assessment. Additionally, the status of *Dikoleps micalii*, *Hemiliostraca clandestina***comb. nov.** and *H. athenamariae***comb. nov.** is changed to non-indigenous, range extensions for nine species and the occurrence of living individuals for species previously recorded from empty shells only are reported. *Opimaphora blattereri* Albano, Bakker & Sabelli, **sp. nov.** is described from the Red Sea for comparison with the morphologically similar *C. lessepsiana* Albano, Bakker & Sabelli, **sp. nov.** The taxonomic part is followed by a discussion on how intensive fieldwork and cooperation among institutions and individuals enabled such a massive report, and how the poor taxonomic knowledge of the Indo-Pacific fauna hampers non-indigenous species detection and identification. Finally, the hypothesis that the simultaneous analysis of quantitative benthic death assemblages can support the assignment of non-indigenous status to taxonomically undetermined species is discussed.

## Introduction

The Eastern Mediterranean Sea is a hotspot of non-indigenous species introductions. The opening of the Suez Canal in 1869 broke a long-standing biogeographic barrier and enabled hundreds of Red Sea species to enter the basin and establish populations (Por 1978; Galil 2009; Zenetos et al. 2010, 2017; Zenetos and Galanidi 2020). These so-called Lessepsian species are now recorded from all countries bordering this basin west to Greece (Katsanevakis et al. 2009; Çinar et al. 2011; Ammar 2018; Zenetos et al. 2018; Bariche and Fricke 2020; Crocetta et al. 2020) and some have already reached the central Mediterranean, e.g., Tunisia (Ounifi-Ben Amor et al. 2015), Italy (Occhipinti-Ambrogi et al. 2011), and even France (Daniel et al. 2009; Bodilis et al. 2011).

The introduction rate is an important metric to describe the invasion process. Genuine variation in this rate can result from changes in vector efficacy, connectivity between the native and introduced range, and environmental conditions in the recipient ecosystem. The introduction rate is often estimated from the discovery record (Solow and Costello 2004). However, even in well sampled and taxonomically well-known groups like mollusks, multi-decadal time lags between introduction and first detection have been quantified (Oliver 2015; Guy-Haim et al. 2017; Albano et al. 2018), suggesting that the detection rate is a poor proxy of the introduction rate. Indeed, although the discovery rate is increasing (Galil 2009; Raitsos et al. 2010), estimates of the introduction rate corrected for temporal variation in sampling effort for Lessepsian fishes showed that it was constant over ~ 1930–2010 (Belmaker et al. 2009). Still, the most recent enlargement of the Suez Canal has raised concerns that the improved connectivity could increase the introduction rate of Lessepsian species (Galil et al. 2015). Additionally, rapid climate warming is particularly affecting the Eastern Mediterranean (Ozer et al. 2017), causing, on the one hand, the decline of native species and, on the other hand, more favourable conditions for the establishment of tropical species (Rilov 2016; Albano et al. 2021).

To monitor a dynamic process such as the Lessepsian invasion, intensive fieldwork is mandatory. We indeed show that an intensive sampling effort coupled with identification at high taxonomic resolution and collaborative research among individuals and institutions enabled the detection of 23 new Lessepsian mollusks, another nine species which, upon further inspection, may prove to be new Lessepsian species, nine new records for Eastern Mediterranean countries, and new data for eleven already recognized non-indigenous species. We here describe these new findings, providing detailed collecting data, taxonomic comments, and comparisons with similar species.

## Materials and methods

### Origin of samples

The studied material comes from three main sources. First, sampling on the Israeli Mediterranean shelf performed in the context of the project “Historical ecology of Lessepsian migration” (**HELM**), in progress at the University of Vienna. Second, benthic assemblage monitoring by the Israel Oceanographic and Limnological Research (**IOLR**). Third, smaller scale sampling by some of us, further detailed in the Results section.

Sampling in the framework of the HELM project was conducted on soft substrates between 10 and 40 m depth with a van Veen grab, and on hard substrates between 5 and 30 m by diver-operated airlift suction sampling, using 0.5 mm mesh-size net bags. Samples were sieved with a 0.5 mm mesh and the retained material fixed in 95% ethanol. Both living individuals and empty shells were identified and counted.

IOLR conducts regular monitoring of Israeli soft bottom benthic assemblages in the framework of the National Monitoring (NM) and focused sampling for environmental assessment (APM DAN, Shafdan, Via Maris). The NM, APM DAN and Via Maris projects sampled soft substrates with a 0.11 m^2^ van Veen grab at depths between 6 and 12.5 m (NM), 22 and 26.5 m (APM DAN) and 18 and 26 m (Via Maris). Samples were sieved with a 250 μm mesh. During the Shafdan project, three replicate sediment samples were taken at each station from a different 0.062 m² box-corer launch (Ocean Instruments model 700 AL) twice a year in spring (May) and fall (October). The samples were sieved on board with a 0.5 mm mesh. All samples were preserved in 99% ethanol, stained with eosin solution (hence the pink hue that some specimens bear) and picked for living individuals.

Finally, we included serendipitous findings by some of us or by colleagues within our extended network, from multiple localities. For each species, we provide detailed collecting data following the guidelines by Chester et al. (2019).

### Taxonomic assignment and non-indigenous status attribution

The depth of taxonomic assignment varies across taxa, mostly reflecting the available knowledge on these groups in the Indo-Pacific province (the source pool of most non-indigenous species in the Eastern Mediterranean). For families like the Triphoridae, some of us (PGA, PAJB, and BS) have been conducting taxonomic research for a long time and we have thus been able to describe new species as we have robust knowledge of inter- and intraspecific variability and of type specimens (Albano et al. 2011, 2017, 2019; Albano and Bakker 2016). For other families, like the Eulimidae, we focused our attention on highlighting differences from native species and similarities with Indo-Pacific species, because a more thorough coverage would have required revising the taxonomy of entire Indo-Pacific species-groups, a task well beyond our objectives. In all cases, we strove to provide detailed and high-quality images as a basis to foster further research and enable the scientific community to refine our identifications. The use of qualifiers for species left in open nomenclature follows the recommendations of Sigovini et al. (2016).

Acknowledging that an unsettled taxonomic status implies uncertainty in the assignment of non-indigenous status (Marchini et al. 2015), we here tagged as non-indigenous only the species which: i) unequivocally belong to Indo-Pacific species; ii) belong to clades (genera or families) that do not occur in the Mediterranean Sea, even if left in open nomenclature; iii) belong to species whose diagnostic characters did not enable a clear attribution to a non-Mediterranean clade but that were found alive while not, or only very rarely, in the death assemblage (see Discussion). In contrast, we tagged as “potential” non-indigenous species those whose morphological characters did not allow for an unambiguous attribution to a non-Mediterranean clade and that were found mostly, or exclusively, as empty shells.

### Imaging and reporting

Small specimens were photographed with a Zeiss SteREO Discovery.V20 stereomicroscope, larger ones with a Nikon D7200 camera mounted on a stand, using a Nikon Micro-Nikkor 60 mm lens. Photographs were stacked with Helicon Focus 6. Scanning electron microscope (SEM) images were shot with a Fei Inspect S50 at low-vacuum mode without coating. The internal shell morphology of *Odostomia* (s.l.) sp. 1, with a particular focus on the intorted protoconch, was visualized using a Phoenix v|tome|x s research edition computer tomographic (CT) scanner. The 3D-reconstruction and virtual sections through the shell were produced with VGSTUDIO MAX 2.1 software. The X-ray image stack, mesh files, virtual sections, and a video showing the interior of the shell are available from the Figshare repository (https://doi.org/10.6084/m9.figshare.c.5215226). Plates were mounted with the image manipulation software GIMP 2.

For each new non-indigenous species record, we report the size of at least one specimen (usually the one figured, unless otherwise stated). The systematic arrangement follows Bouchet et al. (2010, 2017). Table 1 summarizes the species treated in this work.

**Table 1.** List of the taxa treated in this paper, with indication of the novelty of the records.

Family	Taxon	Novelty	Page	Figure
–	Unidentified gastropod	Potential new NIS for the Mediterranean Sea	6	Figure 1
Conradiidae	*Conradia eutornisca*	First record of living individuals in the Mediterranean Sea	7	–
Skeneidae	*Dikoleps micalii*	Declared NIS in the Mediterranean Sea. First record from Israel; first record of living individuals in the Mediterranean Sea	8	Figure 2
	Skeneidae indet.	Potential new NIS for the Mediterranean Sea	9	Figure 3
Planaxidae	*Fossarus* sp. (aff. aptus sensu Blatterer, 2019)	New NIS for the Mediterranean Sea	10	Figure 4
Epitoniidae	*Cycloscala hyalina*	First record of living individuals in the Mediterranean Sea	11	–
Naticidae	*Eunaticina papilla*	First record of living individuals in Israel	12	Figure 5
Triphoridae	*Coriophora lessepsiana* Albano, Bakker & Sabelli, sp. nov.	New NIS for the Mediterranean Sea	12	Figure 6
	*Opimaphora blattereri* Albano, Bakker & Sabelli, sp. nov.	New species from the Red Sea, for comparison with the non-indigenous *Coriophora lessepsiana* Albano, Bakker & Sabelli, sp. nov.	15	Figure 7
	*Triphora* sp.	Potential new NIS for the Mediterranean Sea	18	Figure 8
	Viriola cf. bayani	New reports of living individuals from Israel	19	–
Cerithiopsidae	Cerithiopsis sp. aff. pulvis	New NIS for the Mediterranean Sea	20	Figure 9
	*Cerithiopsis* sp.	New NIS for the Mediterranean Sea	20	Figure 10
	*Joculator problematicus* Albano & Steger, sp. nov.	New NIS for the Mediterranean Sea	23	Figure 11
Elachisinidae	*Elachisina* sp.	New NIS for the Mediterranean Sea	26	Figure 12
Iravadiidae	Iravadia aff. elongata	New NIS for the Mediterranean Sea	26	Figure 13
Vitrinellidae	*Vitrinella* aff. *Vitrinella* sp. 1 (sensu Blatterer 2019)	New NIS for the Mediterranean Sea	29	Figure 14
Eulimidae	*Hypermastus* sp.	Potential new NIS for the Mediterranean Sea	31	Figure 15
	*Hemiliostraca clandestina* comb. nov.	Declared NIS in the Mediterranean Sea. First record from Israel; first record of living individuals in the Mediterranean Sea	31	Figure 16
	*Melanella orientalis*	New NIS for the Mediterranean.	33	Figure 17
	Parvioris aff. dilecta	New NIS for the Mediterranean Sea	35	Figure 18
	*Sticteulima* sp.	Potential new NIS for the Mediterranean Sea	37	Figure 19
	Vitreolina cf. philippi	Potential new NIS for the Mediterranean Sea	37	Figure 16
Conidae	*Conus fumigatus*	First record from Israel	38	Figure 20
Murchisonellidae	*Henrya* (?) sp.	Potental new NIS for the Mediterranean Sea	39	Figure 21
Pyramidellidae	Odostomia cf. dalli	New NIS for the Mediterranean Sea	41	Figure 22
	*Odostomia* (s.l.) sp. 1	Potential new NIS for the Mediterranean Sea	41	Figure 23
	*Odostomia* (s.l.) sp. 2	First record of a living individual in the Mediterranean Sea	44	Figure 24
	*Oscilla virginiae*	New NIS for the Mediterranean Sea	45	Figure 25
	*Parthenina cossmanni*	New NIS for the Mediterranean Sea	47	Figure 26
	*Parthenina typica*	New NIS for the Mediterranean Sea	49	Figure 27
	*Pyrgulina craticulata*	New NIS for the Mediterranean Sea	49	Figure 28
	*Pyrgulina nana*	First record of living individuals in Israel	53	–
	Turbonilla funiculata	New NIS for the Mediterranean Sea	53	Figure 29
Cylichnidae	*Cylichna collyra*	New NIS for the Mediterranean Sea	55	Figure 30
Mnestiidae	*Mnestia girardi*	First record of living individuals in the Mediterranean Sea	57	–
Haminoeidae	*Atys angustatus*	First record from Greece	57	Figure 31
Mytilidae	*Arcuatula perfragilis*	Additional records of living individuals from Israel	58	Figure 32
	*Lioberus ligneus*	First record from Cyprus and Israel	58	Figure 33
	*Musculus coenobitus*	New NIS for the Mediterranean Sea	60	Figure 34, Figure 35
	Musculus aff. viridulus	New NIS for the Mediterranean Sea	61	Figure 36
Isognomonidae	Isognomon aff. australica (sensu Angelidis and Polyzoulis 2018)	New record from Cyprus	65	Figure 37
Lucinidae	Pegophysema cf. philippiana	First record of living individuals in the Mediterranean Sea	66	Figure 38
	*Chavania erythraea*	New NIS for the Mediterranean Sea	67	Figure 39
	*Rugalucina angela*	Additional records of living individuals from Israel	67	–
Galeommatidae	Nudiscintilla cf. glabra (sensu Mifsud and Ovalis 2012)	First record from Israel	68	Figure 40
Galeommatidae	Scintilla cf. violescens	New NIS for the Mediterranean Sea	69	Figure 41
Psammobiidae	*Gari pallida*	Additional records of living individuals from Israel	72	–
Semelidae	*Ervilia scaliola*	First record from Israel	72	Figure 42
	*Iacra seychellarum*	New NIS for the Mediterranean Sea	72	Figure 43
	Semelidae sp.	Potential new NIS for the Mediterranean Sea	74	Figure 44
Veneridae	*Clementia papyracea*	Additional records of living individuals from Israel	76	–
Corbulidae	*Corbula erythraeensis*	New NIS for the Mediterranean Sea	76	Figure 45

### Abbreviations

**BPBM**Bernice Pauahi Bishop Museum, Honolulu, Hawaii, USA;

**H** height;

**HELM** “Historical ecology of Lessepsian migration” project;

**IOLR** Israel Oceanographic and Limnological Research;

**L** length;

**LACM**Natural History Museum of Los Angeles County, Los Angeles, California, United States;

**MNHN**Museum national d’Histoire naturelle, Paris, France;

**MSNG**Museo Civico di Storia Naturale di Genova “Giacomo Doria”, Genova, Italy;

**MZUB**Museum of Zoology of the University of Bologna, Italy;

**NHMW** Natural History Museum, Vienna, Austria;

**NM** National Monitoring Israel;

**OLML**Oberösterreichisches Landesmuseum Linz, Austria;

**RMNH** Rijksmuseum van Natuurlijke Historie (now Naturalis Biodiversity Center), Leiden, The Netherlands;

**SMF**Senckenberg Museum Frankfurt, Germany;

**SMNH** Steinhardt Museum of Natural History, Tel Aviv, Israel;

**sh**/**shs** empty shell/s;

**spcm**/**spcms** live collected specimen/s;

**v**/**vv** valve/s;

**W** width.

## Results

### Class Gastropoda Cuvier, 1795

#### Family unassigned (Caenogastropoda)

**Unidentified gastropod**

Figure 1

**New records.**

Israel • 1 spcm; Haifa Bay; 32.8211°N, 35.0196°E; depth 11 m; 2 Aug. 2015; soft substrate; grab; NM project (sample HM27(c)); size: H 2.5 mm, W 1.6 mm.

**Remarks.**

We were not able to confidently assign this specimen to any family. The general characters suggest that it is a caenogastropod. This specimen has apparently traces of the animal inside and has thus been considered live collected. However, as it was found in Haifa Bay, we cannot exclude that it comes from freshwater or transitional ecosystems (the adjacent Kishon River and estuary) whose waters flow into the bay. An anatomical study of the soft parts, should another living specimen become available, will clarify the taxonomic placement of this intriguing species.

**Figure 1.**
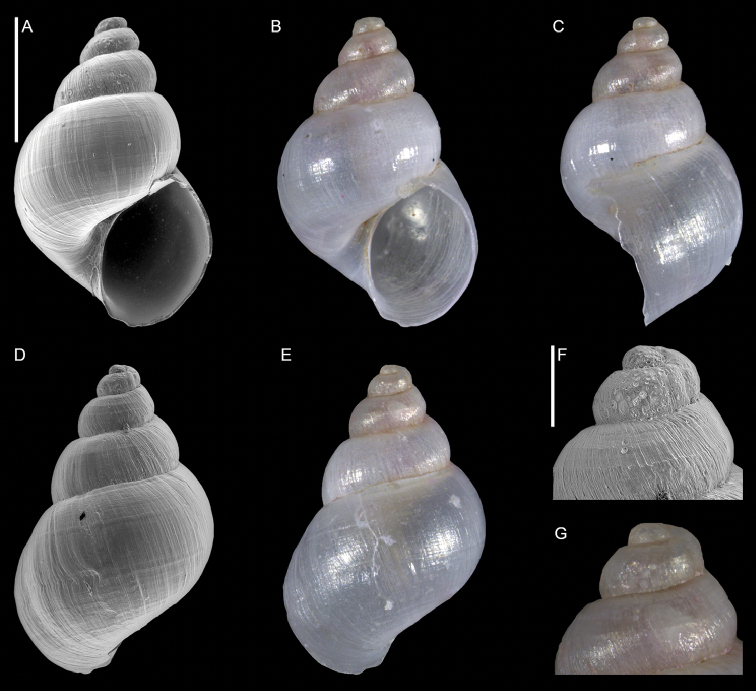
Unidentified gastropod (Caenogastropoda), Haifa Bay, Israel (sample HM27(c)): front (**A, B**), side (**C**) and back (**D, E**) views, protoconch (**F, G**). Scale bars: 1 mm (**A–E**); 0.3 mm (**F–G**).

#### Family Conradiidae Golikov & Starobogatov, 1987

##### Conradia eutornisca

Taxon classificationAnimaliaTrochidaConradiidae

(Melvill, 1918)

99A36CD8-D038-569F-8DB6-4226540DF248

###### New records.

Israel • 1 sh; Ashqelon; 31.7101°N, 34.5406°E; depth 31 m; 18 Sep. 2016; muddy-sand; grab; HELM project (sample SG30_5F) • 3 spcms; Ashqelon; 31.6868°N, 34.5516°E; depth 11 m; 31 Oct. 2018; offshore rocky reef; suction sampler; HELM project (samples S58_1M, S58_2F) • 3 spcms; Ashqelon; 31.6891°N, 34.5257°E; depth 25 m; 2 May 2018; offshore rocky reef; suction sampler; HELM project (sample S16_1M) • 1 spcm; same collecting data as for preceding; depth 28 m; 31 Oct. 2018; HELM project (sample S59_3F) • 1 spcm, 1 sh; west of Rosh HaNikra Islands; 33.0704°N, 35.0926°E; depth 12 m; 1 May 2018; rocky substrate; suction sampler; HELM project (samples S14_1F, S14_3F) • 3 spcms; same collecting data as for preceding; 29 Oct. 2018; HELM project (samples S52_1M, S52_3F, S52_3M) • 12 spcms, 4 shs; west of Rosh HaNikra Islands; 33.0725°N, 35.0923°E; depth 20 m; 1 May 2018; rocky substrate; suction sampler; HELM project (samples S13_1F, S13_1M, S13_2F, S13_3F, S13_3M) • 14 spcms; same collecting data as for preceding; depth 19 m; 29 Oct. 2018; HELM project (samples S53_1F, S53_1M, S53_2F, S53_2M, S53_3F, S53_3M); size: H 2 mm, W 1.7 mm.

###### Remarks.

The species has already been reported from Israel and Turkey (Bogi and Galil 1999; Buzzurro and Cecalupo 2006), but only from empty shells. To the best of our knowledge, this is the first record of living individuals from the Mediterranean Sea. Based on our observations, it occurs rather frequently on shallow subtidal rocky substrates. The samples from Turkey led to the description of *Parviturbo dibellai* Buzzurro & Cecalupo, 2006, at that time supposed to be a native species, but this name was later recognized to be a synonym of the Indo-Pacific *Fossarus eutorniscus* (Rubio et al. 2015), attributed to *Conradia* by Janssen et al. (2011). Janssen et al. (2011) highlighted, however, that the Red Sea specimens have seven spiral cords instead of the five cited in the original description based on material from Karachi (Pakistan). Specimens with five spiral cords occur also in the Persian (Arabian) Gulf and rarely in the Red Sea (H. Dekker, pers. comm., November 2020). Further research is required to ascertain if these two morphologies belong to two different taxa.

#### Family Skeneidae W. Clark, 1851

##### Dikoleps micalii

Taxon classificationAnimaliaTrochidaSkeneidae

Agamennone, Sbrana, Nardi, Siragusa & Germanà, 2020

47569C9B-8B2F-5896-880A-49898AA61774

Figure 2

###### New records.

Israel • 4 spcms; Haifa Bay; 32.8211°N, 35.0196°E; depth 11 m; 2 Aug. 2015; soft substrate; grab; NM project (samples HM27(a) and HM27(c)); size of largest specimen: H 0.7 mm, W 0.7 mm.

###### Remarks.

This species has been recently described from sediment collected in 2016 at 33–45 m depth at Karpathos and Samos islands in the eastern Aegean Sea (Agamennone et al. 2020b). The authors discussed but declined the possibility that this is a Lessepsian species, but one of us (BS) observed non-distinguishable specimens from the Red Sea and we received reports of further indistinguishable specimens from the Persian (Arabian) Gulf (H. Dekker, pers. comm., November 2020). This is the first record for Israel; all specimens were live collected.

**Figure 2.**
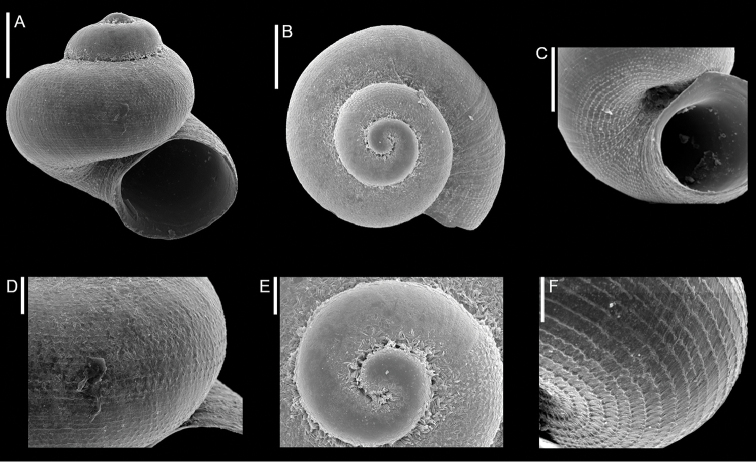
*Dikoleps micalii* Agamennone, Sbrana, Nardi, Siragusa & Germanà, 2020, Haifa Bay, Israel: front (**A**) and apical (**B**) views, umbilicus (**C**), microsculpture of body whorl (**D**), apical view of protoconch (**E**) and microsculpture of the base (**F**). Photograph courtesy A. Bonfitto. Scale bars: 0.2 mm (**A–C**); 0.05 mm (**D–F**).

##### Skeneidae

Taxon classificationAnimaliaTrochidaSkeneidae

indet.

F39C65A0-5FF0-5091-A565-9ED08620A347

Figure 3

###### New records.

Israel • 1 sh; Akko; 32.92°N, 35.07°E; depth 4 m; 22 Oct. 1998; shell grit sample; size: H 0.6 mm, W 1.0 mm.

###### Remarks.

This tiny gastropod (largest diameter 1 mm) is characterized by a small but solid shell, ~ 0.75 whorls of protoconch with axial costae visible near the proto-teleoconch transition (more costae closer to the nucleus may be abraded), and two teleoconch whorls with numerous regular spiral cords. The shoulder is slightly angulated near the lip. Umbilicus open, large. Shell white, slightly translucent. No native Mediterranean species shares these features. Only *Skenea catenoides* (Monterosato, 1877) has a similarly solid shell with numerous regular spiral cords, but it can be distinguished easily by the three nodulose thicker spiral cords on the base and the lack of angulation at the shoulder. Both Mediterranean (e.g., *Circulus striatus* (Philippi, 1836) and Red Sea *Circulus* (e.g., *C. novemcarinatus* (Melvill, 1906a)) and *C. octoliratus* (Carpenter, 1856)) can be distinguished by the multispiral protoconch and the much more prominent spiral cords. It is most likely a new, probably still unnamed, Indo-Pacific species in the Mediterranean.

**Figure 3.**
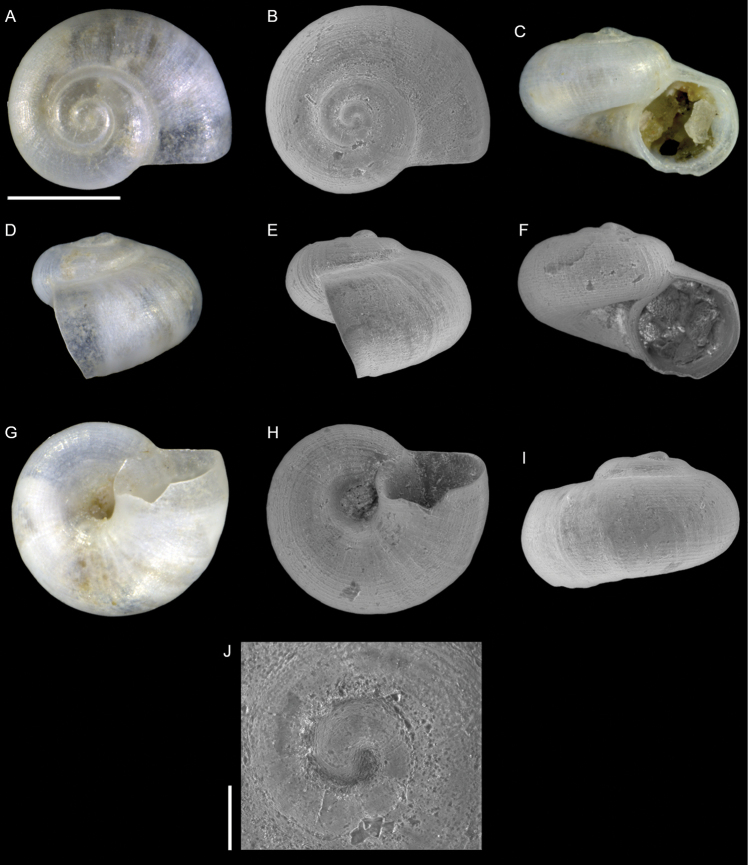
Skeneidae indet., Akko, Israel: apical (**A, B**), front (**C, F**), side (**D, E**), umbilical (**G, H**) and back (**I**) views, protoconch (**J**). Scale bars: 0.5 mm (**A–I**); 0.1 mm (**J**).

#### Family Planaxidae Gray, 1850

##### Fossarus

Taxon classificationAnimaliaPlanaxidae

sp. (aff. aptus sensu Blatterer 2019)

474FEED8-3725-513D-A641-24EC79585E6A

Figure 4

###### New records.

Israel • 1 sh; Ashqelon; 31.6868°N, 34.5516°E; depth 11 m; 31 Oct. 2018; offshore rocky reef; suction sampler; HELM project (sample S58_3M); size: H 3.3 mm, W 2.7 mm.

###### Remarks.

We found a single empty shell of this *Fossarus* that can be readily distinguished from the Mediterranean *F. ambiguus* (Linnaeus, 1758), which bears prominent spiral ridges and has a depressed spire. In contrast, our shell bears numerous spiral cords and has a high spire. This shell is extremely similar to “Fossarus aff. aptus Melvill, 1912” illustrated by Blatterer (2019: plate 87, fig. 7a–f). Especially the largest specimen (plate 87, fig. 7a, b) bears a sculpture of similarly depressed and closely arranged spiral cords, has a very similar profile and a large elongated umbilical area. Blatterer’s specimen shows, however, more regularly alternated thicker and finer cords, whereas in our specimen this feature is not so evident. Our specimen is almost the double in size and rather worn, which may explain the observed differences in sculpture. The extreme similarity with Blatterer’s Red Sea specimens suggests that this is a new non-indigenous species in the Mediterranean Sea.

The name *aptus* is problematic. Originally introduced by Melvill (1912) for a species from the Persian (Arabian) Gulf, it is currently considered a synonym of the Atlanto-Mediterranean *F. ambiguus* (MolluscaBase 2020), but no revision of this genus is available. However, this name fits neither Blatterer’s specimens nor ours because *F. aptus* is characterized by five strong spiral keels (indeed similar to *F. ambiguus*).

**Figure 4.**
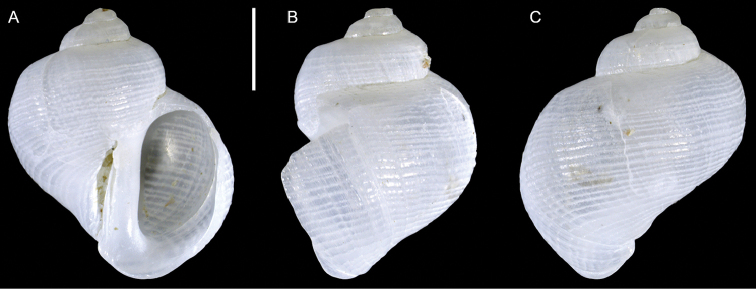
*Fossarus* sp. (aff. aptus sensu Blatterer, 2019), Ashqelon, Israel, HELM project (sample S58_3M): front (**A**), side (**B**) and back (**C**) views. Scale bar: 1 mm.

#### Family Epitoniidae Berry, 1910 (1812)

##### Cycloscala hyalina

Taxon classificationAnimaliaEpitoniidae

(G.B. Sowerby II, 1844)

6F09BA9B-8FAD-53EC-99CB-E266B41589B6

###### New records.

Israel • 2 spcms; west of Rosh HaNikra Islands; 33.0704°N, 35.0926°E; depth 12 m; 29 Oct. 2018; rocky substrate; suction sampler; HELM project (samples S52_1M, S52_2F); size of largest specimen: H 3.7 mm, W 2.0 mm.

###### Remarks.

The species has been recently recorded for the Mediterranean Israeli coastline based on empty shells collected off the Soreq desalination plant, ~ 15 km south of Tel Aviv (Scaperrotta et al. 2019). The species is also known from Cyprus (Cecalupo and Quadri 1994), Turkey (Giunchi et al. 2001), and Greece (Scaperrotta et al. 2019). To our knowledge, this is the first record of living individuals in the Mediterranean Sea.

#### Family Naticidae Guilding, 1834

##### Eunaticina papilla

Taxon classificationAnimaliaLittorinimorphaNaticidae

(Gmelin, 1791)

A323F133-087A-55E9-B9FE-B3904B4FE350

Figure 5

###### New records.

Israel • 1 spcm; Ashdod; 31.8758°N, 34.6465°E; depth 27 m; 17 May 2017; soft substrate; grab; APM DAN project (sample 8C); size: H 1.1 mm, W 1.4 mm (illustrated specimen) • 1 sh; rocky reef off Sdot Yam; 32.5111°N, 34.8702°E; depth 28 m; 1 Nov. 2018; hard substrate; suction sampler; HELM project (sample S60_2M).

###### Remarks.

**Figure 5.**
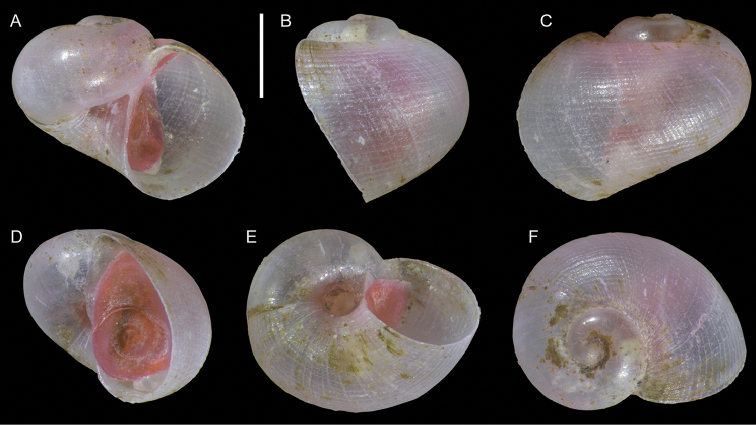
*Eunaticina papilla* (Gmelin, 1791), juvenile, Ashdod, Israel, APM DAN project (sample 8C): front (**A**), side (**B**) and back (**C**) views, operculum (**D**), base (**E**) and apical view (**F**). The pink hue is due to staining with eosin solution. Scale bar: 0.5 mm.

We here report the finding of a living individual of *Eunaticina papilla* from the Israeli Mediterranean shelf. This juvenile specimen can be assigned to *E. papilla* because of its overall shape, the sculpture of fine spiral cords, the large umbilicus and the morphology of the thin corneus operculum (Figure 5D).The species has already been reported in the Mediterranean Sea from Iskenderun in eastern Turkey with a living individual (Öztürk and Bitlis Bakir 2013). An empty shell was collected near Shiqmona, Israel, in November 2019 and reported as *Eunaticina linneana* (Récluz, 1843) (Schechter and Mienis 2020), a name considered a junior synonym of *E. papilla* by Beu et al. (2004).

#### Family Triphoridae Gray, 1847

##### Coriophora lessepsiana

Taxon classificationAnimaliaTriphoridae

Albano, Bakker & Sabelli sp. nov.

AD8D2C73-23C3-5553-81D4-24D97C663872

http://zoobank.org/B6911508-20E6-43B2-A01C-694571AD60FE

Figure 6

###### Type material.

***Holotype*.** Egypt • sh; Sinai (Red Sea), Dahab, dive site “Blue Hole”; 28.572°N, 34.538°E; depth 4 m; 2017; H. Blatterer leg.; NHMW-MO-113282.

***Paratypes*.** Egypt • sh; Sinai (Red Sea), Dahab, dive site “Tigerhouse”; 28.567°N, 34.533°E; depth 7 m; 2015; H. Blatterer leg.; OLML LIEV 2019/70/1 (paratype 1) • sh; Sinai (Red Sea), Dahab, dive site “Caves”; 28.416°N, 34.456°E; depth 20 m; 2017; H. Blatterer leg.; MNHN-IM-2014-7546 (paratype 2)

Sudan • sh; Arous, ca 30 km N of Port Sudan; 19.90°N, 37.23°E; depth 25–30 m; 2–8 Apr. 1975; G. Spada leg.; MZUB 60254 (paratype 3).

###### Additional material examined.

Egypt • 1 sh; Sinai (Red Sea), Dahab, dive site “Caves”; 28.416°N, 34.456°E; depth unspecified; 2012; H. Blatterer leg. • 1 sh; same collecting data as for preceding; depth 15 m; 2015 • 1 sh; same collecting data as for preceding; depth 14 m; 2017 • 1 sh; same collecting data as for preceding; depth 18 m; 2017 • 1 sh (juv.); Sinai (Red Sea), Dahab, dive site “Blue Hole”; floor of cave in cliff face; 28.57°N, 34.54°E; depth 25 m; Oct. 1994; D. Korkos leg.; H. Dekker coll. reg. no. 22017.

###### Records from the Mediterranean Sea.

**Figure 6.**
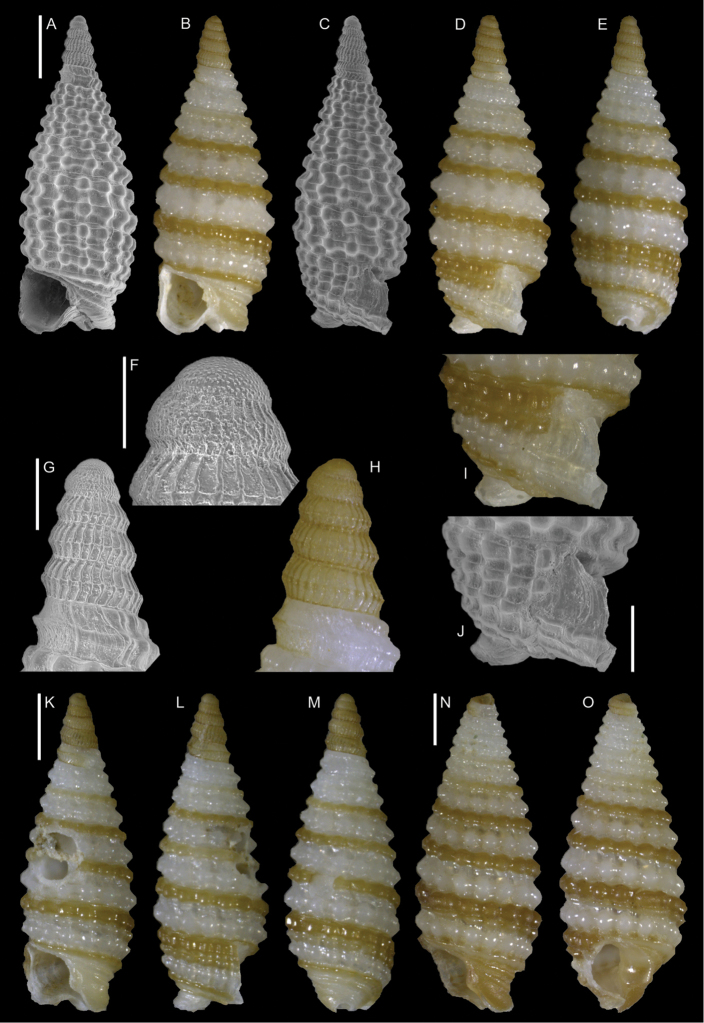
*Coriophora lessepsiana* Albano, Bakker & Sabelli, sp. nov. **A–J** holotype, NHMW-MO-113282, dive site “Blue Hole”, Dahab, Sinai, Egypt: front (**A, B**), side (**C, D**) and back (**E**) views, nucleus (**F**), protoconch (**G, H**) and peristome (**I, J**) **K, M** paratype 1, OLML LIEV 2019/70/1, dive site “Tigerhouse”, Dahab, Sinai, Egypt: front (**K**), side (**L**) and back (**M**) views. **N, O**NHMW-MO-112930/LM/0169, west of Rosh HaNikra Islands, Israel (Mediterranean), HELM project (sample S52_2M): front (**N**) and side (**O**) views. Scale bars: 0.5 mm (**A–E, K–O**); 0.1 mm (**F**); 0.2 mm (**G, H**); 0.3 mm (**I, J)**.

Israel • 1 sh; west of Rosh HaNikra Islands; 33.0704°N, 35.0926°E; depth 12 m; 29 Oct. 2018; HELM project (sample S52_2M); NHMW-MO-112930/LM/0169; size: H 3.1 mm, W 1.2 mm (illustrated shell, Figure 6N, O).

###### Diagnosis.

Shell cyrtoconoid of ~ 3 mm with 11 whorls and multispiral protoconch. Nucleus with hemispherical granules. Sculpture of three spiral cords of which two with elevated tubercles larger than their interspaces; second cord appearing later. Peristome apparently without bifurcating spiral cords.

###### Description.

***Color***: protoconch light brown; first teleoconch whorls whitish, with the first spiral cord becoming brown after one to three whorls. The second spiral cord acquires this brown color only on the dorsal part of the last whorl. The fourth cord, visible only on the last whorl, is brown. The base is light brown.

***Dimensions***: H 2.6 mm, W 1.0 mm (holotype); H 2.4 mm, W 0.9 mm (paratype 1); H 2.7 mm, W 1.1 mm (paratype 2, without apex); H 3.1 mm, W 1.2 mm (Mediterranean specimen, without apex).

***Protoconch***: multispiral with five whorls, H 530 µm (holotype), 553 µm (paratype 1).

Protoconch I: 1.5 whorls with hemispherical granules, nucleus height of 114 µm (holotype), 104 µm (paratype 1) and a maximum diameter of 154 µm (holotype), 145 µm (paratype 1).

Protoconch II: 3.5 monocarinated whorls with axial orthogonal riblets with a maximum diameter of 305 µm (holotype), 311 µm (paratype 1), 323 µm (paratype 2), 268 µm (Mediterranean specimen).

***Teleoconch***: 6 (holotype, paratype 1 and 2), 7.5 (Mediterranean specimen) whorls, height: 2.04 mm (holotype), 1.83 mm (paratype 1), 2.43 mm (paratype 2) and 2.95 mm (Mediterranean specimen).

The tuberculate first and third spiral cords start simultaneously after the protoconch with the same size, the third later becomes progressively larger and more acute. The second spiral cord appears only on the last whorl and is smaller than the others in front view, becoming of similar size to the first dorsally. In the second half of the last whorl, a very thin smooth suprasutural cord is visible. The base shows a fourth rather smooth cord of the same color as the first, followed by a fifth and sixth cord that are smooth and very pale in color. Anterior siphonal canal short, tubular, and oblique; posterior siphonal canal a simple notch. Peristome without microsculpture and apparently without bifurcating spiral cords.

The Mediterranean specimen is larger, has three white whorls after the protoconch and the second spiral cord appears on the seventh whorl, remaining still smaller than the others.

###### Etymology.

Named after the Lessepsian invasion (Por 1978), because we first found this Red Sea species on the Mediterranean Israeli shelf. The species epithet is an adjective in nominative singular feminine.

###### Remarks.

The Mediterranean specimen is larger and broader than the Red Sea ones. Triphorids do show a morphological dimorphism characterized by smaller and larger morphs and we think that we captured this dimorphism in our samples. See under *Opimaphora blattereri* Albano, Bakker & Sabelli, sp. nov. for a comparison with similar species.

##### Opimaphora blattereri

Taxon classificationAnimaliaTriphoridae

Albano, Bakker & Sabelli sp. nov.

85FFB108-E2A7-5BE2-B7BC-AD02FE80140C

http://zoobank.org/9133ECDF-5F41-4E68-80C2-D20A74549047

Figure 7

###### Type material.

***Holotype*.** Egypt • sh; Sinai (Red Sea), Dahab, dive site “Islands”; 28.476°N, 34.513°E; depth 10 m; 2015; H. Blatterer leg.; NHMW-MO-113283.

***Paratypes*.** Egypt • sh; same collecting data as for holotype; OLML LIEV 2019/70/2 (paratype 1) • sh; Sinai (Red Sea), Dahab, dive site “Labyrinth”; 28.478°N, 34.514°E; depth unspecified; 2011; H. Blatterer leg.; MNHN-IM-2014-7547 (paratype 2).

###### Additional material examined.

Egypt • 3 shs (juv. and fragments); same collecting data as for holotype • 9 shs (juv. and fragments); same collecting data as paratype 2 • 1 sh; Sinai (Red Sea), Dahab, dive site “Rick’s Reef”; 28.557°N, 34.524°E; 2012; H. Blatterer leg. • 1 sh; Sinai (Red Sea), Dahab, dive site “Caves”; 28.416°N, 34.456°E; depth 45 m; 2012; H. Blatterer leg. • 3 shs (juv.); Sinai (Red Sea), Dahab, dive site “Canyon”; 28.553°N, 34.522°E; depth 29 m; 2012; H. Blatterer leg. • 3 shs (juv.); Sinai (Red Sea), Dahab, Masbay Bay; 28.497°N, 34.518°E; depth 5 m; 2015; H. Blatterer leg. • 2 shs; Marsa Abu Makhadiq (Makadi Bay), SW side of bay, station 03; 26.9889°N, 33.9036°E; beached shell grit; 24 Sep. – 4 Oct. 1999; H. Dekker leg.; H. Dekker coll. reg. nos. 37192 and 37201).

**Figure 7.**
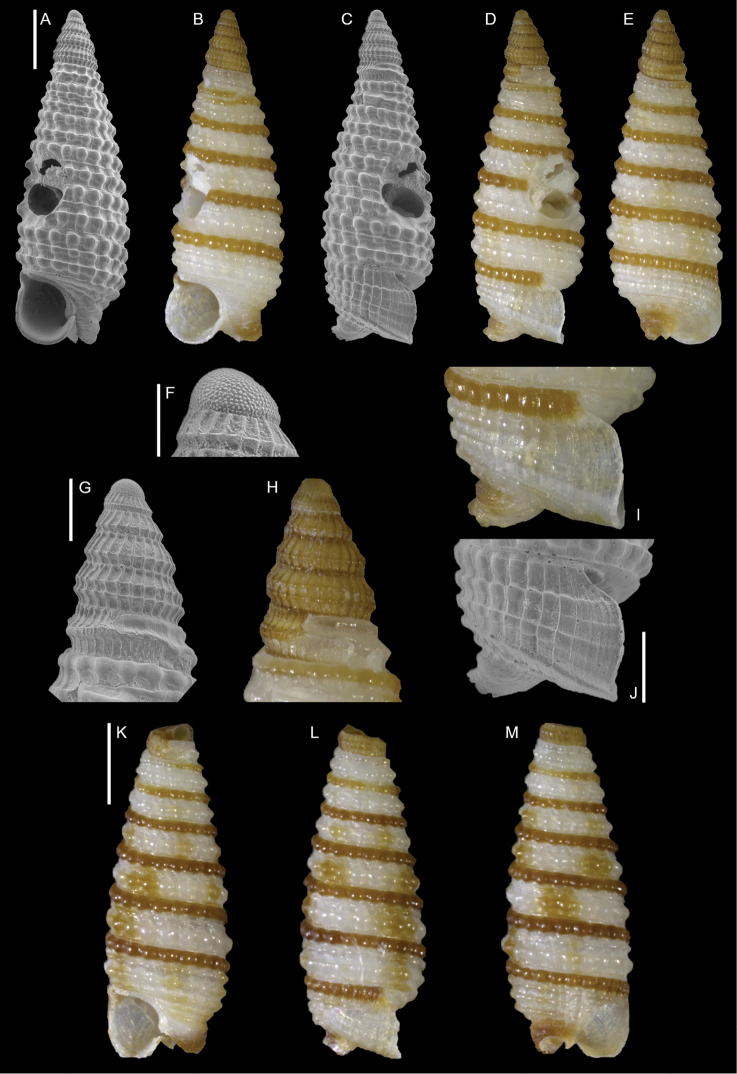
*Opimaphora blattereri* Albano, Bakker & Sabelli, sp. nov., dive site “Islands”, Dahab, Sinai, Egypt **A–J** holotype, NHMW-MO-113283: front (**A, B**), side (**C, D**) and back (**E**) views, nucleus (**F**), protoconch (**G, H**) and peristome (**I, J**) **K–M** paratype 1, LIEV 2019/70/2: front (**K**), side (**L**) and back (**M**) views. Scale bars: 0.5 mm (**A–E, K–M**), 0.1 mm (**F**); 0.2 mm (**G, H**); 0.3 mm (**I, J**).

###### Diagnosis.

Shell cyrtoconoid of less than 3 mm with 11 (holotype) or 12 (paratype 2) whorls and multispiral protoconch. Nucleus with hemispherical granules. Sculpture of three spiral cords with round tubercles larger than their interspaces; the second cord appears only on the fourth whorl, initially as a thin smooth thread. Microsculpture absent on the teleoconch whorls, present on the peristome, which bears bifurcating spiral cords.

###### Description.

***Color***: protoconch brown; whitish first teleoconch whorls with the first spiral cord becoming brown after two whorls. Light brown irregular patches are randomly distributed on the teleoconch, usually covering one or, more frequently, two tubercles. The base background is white, with the color patches of the last whorl extending onto it. The tip of the anterior siphon is brown.

***Dimensions***: H 2.7 mm, W 0.9 mm (holotype); H 2.6 mm, W 1.0 mm (paratype 1, without apex); H 3.0 mm, W 1.0 (paratype 2).

***Protoconch***: multispiral with 5.5 whorls (holotype), 5 (paratype 2, but first whorl worn); height: 566 µm (holotype), 644 µm (paratype 2).

Protoconch I: 1.5 whorls with hemispherical granules, nucleus with a height of 109 µm (holotype), 122 µm (paratype 2), and a maximum diameter of 371 µm (holotype), 380 µm (paratype 2).

Protoconch II: 3.5 whorls with axial orthogonal riblets with a maximum diameter of 371 µm (holotype), 380 µm (paratype 2). First two whorls monocarinated, then bicarinated.

***Teleoconch***: 6 (holotype), 7.5 (paratype 1), 7 (paratype 2) whorls, height: 2.24 mm (holotype), 2.43 mm (paratype 1), 2.50 mm (paratype 2).

The tuberculate first and third spiral cords start simultaneously after the protoconch with the same size, whereas the second cord appears from the fourth to the seventh teleoconch whorl, depending on shell size. This cord is initially thin and closer to the first one, it progressively increases its size until reaching that of the other two cords on the last whorl. On the second half of the shell, a very thin smooth suprasutural cord is visible. The second cord bifurcates on the peristome. The base shows a fourth rather smooth cord, and a fifth and sixth smooth ones; these cords become towards the peristome more granulated. On the peristome, below the third spiral thread, microsculpture is visible as fine spiral lines. Anterior siphonal canal tubular, short and oblique; posterior siphonal canal a simple notch.

###### Etymology.

This species is named after Hubert Blatterer, Austrian conchologist, in recognition of his work on Red Sea mollusks. Moreover, he contributed to our work on Lessepsian species by granting us access to the material he collected in the Red Sea and by donating the type series of *O. blattereri* and *Coriophora lessepsiana*. The species epithet is a noun in the genitive case.

###### Remarks.

We describe *O. blattereri* as new because of the similar color pattern to *C. lessepsiana* Albano, Bakker & Sabelli, sp. nov., even if it has not been reported from the Mediterranean Sea. The two species can be easily distinguished because *C. lessepsiana* has an monocarinated protoconch while *O. blattereri* has a bicarinated one; the second spiral cord of *O. blattereri* never becomes brownish as in *C. lessepsiana*; *O. blattereri* has a white background on the base and a distinct brown end of the anterior siphonal canal, whereas *C. lessepsiana* has a light brown base and the anterior siphon has not a colored end; the teleoconch of *O. blattereri* has irregular light brown patches, particularly evident on fresh specimens; this feature is totally absent in *C. lessepsiana*.

We have seen specimens very similar to *O. blattereri* collected in Madagascar, New Caledonia, and French Polynesia. A revision of the group in the Indo-Pacific province is beyond the scope of this paper; however, this species likely has a broad distribution.

*Opimaphora blattereri* and *C. lessepsiana* share their color pattern of brown to orange spiral cords on a white background with other Indo-Pacific species. *Litharium bilineatum* (Kosuge, 1962) (holotype illustrated by Higo et al. (2001)), *Costatophora iniqua* (Jousseaume 1898) (= *Notosinister kawamurai* Kosuge, 1962, type material illustrated by Higo et al. (2001) and Albano et al. (2019)) and *Aclophora albozonata* Laseron, 1958 can be easily distinguished by having three fully developed spiral cords since the early teleoconch. *Iniforis formosula* (Hervier, 1898) and *Mastonia peanites* Jousseaume, 1898 (= *Mastonia squamosa* Kosuge, 1962, type material again illustrated by Higo et al. (2001) and Albano et al. (2019)) have only two spiral cords, but the former has three or four dark brown lines on the last whorl, whereas the latter has a dark brown last whorl with lighter tubercles. *Triphora fulvescens* Hervier, 1898 also has a similar color pattern, but the second spiral cord remains a very fine thread even on the last whorl and the tubercles are whitish even on the first cord (on an orange background). Some species show a delayed appearance of the second spiral cord: *Nototriphora regina* (Hedley, 1903) has a brown tip of the anterior siphonal canal similarly to *O. blattereri*, but lacks the patches on the whorls and has an orange line on the third spiral cord on the last whorl; *Coriophora tigris* Laseron, 1958 has a paucispiral protoconch; *Cautor similis* (Pease, 1871) has larger and more densely arranged tubercles, a brown fourth spiral cord and white base. Last, a few species have a similar color pattern, but with an inverted pattern: the first spiral cord is white and the third orange to brown, like *Mastonia cingulifera* (Pease, 1861), which also has a dark yellow teleoconch, *Mastonia funebris* Jousseaume, 1884 and *Mastonia tulipa* Jousseaume, 1898 with a brown and white base, respectively.

##### Triphora

Taxon classificationAnimaliaTriphoridae

sp.

23572EBB-608D-54B7-922A-959A4D532918

Figure 8

###### New records.

Israel • 1 sh; north of Atlit; 32.7433°N, 34.9067°E; depth 40 m; 20 Sep. 2016; coarse biogenic sediment in a pool among rocks covered by coralligenous formations; grab; HELM project (sample NG40_2M); NHMW-MO-112930/LM/0170; size: H 4.5 mm, W 1.4 mm.

###### Remarks.

We found a single, adult, empty shell. It likely possesses a large paucispiral protoconch, but it is incomplete in our shell. The second spiral cord starts at mid-shell height, the fourth and fifth spiral cords are smooth, and the posterior siphonal canal is shallow. It is brown in color with darker spiral cords. We have not been able to assign it to a species so far, but it is distinctly different from all known Mediterranean species and most likely belongs to the Indo-Pacific fauna.

**Figure 8.**
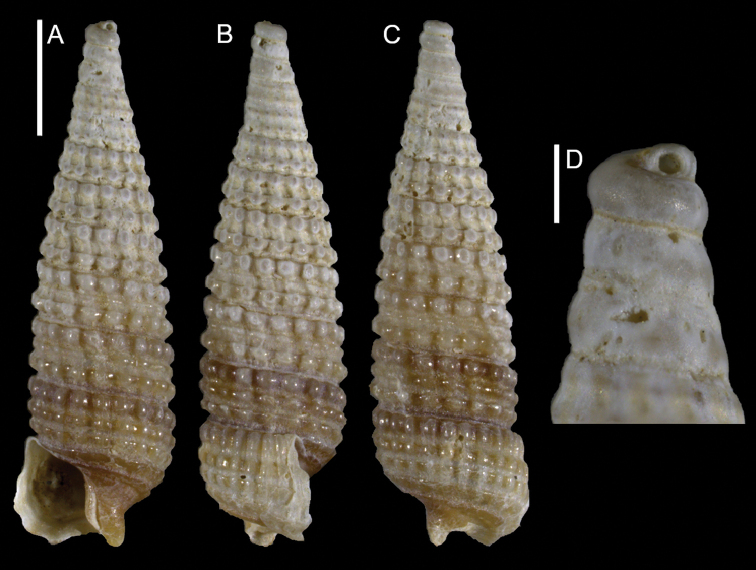
*Triphora* sp., NHMW-MO-112930/LM/0170, north of Atlit, Israel, HELM project (sample NG40_2M): front (**A**), side (**B**) and back (**C**) views, protoconch (**D**). Scale bars: 1 mm (**A–C**); 0.2 mm (**D**).

##### Viriola cf. bayani

Taxon classificationAnimaliaTriphoridae

Jousseaume, 1884

21005B41-6C3F-5976-B9FC-B8E324B56A91

###### New records.

Israel • 3 spcms; Ashqelon; 31.6868°N, 34.5516°E; depth 11 m; 31 Oct. 2018; offshore rocky reef; suction sampler; HELM project (samples S58_1M, S58_2F, S58_2M) • 3 spcms; off Tel Aviv Marina; 32.0871°N, 34.7635°E; depth 7 m; 8 Nov. 2018; rocky reef, suction sampler; HELM project (sample S67_1M) • 7 spcms; west of Rosh HaNikra Islands; 33.0704°N, 35.0926°E; depth 12 m; rocky substrate; suction sampler; 29 Oct. 2018; HELM project (samples S52_1M, S52_3F) • 1 spcm; west of Rosh HaNikra Islands; 33.0725°N, 35.0923°E; depth 20 m; 1 May 2018; rocky substrate; suction sampler; HELM project (sample S13_3M) • 9 spcms; same collecting data as for preceding; depth 19 m; 29 Oct. 2018; HELM project (samples S53_1M, S53_2F, S53_2M, S53_3F, S53_3M); size of largest specimen: H 11.6 mm, W 2.7 mm.

###### Remarks.

This species was first recorded from Israel by Steger et al. (2018) based on three living individuals from Palmachim, southern Israel. We here report multiple living individuals all along the Israeli coast, confirming its establishment. The species shows a broad distribution in the Eastern Mediterranean ranging from Greece to Turkey and Cyprus (Micali et al. 2017; Stamouli et al. 2017; Angelidis and Polyzoulis 2018; Chartosia et al. 2018). Its final taxonomic assignment requires the clarification of the relation between several other *Viriola* such as *V. corrugata* (Hinds, 1843), *V. senafirensis* (Sturany, 1903), and *V. tricincta* (Dunker, 1882) (Albano and Bakker 2016; Albano et al. 2017, 2019).

#### Family Cerithiopsidae H. Adams & A. Adams, 1853

##### Cerithiopsis sp. aff. pulvis

Taxon classificationAnimaliaCerithiopsidae

(Issel, 1869)

36E8B960-BB9B-5AE0-A472-1239BF4BFC21

Figure 9D–F

###### New records.

Israel • 1 sh; Ashqelon; 31.6868°N, 34.5516°E; depth 11 m; 31 Oct. 2018; offshore rocky reef; suction sampler; HELM project (sample S58_1F) • 1 spcm; Ashqelon; 31.6891°N, 34.5257°E; depth 28 m; 31 Oct. 2018; offshore rocky reef; suction sampler; HELM project (sample S59_3F); NHMW-MO-112930/LM/0174; size: H 1.8 mm, W 0.7 mm (illustrated specimen).

###### Additional material examined.

*Cerithiopsis pulvis* (Issel, 1869): Israel • 2 spcms, 1 sh; Ashqelon; 31.6868°N, 34.5516°E; depth 12 m; 30 Apr. 2018; offshore rocky reef; suction sampler; HELM project (sample S12_1F) • 5 spcms; same collecting data as for preceding; depth 11 m; 31 Oct. 2018; HELM project (samples S58_1F, S58_1M, S58_2F) • 5 spcms, 2 shs; Ashqelon; 31.6891°N, 34.5257°E; depth 25 m; 2 May 2018; offshore rocky reef; suction sampler; HELM project (samples S16_1F, S16_2F, S16_2M) • 19 spcms; same collecting data as for preceding; depth 28 m; 31 Oct. 2018; HELM project (samples S59_1F, S59_1M, S59_2F, S59_3F) • 8 spcms; west of Rosh HaNikra Islands; 33.0704°N, 35.0926°E; depth 12 m; 29 Oct. 2018; rocky substrate; suction sampler; HELM project (samples S52_2F, S52_2M, S52_3M) • 1 spcm; west of Rosh HaNikra Islands; 33.0725°N, 35.0923°E; depth 20 m; 1 May 2018; rocky substrate; suction sampler; HELM project (sample S13_1M) • 6 spcms; same collecting data as for preceding; depth 19 m; 29 Oct. 2018; HELM project (samples S53_1F, S53_1M, S53_3F).

###### Remarks.

This species superficially resembles the Lessepsian *Cerithiopsis pulvis* but has a more cyrtoconoid shape and a greater ratio between the height of the last whorl and that of the shell. The base is not concave as in *C. pulvis*, bears a fourth spiral cord which is more prominently tuberculate, and an additional fifth tuberculate cord that is not present in typical *C. pulvis*. Additionally, the siphonal canal bears numerous fine cords. The color pattern is similar to *C. pulvis* which has orange bands on white background; in contrast, in C. aff. pulvis these are brown and yellowish, respectively. It is distinct from any native Mediterranean species and clearly belongs to an Indo-Pacific clade. It is here considered a new non-indigenous species.

**Figure 9.**
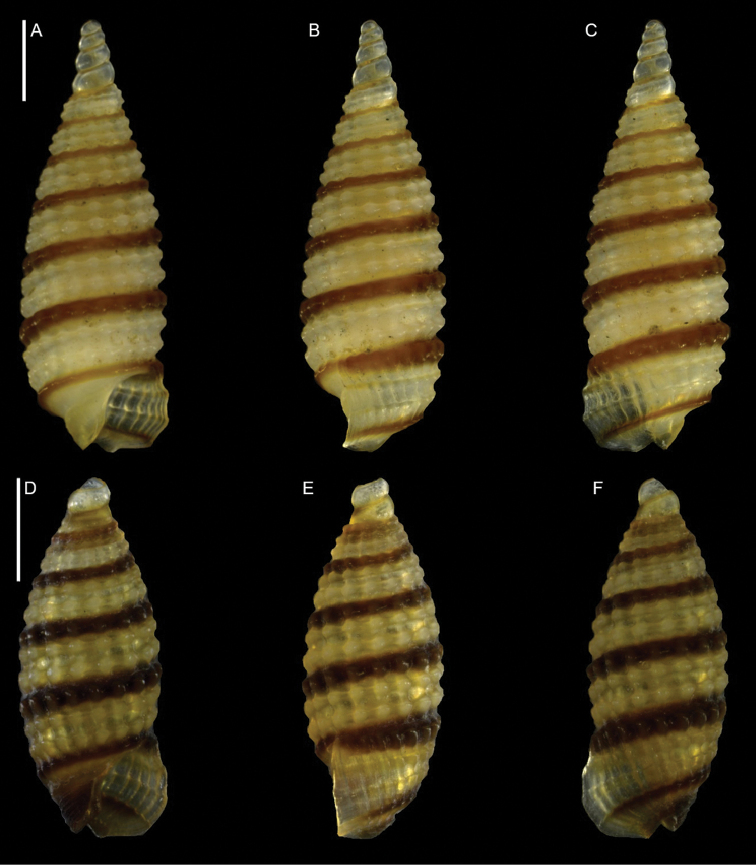
Comparison between *Cerithiopsis pulvis* (Issel, 1869) and Cerithiopsis sp. aff. pulvis. **A–C***Cerithiopsis pulvis*, Ashqelon, Israel, HELM project (sample S16_1F): front (**A**), side (**B**) and back (**C**) views. **D–F**Cerithiopsis sp. aff. pulvis, NHMW-MO-112930/LM/0174, Ashqelon, Israel, HELM project (sample S59_3F): front (**D**), side (**E**) and back (**F**) views. Scale bars: 0.5 mm.

##### Cerithiopsis

Taxon classificationAnimaliaCerithiopsidae

sp.

52CDA653-5BBA-502A-A88F-5E839FA47CA0

Figure 10

###### New records.

Israel • 1 sh; Shiqmona Beach; 32.8259°N, 34.9555°E; beached; 4 Jan. 2008; size: H 3.5 mm, W 1.2 mm.

###### Remarks.

This beautiful species has almost eight teleoconch whorls bearing two strong spiral cords with oblong tubercles at the intersection with prosocline axial ribs. Interspaces between spiral cords are approximately as large as the cords themselves, and interspaces between the axial ribs are double the size of the ribs. A third smooth thick cord delimits the rather flat base and is visible above the suture throughout most of the teleoconch. The protoconch is smooth with very fine and extremely short axial riblets just below the suture; it is multispiral but broken in our specimen in which only the last two whorls are preserved. The slender shape, the two strong spiral cords and the smooth flat base distinguish it at once from all native Mediterranean species suggesting it is a new non-indigenous species in the basin.

Among Indo-Pacific cerithiopsids, *Synthopsis lauta* Cecalupo & Perugia, 2013, described from Vanuatu, is among the few similar species we were able to trace. However, the interspace between the spiral cords is broader, the tubercles on the first spiral cord of the last whorl are larger than those on the second cord, and the teleoconch is shorter with just six whorls. Additionally, the color pattern with white tubercles, yellowish interspaces, deep brown suture and violet protoconch is strikingly different from the one of our shell. We have some reservations that *S. lauta*, as well as our specimen, belong to the genus *Synthopsis* Laseron, 1956 that was described as bearing three tuberculate spiral cords on the whole teleoconch (Laseron 1956). Pending a molecular phylogeny of the family, we consider this feature important at the genus level. Therefore, we assign our specimen to the nominotypical genus *Cerithiopsis*, in the wait of a better understanding of cerithiopsid systematics. The specimen identified as *Horologica gregaria* Cecalupo & Perugia, 2012 and illustrated in the recent revision of Cerithiopsidae from South Madagascar (Cecalupo and Perugia 2014b: fig. 8G) is also similar to ours; that specimen, however, has a distinct basal spiral cord which is absent in our specimen. The latter character, the prominence of the tuberculate spiral cords and the evident but rather flat third cord also raise some doubts that the specimen from South Madagascar is conspecific with the *H. gregaria* originally described from the Central Philippines (Cecalupo and Perugia 2011). Last, the Sudanese specimen of Horologica cf. taeniata Cecalupo & Perugia, 2013 illustrated by Cecalupo and Perugia (2016: fig. 1P–S) shares the general features of our shell but can be distinguished by the first spiral cord that tends to split into two separate cords, and by the color pattern of white teleoconch and orange base.

**Figure 10.**
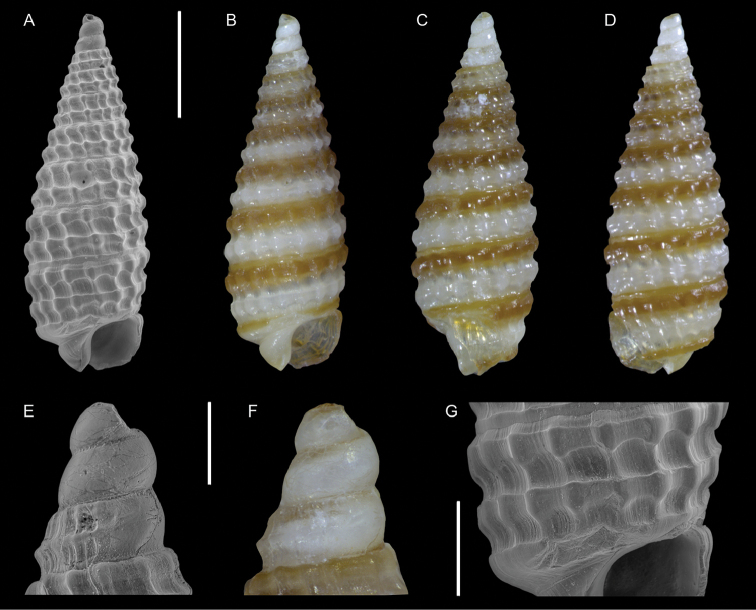
*Cerithiopsis* sp., Shiqmona Beach, Israel: front (**A, B**), side (**C**) and back (**D**) views, protoconch oriented to highlight the transition to the teleoconch (**E, F**), microsculpture (**G**). Scale bars: 1 mm (**A–D**); 0.2 mm (**E, F**); 0.4 mm (**G**).

##### Joculator problematicus

Taxon classificationAnimaliaCerithiopsidae

Albano & Steger sp. nov.

393126D6-44DA-58D3-A51E-AFDC55B446C7

http://zoobank.org/1D9DDB2C-99D0-40A6-824A-93F4149B3550

Figure 11

###### Type material.

***Holotype*.** Israel • spcm; Ashqelon; 31.6868°N, 34.5516°E; depth 11 m; 31 Oct. 2018; offshore rocky reef; suction sampler; HELM project (sample S58_3F); NHMW-MO-113580.

***Paratypes*.** Israel • spcm; west of Rosh HaNikra Islands; 33.0704°N, 35.0926°E; depth 12 m; 1 May 2018; rocky substrate; suction sampler; HELM project (sample S14_2F); MNHN-IM-2012-25505 (paratype 1) • spcm; same collecting data as for paratype 1; HELM project (sample S14_4F); MZUB 60400 (paratype 2) • spcm; same collecting data as for paratype 1; 29 Oct. 2018; HELM project (sample S52_3F); SMF 360591 (paratype 3) • spcm; same collecting data as for paratype 1; HELM project (sample S14_2F); SMNH MO 99705 (paratype 4).

###### Additional material examined.

Israel • 5 spcms; Ashqelon; 31.6868°N, 34.5516°E; depth 12 m; 30 Apr. 2018; offshore rocky reef; suction sampler; HELM project (samples S12_1F, S12_2F, S12_3F) • 6 spcms; same collecting data as for preceding; depth 11 m; 31 Oct. 2018; HELM project (samples S58_1F, S58_2F, S58_3F) • 1 spcm; Ashqelon; 31.6891°N, 34.5257°E; depth 25 m; 2 May 2018; offshore rocky reef; suction sampler; HELM project (sample S16_2F) • 2 spcms; same collecting data as for preceding; depth 28 m; 31 Oct. 2018; HELM project (samples S59_3F, S59_3M) • 1 sh; Sdot Yam; 32.5299°N, 34.8599°E; depth 24 m; 3 May 2018; rocky substrate; suction sampler; HELM project (sample S17_1F) • 3 spcms; west of Rosh HaNikra Islands; 33.0704°N, 35.0926°E; depth 12 m; 1 May 2018; rocky substrate; suction sampler; HELM project (samples S14_2F, S14_4F) • 16 spcms; same collecting data as for preceding; 29 Oct. 2018; HELM project (samples S52_1F, S52_1M, S52_2F, S52_3F) • 2 spcms; west of Rosh HaNikra Islands; 33.0725°N, 35.0923°E; depth 20 m; 1 May 2018; rocky substrate; suction sampler; HELM project (sample S13_1F) • 9 spcms; same collecting data as for preceding; depth 19 m; 29 Oct. 2018; HELM project (samples S53_1F, S53_2F, S53_3F, S53_3M).

**Figure 11.**
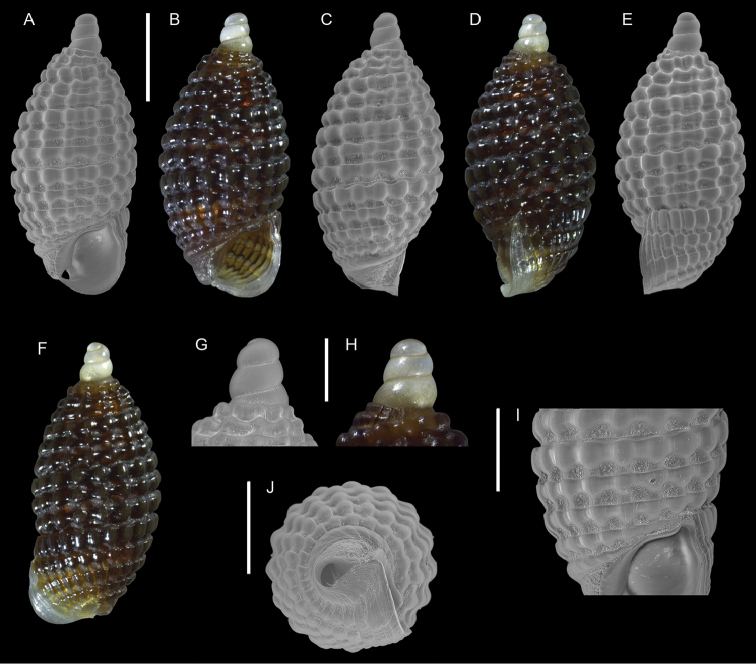
*Joculator problematicus* Albano & Steger, sp. nov., holotype, NHMW-MO-113580, Ashqelon, Israel, HELM project (sample S58_3F): front (**A, B**), left side (**C**), right side (**D, E**) and back (**F**) views, protoconch (**G, H**), microsculpture (**I**) and base and siphonal canal (**J**). Scale bars: 0.5 mm (**A–F**); 0.2 mm (**G, H**); 0.3 mm (**I**); 0.4 mm (**J**).

###### Diagnosis.

Very small bulbous brown shell, of ~ 1.5 mm in height and < 1 mm in width, with a relatively short, almost smooth protoconch.

###### Description.

***Color***: Protoconch white, teleoconch brown with white outer lip margin.

***Dimensions***: H 1.6 mm, W 0.7 mm (holotype), H 1.4 mm, W 0.7 mm (paratype 1), H 1.5 mm, W 0.7 mm (paratype 2), H 1.5 mm, W 0.7 mm (paratype 3), H 1.6 mm, W 0.8 mm (paratype 4).

***Protoconch***: composed of 3.5 whorls with no clear demarcation between protoconchs I and II, height: ~ 300 µm, width ~ 200 µm (holotype), but accurate measurement hampered by the last protoconch whorl being covered by the first teleoconch whorl. It appears smooth except for growth lines and fine pustules covering the lower half of the first whorl and sparsely present apically and abapically on the following whorls (only visible with scanning electron microscopy at high magnification).

***Teleoconch***: 4 whorls (holotype), height: 1.4 mm (holotype). It bears three spiral cords of equal size, with tubercles at the intersection of orthocline axial ribs. The base is contracted and has two additional tuberculate spiral cords. Tubercles become oblong near the lip. Anterior siphonal canal short, reverted upwards, formed by a prong-like protrusion of the anterior outer lip (Figure 11A); posterior siphonal canal notch-like.

###### Etymology.

The name *problematicus* refers to the difficult task of recognizing and identifying non-indigenous species belonging to groups whose taxonomy in the tropical seas is poorly known (see Discussion). The species epithet is an adjective in nominative singular masculine.

###### Remarks.

This species is characterized by its bulbous contour and constricted last whorl which justify its inclusion in the genus *Joculator* Hedley, 1909 (Hedley 1909; Marshall 1978).

The Cerithiopsidae of the Indo-Pacific have been subject to numerous in-depth studies (Cecalupo and Perugia 2011, 2013, 2014a, b, 2016, 2017a, b, 2018, 2019a, b, c). Still, this species does not fit any of the known species. Among the most similar species in terms of shell shape and ornamentation, *Joculator itiensis* Cecalupo & Perugia, 2014 has one teleoconch whorl more and a different color pattern characterized by light brown first whorl and base, *J. olivoideus* Cecalupo & Perugia, 2018 can be distinguished by its clearly prosocline axial ribs and greyish tubercles, and *J. sekensis* Cecalupo & Perugia, 2018 has only two spiral cords and blunter axial ribs on the first teleoconch whorl, in addition to a blunter siphonal canal.

There are several more species of small brown bulbous *Joculator* often distinguishable only by subtle character differences. *Joculator priorai* Cecalupo & Perugia, 2012 is corneous in color and has a pointed protoconch with one additional whorl; moreover, in our specimens the interspaces between the spiral cords are smaller. *Joculator pupiformis* Cecalupo & Perugia, 2012 has one protoconch and one teleoconch whorl more, the tubercles are oblong, and the base lacks a clearly visible fifth tuberculate spiral cord. *Joculator fuscus* Cecalupo & Perugia, 2012 has much broader interspaces between cords and a wide subquadrangular aperture which is, in contrast, quite small in our specimens. *Joculator furvus* Cecalupo & Perugia, 2012 has a neat abapical smooth cord on the protoconch, one teleoconch whorl less and a broader aperture. *Joculator carpatinus* Cecalupo & Perugia, 2012 has one protoconch whorl more, one teleoconch whorl less, a broader aperture and a fine abapical thread on the protoconch. *Joculator caliginosus* Cecalupo & Perugia, 2012 has one protoconch whorl more and one teleoconch whorl less, the basal fourth and fifth cords are only weakly tuberculate whereas they are neatly tuberculate in our specimens. *Joculator coffeus* and *J. subglobosus*, both Cecalupo & Perugia, 2013, have one clear abapical thread on the protoconch, one teleoconch whorl less, the shell has a more roundish shape and the lip does not reach anteriorly the siphonal canal, almost covering it, like in our specimens. The other representatives of *Joculator* include also other more elongated species that can be easily distinguished from our specimens.

This species is superficially similar to the native Mediterranean *Cerithiopsis ladae* Prkić & Buzzurro, 2007, which, however, can be distinguished at once for not having the last protoconch whorl partially covered by the first teleoconch whorl and lacking the prong-like process of the anterior outer lip. Additionally, tubercles in *C. ladae* on the last whorl are more elongated, subrectangular, and the shell profile is less bulbous. *Cerithiopsis greppii* Buzzurro and Cecalupo, 2005, described from Turkey, has a rather oval profile, but not as bulbous as in our species; additionally, it has a paucispiral protoconch. *Cerithiopsis micalii* (Cecalupo and Villari, 1997), which also has a somewhat oval shell profile, can be quickly distinguished by its protoconch whose last two whorls bear strong axial ribs.

Unfortunately, a revision of Red Sea Cerithiopsidae is lacking, but given that *Joculator* is a broadly distributed genus in the Indo-Pacific province, we consider *J. problematicus* another previously undescribed Indo-Pacific species recently introduced to the Mediterranean Sea.

#### Family Elachisinidae Ponder, 1985

##### Elachisina

Taxon classificationAnimaliaLittorinimorphaElachisinidae

sp.

5ED182D4-D891-570A-9C63-AF53C4867A6F

Figure 12A–E

###### New records.

Israel • 1 spcm; north of Atlit; 32.7417°N, 34.9177°E; depth 31 m; 25 Apr. 2017; sand; grab; HELM project (sample NG30_8M) • 14 spcms, 1 sh; west of Rosh HaNikra Islands; 33.0725°N, 35.0923°E; depth 20 m; 1 May 2018; rocky substrate; suction sampler; HELM project (samples S13_1F, S13_1M, S13_2F, S13_3L); size of largest specimen: H 1.6 mm, W 1.3 mm • 9 spcms; same collecting data as for preceding; depth 19 m; 29 Oct. 2018; HELM project (samples S53_1F, S53_2F, S53_3F).

###### Additional material examined.

*Elachisina robertsoni* Kay, 1979: United States • 1 sh; Hawaii, Oahu, Maunalua Bay; BPBM 9754 (holotype).

###### Remarks.

The morphology of this species is unique among the native mollusks of the Mediterranean, which does not host any shallow water Elachisinidae. Therefore, we consider it a new non-indigenous species in the basin.

The only Indo-Pacific *Elachisina* we are aware of is *E. robertsoni* Kay, 1979, which indeed shares the general characters of our species. However, it can be readily distinguished by the thicker and fewer spiral cords, less rounded whorls and sigmoid, rather than strongly prosocline, aperture profile. *Elachisina* sp. is more similar to the West-African *E. tenuisculpta* (Rolán and Gofas 2003), but the Israeli shells have more rounded whorls, a greater height/width ratio and smaller ratio between aperture and shell height.

**Figure 12.**
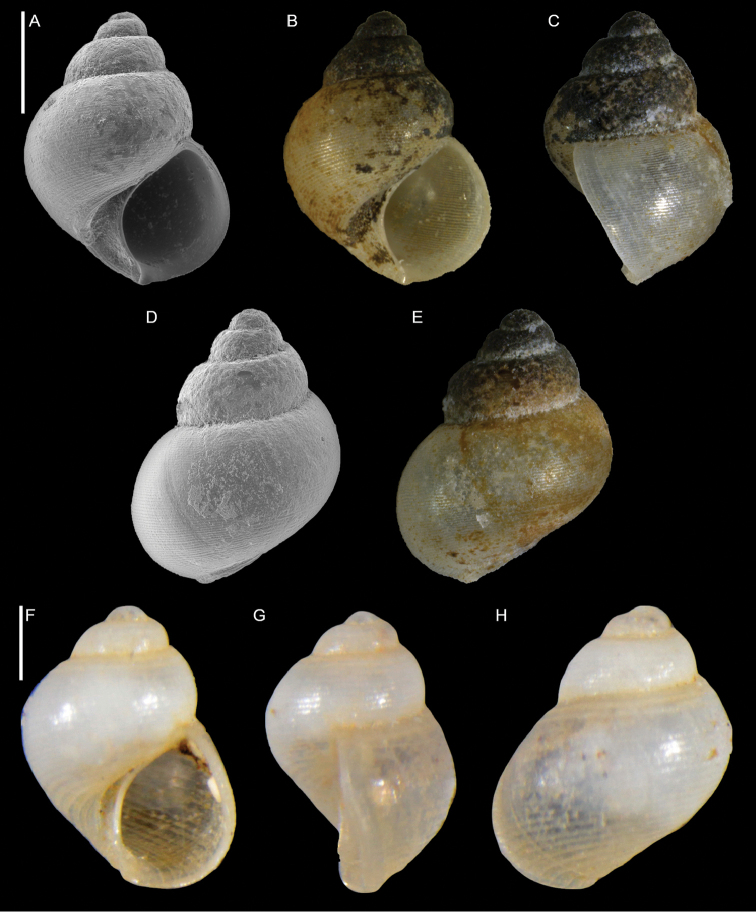
Comparison between *Elachisina* sp. and *Elachisina robertsoni* Kay, 1979 **A–E***Elachisina* sp., west of Rosh HaNikra Islands, Israel, HELM project (sample S13_3L): front (**A, B**), side (**C**) and back (**D, E**) views **F–H***Elachisina robertsoni*, BPBM 9754 (holotype), Maunalua Bay, Oahu, Hawaii: front (**F**), side (**G**) and back (**H**) views (photograph courtesy N. Young). Scale bars: 0.5 mm.

#### Family Iravadiidae Thiele, 1928

##### Iravadia aff. elongata

Taxon classificationAnimaliaIravadiidaeIravadiidae

(Hornung & Mermod, 1928)

FE1FFC38-E077-57C4-A7E0-D91D240FFC63

Figure 13

###### New records.

Israel • 1 spcm; Ashqelon; 31.6868°N, 34.5516°E; depth 12 m; 30 Apr. 2018; offshore rocky reef; suction sampler; HELM project (sample S12_1F) • 5 shs; same collecting data as for preceding; depth 11 m; 31 Oct 2018; HELM project (sample S58_2F); size: H 2.8 mm, W 1.1 mm (largest (illustrated) shell).

###### Additional material examined.

Iravadia aff. elongata: Sudan • 2 shs; Arusa (near Port Sudan); 19.90°N, 37.23°E; shallow water; 1975; shell-grit; G. Spada leg.; MZUB.

*Iravadia elongata* (Hornung & Mermod, 1928): Eritrea • 1 sh; Massawa; depth 30 m; 1870; A. Issel leg.; syntype in MSNG; size: H 3.9 mm, W 1.4 mm.

###### Remarks.

**Figure 13.**
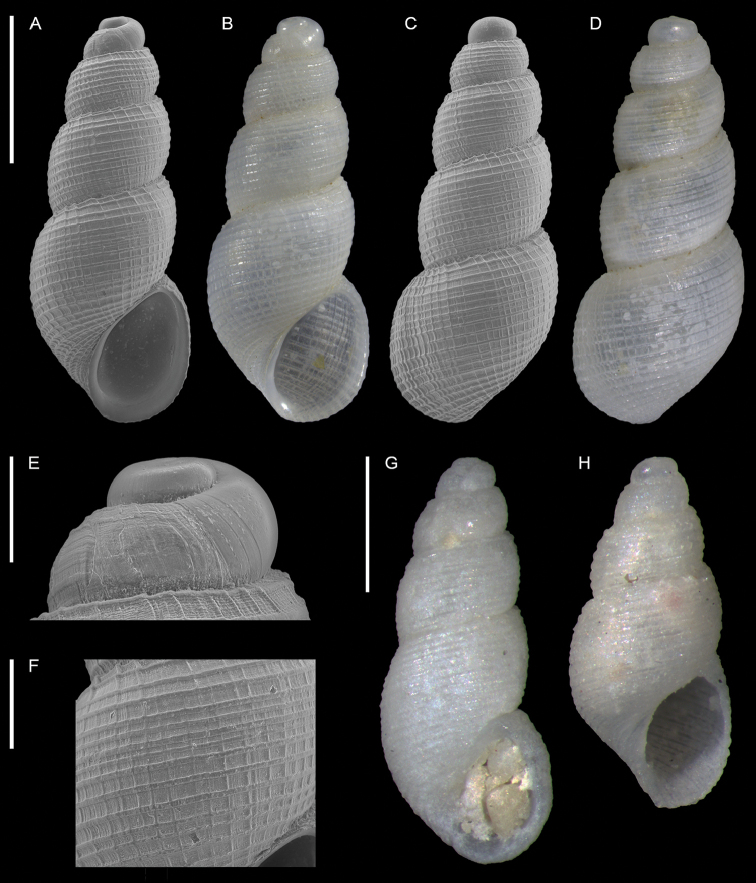
Iravadia aff. elongata (Hornung & Mermod, 1928) **A–F** Ashqelon, Israel, HELM project (sample S58_2F): front (**A, B**) and back (**C, D**) views, protoconch (**E**) and detail of sculpture on the body whorl (**F**) **G, H** Arusa (near Port Sudan), Sudan: front view of an adult (**G**) and juvenile (**H**) shell. Scale bars: 1 mm (**A–D, G, H**); 0.2 mm (**E**); 0.3 mm (**F**).

This species is characterized by a turriform shell with up to five convex whorls, separated by a marked suture and a blunt, flat, and smooth protoconch. The sculpture consists of flat spiral ridges (12–14 on the penultimate whorl) that become more raised at both the adapical and abapical parts of the whorls, and which overlie numerous axial lines (Figure 13F), resulting in a reticulate surface.

The closest match to our specimens is *Iravadia elongata* (Hornung & Mermod, 1928) which was described from material collected by Arturo Issel in the Red Sea off Massawa, Eritrea, at 30 m depth (Hornung and Mermod 1928). Compared to our material, however, the syntype of *I. elongata* is larger (height 3.9 mm vs. 2.8 mm in our largest shell) and has seven less convex whorls. Further, the apical part of its spire has a slightly concave profile and thus appears more tapered. According to Issel’s description, the sculpture of *I. elongata* consists of spiral ridges (12 on the penultimate and 22 on the last whorl) as well as growth lines, although the latter are not indicated in the accompanying line drawing. This suggests that the axial component might be less evident in *I. elongata* than in our specimens, however, the poor preservation of the shell surface of the syntype of *I. elongata* did not allow a reliable comparison with our material. Slightly eroded shells very similar to our specimens have been collected from the Sudanese Red Sea (Figure 13G, H), confirming that the material from Israel indeed represents an Indo-Pacific species rather than an undescribed Mediterranean taxon.

Among Mediterranean iravadiids, our specimens superficially resemble only *Ceratia proxima* (Forbes and Hanley, 1850). This species, however, lacks axial sculpture. Interestingly, Hornung and Mermod (1928) also mention the presence of this latter species at Assab (Eritrea) and “île Saldadin” (Zeila, northern Somalia). While obviously based on a misidentification – *C. proxima* has an Eastern Atlantic-Mediterranean distribution (Bouchet and Warén 1993; Høisæter 2009) – one might speculate that this record could be the result of a confusion of *C. proxima* with the *Iravadia* presented here.

#### Family Vitrinellidae Bush, 1897

##### Vitrinella aff. Vitrinella

Taxon classificationAnimaliaLittorinimorphaVitrinellidae

sp. 1 (sensu Blatterer 2019)

3D061CAF-0549-519F-9658-40B022B97FF3

Figure 14

###### New records.

Israel • 1 sh; Ashdod; 31.8697°N, 34.6473°E; depth 24 m; Sep. 2019; soft substrate; grab; APM DAN project (sample 10B); size: H 0.4 mm, W 0.7 mm.

###### Remarks.

**Figure 14.**
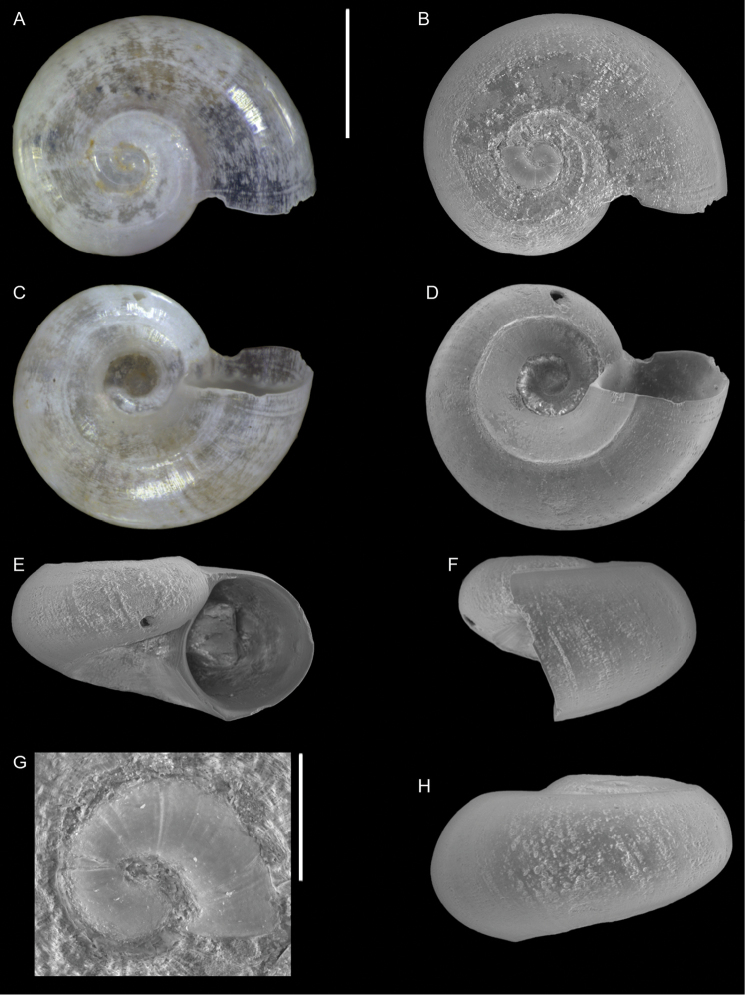
*Vitrinella* aff. *Vitrinella* sp. 1 (sensu Blatterer 2019), Ashdod, Israel, APM DAN project (sample 10B): apical (**A, B**), umbilical (**C, D**), front (**E**) and side (**F**) views, protoconch (**G**) and back view (**H**). Scale bars: 0.3 mm (**A–F, H**); 0.1 mm (**G**).

This tiny gastropod defeated all our attempts to identify it. It consists of a protoconch and a teleoconch of ~ 1.5 whorls each. Sculpture is absent, except for two spiral ridges that run on the shoulder and on the base. A third ridge runs periumbilically (Figure 14E). Broad umbilicus, roundish aperture. Our shell closely resembles the *Vitrinella* sp. 1 illustrated by Blatterer (2019: plate 127, fig. 12a–j) from the Dahab region in the northern Red Sea, which, however, apparently bears fine spiral threads in the umbilicus (fig. 12e, and unpublished figures). SEM images of our shell show that its surface is taphonomically altered; additionally, Blatterer’s specimens look slightly more mature, reaching 2 teleoconch whorls. The significance of these features should be re-assessed upon a satisfying revision of these tiny gastropods from the Indo-Pacific province. Another similar shell is illustrated by Janssen et al. (2011, plate 19, figs. 3a–b), which apparently has less conspicuous or absent spiral ridges as long as can be judged from the optical illustrations provided. It is worth mentioning that gastropods belonging to the family Clenchiellidae D.W. Taylor, 1966 share the small size, low spire, wide umbilicus and presence of strong spiral keels we observed in our specimen (Ponder et al. 2014); the latter, however, lacks the numerous finer spiral cords that characterize clenchiellids. Additionally, these gastropods occur in mangrove swamps or adjacent habitats in tropical estuaries, a kind of habitat that does not occur in Israel. The shell shape and sculpture (in particular the strong spiral keels) distinguish it at once from native Mediterranean species. The extreme similarity with the shell illustrated in Blatterer’s book suggests that the species belongs to a Red Sea clade and is here considered a new non-indigenous species in the Mediterranean Sea.

#### Family Eulimidae Philippi, 1853

##### Hypermastus

Taxon classificationAnimaliaLittorinimorphaEulimidae

sp.

D0C4DB3B-E3BA-5D1F-9C17-EAECFC337221

Figure 15

###### New records.

Israel • 4 shs; off Tel Aviv Marina; 32.0871°N, 34.7635°E; depth 7 m; 8 Nov. 2018; rocky reef; suction sampler; HELM project (sample S67_3F); size: H 1.9 mm, L 0.5 mm (illustrated shell).

###### Remarks.

**Figure 15.**
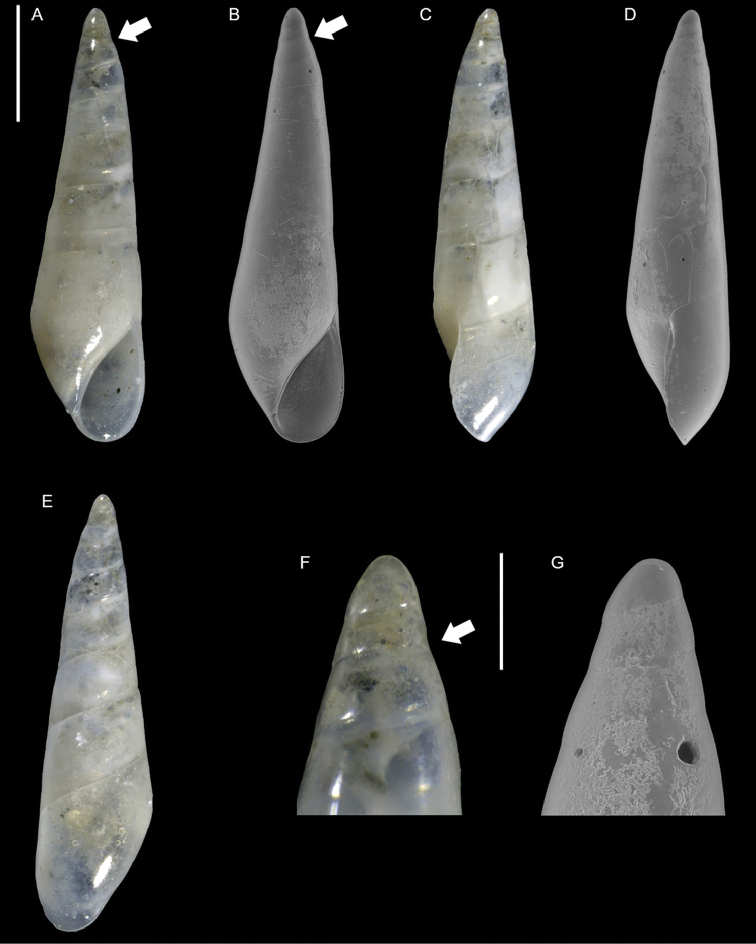
*Hypermastus* sp., Tel Aviv, Israel, HELM project (sample S67_3F): front (**A, B**), side (**C, D**) and back (**E**) views, apex with optical microscope (**F**) and SEM (**G**) showing the protoconch-teleoconch transition (the shell had a different orientation in images **F** and **G**). The arrow marks the constriction between protoconch and teleoconch that is a diagnostic character for this species. Scale bars: 0.5 mm (**A–E**); 0.2 mm (**F, G**).

This slender eulimid is characterized by a constriction at the transition between the protoconch and the teleoconch (Figure 15A, B, F). This feature distinguishes it at once from any native Mediterranean species. The protoconch is ~ 2.5 whorls, apparently without any ornamentation. The teleoconch is very slender, made of 6 translucent-white whorls, with flat sides, inconspicuous suture; the lip profile is arched. These characters fit the genus *Hypermastus* Pilsbry, 1899 to which we tentatively assign the species (Warén 1991b). We were not able to assign it to any Indo-Pacific species, but the family is among the most diverse and least known in that province (Bouchet et al. 2002), thus it could be another still undescribed species recently introduced into the Mediterranean Sea.

##### Hemiliostraca clandestina

Taxon classificationAnimaliaLittorinimorphaEulimidae

(Mifsud & Ovalis, 2019) comb. nov.

8E0158B8-1B2C-5F1D-AEB0-35B7094E0360

Figure 16A–C

###### New records.

Israel • 1 spcm; Ashqelon; 31.6868°N, 34.5516°E; depth 12 m; 30 Apr. 2018; offshore rocky reef; suction sampler; HELM project (sample S12_1F) • 38 spcms; Ashqelon; 31.6891°N, 34.5257°E; depth 25 m; 2 May 2018; offshore rocky reef; suction sampler; HELM project (samples S16_1F, S16_2F, S16_2M); size: H 2.7 mm, L 0.9 mm (illustrated shell) • 16 spcms; same collecting data as for preceding; depth 28 m; 31 Oct. 2018; HELM project (samples S59_1F, S59_2F, S59_3F) • 1 spcm; west of Rosh HaNikra Islands; 33.0725°N, 35.0923°E; depth 20 m; 1 May 2018; rocky substrate; suction sampler; HELM project (sample S13_3F).

###### Remarks.

*Sticteulima clandestina* and *S. athenamariae*, both Mifsud & Ovalis, 2019, were described on specimens collected in Turkey (Mifsud and Ovalis 2019). However, both belong to species present in the Red Sea and were illustrated by Blatterer (2019) for the Gulf of Aqaba on plate 131, fig. 8a–d and plate 131, fig. 9a–h, respectively. *Sticteulima clandestina* appears rather variable but our specimens clearly match Mifsud and Ovalis (2019: fig. 1B). Both *S. clandestina* and *S. athenamariae* look closely related to *Hemiliostraca* and thus we propose the new combinations *Hemiliostraca clandestina* and *Hemiliostraca athenamariae*. This is the first record of *H. clandestina* in Israel, but the species has been recorded for Lebanon based on empty shells collected in 1999 (Crocetta et al. 2020). Consequently, it is likely present here since at least 1999, with a ~ 20 year time-lag in first detection as quantified also for other non-indigenous species in the Mediterranean Sea (Crooks 2005; Albano et al. 2018). This is also the first record of living individuals from the Mediterranean Sea. Despite the relatively large number of living individuals, we did not find any attached to an echinoderm host; this is consistent with the fact that some eulimids actively leave the host if disturbed (Warén 1984).

**Figure 16.**
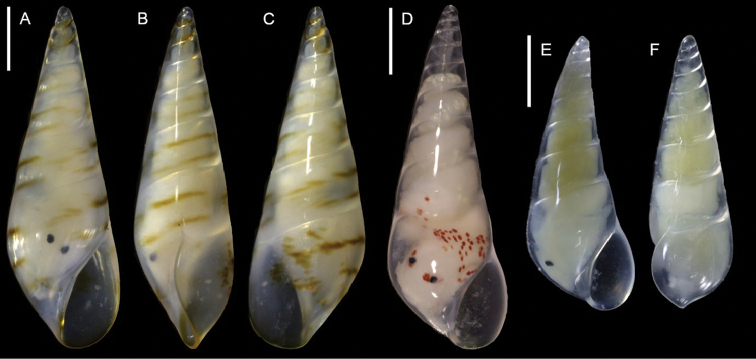
*Hemiliostraca clandestina* (Mifsud & Ovalis, 2019), and comparison between *Vitreolina philippi* (de Rayneval & Ponzi, 1854) and *Vitreolina* sp. **A–C***Hemiliostraca clandestina*, Ashqelon, Israel, HELM project (sample S16_1F): front (**A**), side (**B**) and back (**C**) views. **D***Vitreolina philippi* (de Rayneval & Ponzi, 1854), Plakias, Crete, Greece (sample Rh.05_5M): front view. **E, F**Vitreolina cf. philippi, Ashqelon, Israel, HELM project (S16_2F): front (**E**) and side (**F**) views. Scale bars: 0.5 mm.

##### Melanella orientalis

Taxon classificationAnimaliaLittorinimorphaEulimidae

Agamennone, Micali & Siragusa, 2020

1935B742-2122-522A-AFD3-6103311ADFC7

Figure 17

###### New records.

Israel • 5 spcms; Ashqelon; 31.6868°N, 34.5516°E; depth 12 m; 30 Apr. 2018; offshore rocky reef; suction sampler; HELM project (samples S12_1F, S12_1M, S12_3F); size: H 2.7 mm, W 1.0 (illustrated specimen) • 1 spcm; same collecting data as for preceding; depth 11 m; 31 Oct. 2018; HELM project (sample S58_2F) • 3 spcms; Ashqelon; 31.6891°N, 34.5257°E; depth 25 m; 2 May 2018; offshore rocky reef; suction sampler; HELM project (samples S16_1F, S16_2F) • 1 spcm; same collecting data as for preceding; depth 28 m; 31 Oct. 2018; HELM project (sample S59_1F).

###### Remarks.

This species can be distinguished from Mediterranean *Melanella* by its gently curved whorls, straight spire with fewer whorls and thinner shell than most species. It superficially resembles the Red Sea “*Eulima*” *orthophyes* Sturany, 1903 (type illustrated by Albano et al. (2017)), which can be distinguished because of its slightly bent apical whorls and the unusual pustulous sculpture of the protoconch. The species presented here is apparently already widespread in the Eastern Mediterranean (Agamennone et al. 2020a). We found only living individuals and no empty shells. Because of this, and the low likelihood that a so widespread species in shallow depths in the Eastern Mediterranean would have escaped detection for long, we consider it a new non-indigenous species in the basin. The specimens reported as *Melanella* sp. by Albano et al. (2020) from mesophotic reefs off northern Israel belong to this species.

**Figure 17.**
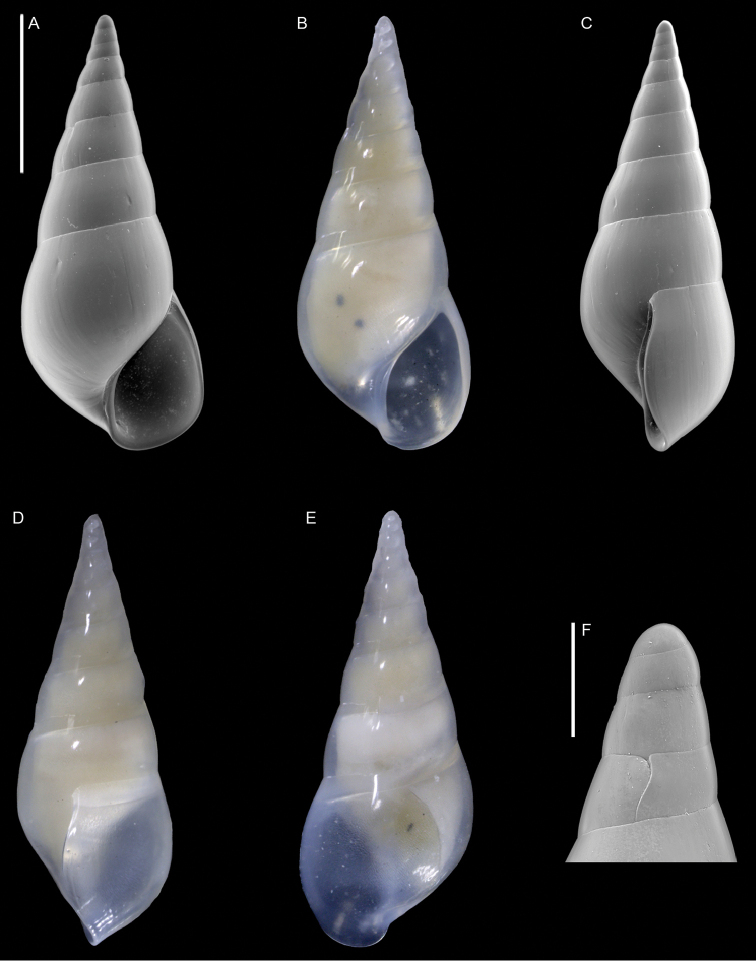
*Melanella orientalis* Agamennone, Micali & Siragusa, 2020, Ashqelon, Israel, HELM project (sample S12_1M): front (**A, B**), side (**C, D**) and back (**E**) views, apex (**F**). Scale bars: 1 mm (**A–E**); 0.2 mm (**F**).

##### Parvioris aff. dilecta

Taxon classificationAnimaliaLittorinimorphaEulimidae

(E.A. Smith, 1899)

5E491ADA-04EB-52FB-893A-01858CD347C2

Figure 18E–M

###### New records.

**Figure 18.**
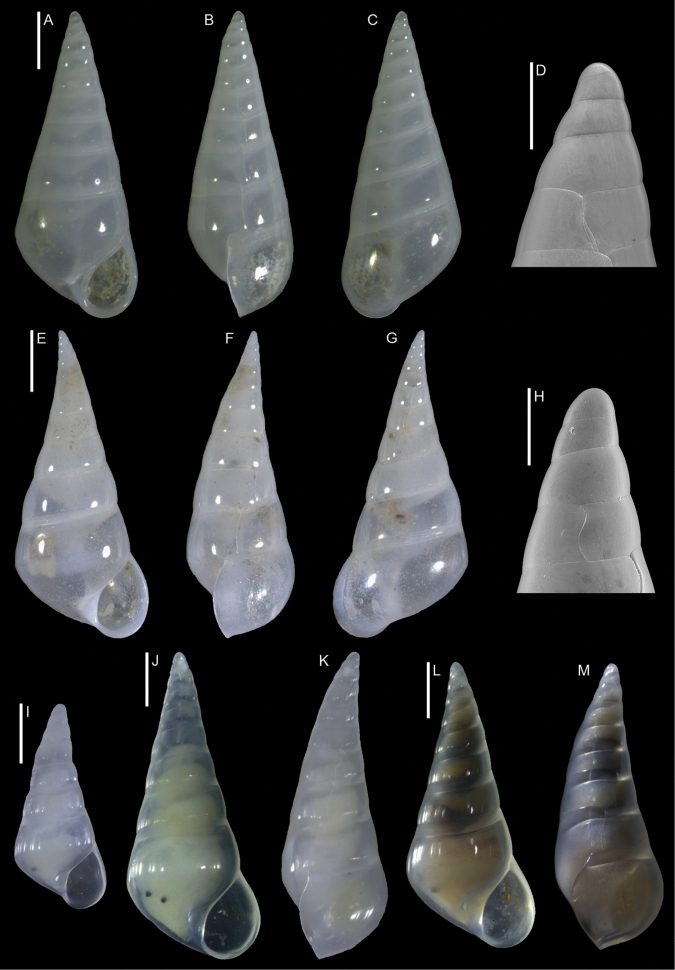
Comparison between Parvioris aff. dilecta (E.A. Smith, 1899) and *Parvioris ibizenca* (F. Nordsieck, 1968) **A–D***Parvioris ibizenca*, west of Rosh HaNikra Islands, Israel, HELM project (sample S52_2F): front (**A**), side (**B**) and back (**C**) views, protoconch (**D**) **E–H**Parvioris aff. dilecta, north of Atlit, Israel, HELM project (sample NG40_2M): front (**E**), side (**F**) and back (**G**) views, protoconch (**H**) **I**Parvioris aff. dilecta, west of Rosh HaNikra Islands, Israel, HELM project (sample S52_1F): front view **J–K**Parvioris aff. dilecta, west of Rosh HaNikra Islands, Israel, HELM project (sample S52_1M): front (**J**) and side (**K**) views **L, M**Parvioris aff. dilecta, same collecting data as for preceding, HELM project (sample S52_2F): front (**L**) and side (**M**) views. Scale bars: 0.5 mm (**A–C, I–M**); 0.2 mm (**D, H**); 1 mm (**E–G**).

Israel • 1 sh; north of Atlit; 32.7433°N, 34.9067°E; depth 40 m; 20 Sep. 2016; coarse biogenic sediment in a pool among rocks covered by coralligenous formations; grab; HELM project (sample NG40_2M); size: H 4.9 mm, W 2.2 mm (illustrated shell, Figure 18E–H) • 3 spcms; west of Rosh HaNikra Islands; 33.0704°N, 35.0926°E; depth 12 m; 29 Oct. 2018; rocky substrate; suction sampler; HELM project (samples S52_1F, S52_1M, S52_2F).

###### Additional material examined.

*Parvioris ibizenca* (F. Nordsieck, 1968): Israel • 1 sh; west of Rosh HaNikra Islands; 33.0704°N, 35.0926°E; depth 12 m; 29 Oct. 2018; rocky substrate; suction sampler; HELM project (sample S52_2F).

###### Remarks.

The genus *Parvioris* Warén, 1981 was erected for a group of numerous conchologically very similar species of which many are still undescribed (Warén 1981). The species here reported is very similar in general shape and size to *P. dilecta* (Warén 1981: 146), especially the morphs illustrated here in Figure 18I–M. However, it has a multispiral protoconch of ~ 4.5 whorls (Figure 18H), whereas *P. dilecta* has a paucispiral protoconch of ~ 1.5 whorls. The type of protoconch is considered to be related to the developmental mode, which was regarded a diagnostic character at the species level for most molluscan lineages (Hoagland and Robertson 1988; Bouchet 1989). Because Warén (1984) suggested that the number of protoconch whorls is rather constant within species in Eulimidae, we currently do not consider our material conspecific with *P. dilecta*, but only closely related (thus the “aff.” notation). However, there is increasing evidence that poecilogony, the intraspecific variation in developmental mode, occurs in Caenogastropoda (McDonald et al. 2014), Neogastropoda (Russini et al. 2020), and Sacoglossa (Krug 1998; Ellingson and Krug 2006; Vendetti et al. 2012).

Parvioris aff. dilecta can be easily distinguished from the native *P. ibizenca* because of a more arched apical part and because of the protoconch morphology: both have multispiral protoconchs, but *P. ibizenca* has shorter whorls and a distinct profile which inflates at the third whorl, in contrast with the more slender and regular profile of P. aff. dilecta. Our specimens are likely conspecific with those identified as *Melanella* sp. 1 by Blatterer (2019) from Dahab, Red Sea, suggesting that it is indeed a new Lessepsian species. An additional issue is whether the animal color is diagnostic at the species level like in other groups whose shells offer few diagnostic morphological characters, e.g., Mediterranean *Granulina* (Neogastropoda: Granulinidae) and *Gibberula* (Neogastropoda: Cystiscidae) (Gofas 1990, 1992). Some of our live collected specimens show a light yellow-white color (e.g., Figure 18I–K) whereas others have a brownish animal (e.g., Figure 18K–M). The final attribution of our findings to a species requires a thorough revision of *Parvioris*, which is beyond the scope of this paper. The specimens reported as *Parvioris* sp. by Albano et al. (2020) from mesophotic reefs off northern Israel belong to this species.

##### Sticteulima

Taxon classificationAnimaliaLittorinimorphaEulimidae

sp.

56E6BF5E-B91C-54A6-A07E-BFCF70109915

Figure 19

###### New records.

**Figure 19.**
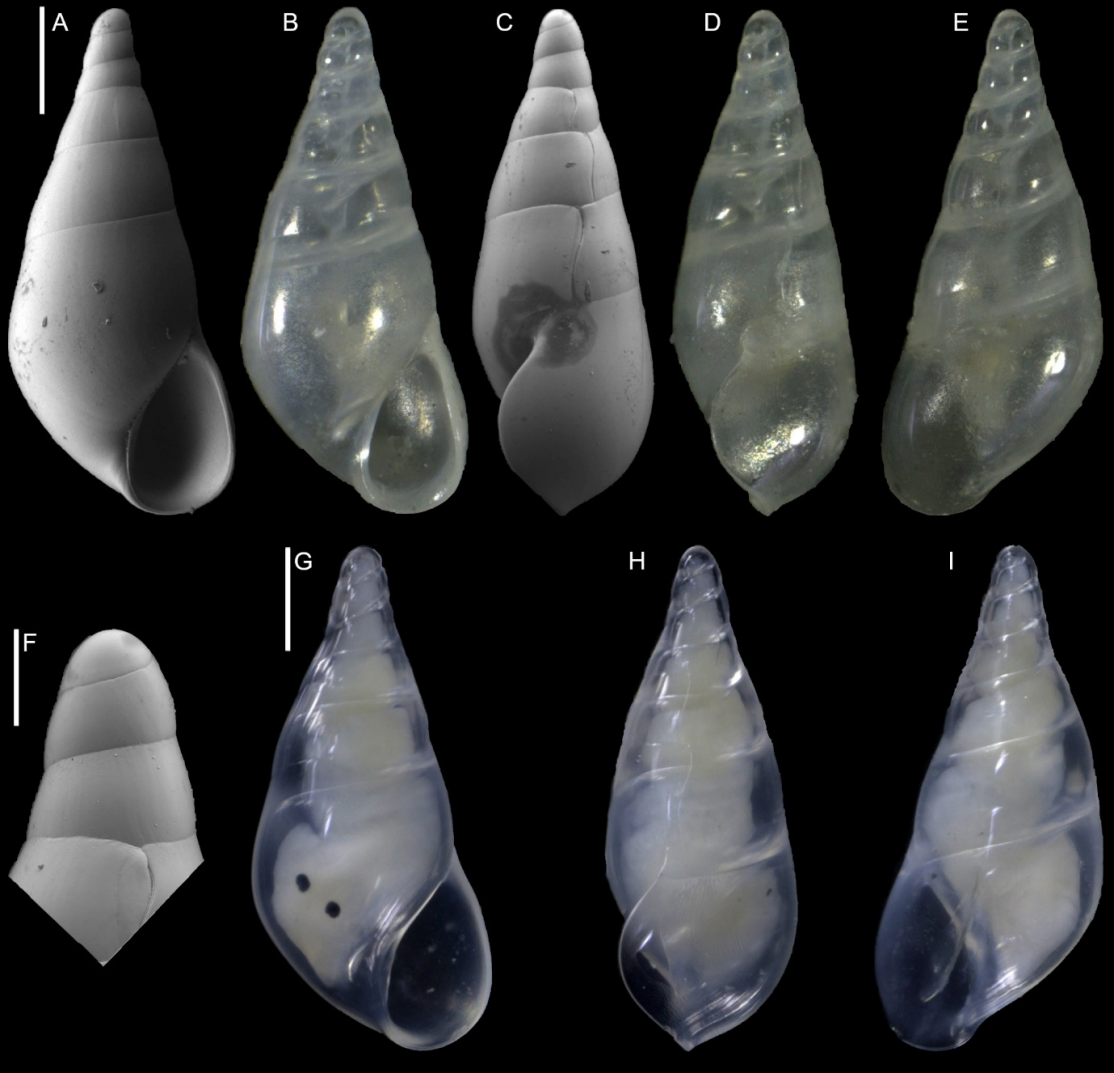
*Sticteulima* sp. **A–F** north of Atlit, Israel, HELM project (sample NG10_1F): front (**A, B**), side (**C, D**) and back (**E**) views, protoconch (**F**) **G–I** Ashqelon, Israel, HELM project (sample S59_1F): front (**G**), side (**H**) and back (**I**) views. Scale bars: 0.3 mm (**A–E, G–I**); 0.1 mm (**F**).

Israel • 1 sh; north of Atlit; 32.7820°N, 34.9466°E; depth 10 m; 21 Sep. 2016; sand; grab; HELM project (sample NG10_1F); size: H 1.4 mm, W 0.6 mm (illustrated shell, Figure 19A–F) • 1 spcm; Ashqelon; 31.6891°N, 34.5257°E; depth 28 m; 31 Oct. 2018; offshore rocky reef; suction sampler; HELM project (sample S59_1F).

###### Remarks.

We place this species in *Sticteulima* due to its small size, slender profile with high and rather flat whorls (Warén 1984). In contrast to the native *S. jeffreysiana* (Brusina, 1869) and the Lessepsian *S. lentiginosa* (A. Adams, 1861), it is colorless, also in live-collected specimens, and stouter. Further, this species does not match any of the known small-sized Mediterranean eulimids. It can be readily distinguished from *Vitreolina curva* (Monterosato, 1874) and *Melanella levantina* (Oliverio, Buzzurro & Villa, 1994) by the lack of the strongly arched apical whorls. This feature differentiates it at once also form other Red Sea small-sized eulimids (Blatterer 2019). *Melanella petitiana* (Brusina, 1869) is larger, has more numerous whorls (our *Sticteulima* has a fully thickened lip suggesting that it is an adult) and has a less prominent lip profile. *Nanobalcis nana* (Monterosato, 1878) (type illustrated by Appolloni et al. (2018)) has shorter whorls, especially the last one, which is also much broader than in this species. It can also be easily distinguished from *Hemiliostraca athenamariae* (Mifsud & Ovalis, 2019) by the lack of any color pattern, and the more inflated lip profile with a deeper posterior sinus. *Sticteulima* sp. may be a new non-indigenous species in the Mediterranean Sea.

##### Vitreolina cf. philippi

Taxon classificationAnimaliaLittorinimorphaEulimidae

(de Rayneval & Ponzi, 1854)

A11FDE76-3446-541F-AD35-3A91F83E6BA4

Figure 16E, F

###### New records.

Israel • 4 spcms; Ashqelon; 31.6891°N, 34.5257°E; depth 25 m; 2 May 2018; offshore rocky reef; suction sampler; HELM project (samples S16_1F, S16_2F); size: H 2.6 mm, W 0.9 mm.

###### Additional material examined.

*Vitreolina philippi* (de Rayneval & Ponzi, 1854): GREECE • Crete, Plakias; 35.1796°N, 24.3957°E; depth 5 m; 24 Sep. 2017; *Posidonia oceanica* rhizomes; suction sampler (sample Rh.05_5M).

###### Remarks.

This *Vitreolina* is extremely similar to the native *V. philippi*, but the animal is whitish with a yellowish digestive gland (Figure 16E, F), in contrast to the peculiar color pattern of typical *V. philippi* with a white background and red dots (Figure 16D). *Vitreolina* is known to be gonochorous (Warén 1984) but it is unclear if this different color pattern, never reported from the Mediterranean, can be related to sex. We suspect that this could be another new Lessepsian species for the Mediterranean Sea, because we observed several Mediterranean-Red Sea species pairs that are morphologically extremely similar. If we are correct, the occurrence and distribution of this species in the Mediterranean may be difficult to trace, because empty shells, the most easily collected, are virtually indistinguishable from the native *V. philippi*.

#### Family Conidae J. Fleming, 1822

##### Conus fumigatus

Taxon classificationAnimaliaNeogastropodaConidae

Hwass in Bruguière, 1792

A5F375E2-F37F-51F7-82F9-40748D927259

Figure 20

###### New records.

**Figure 20.**
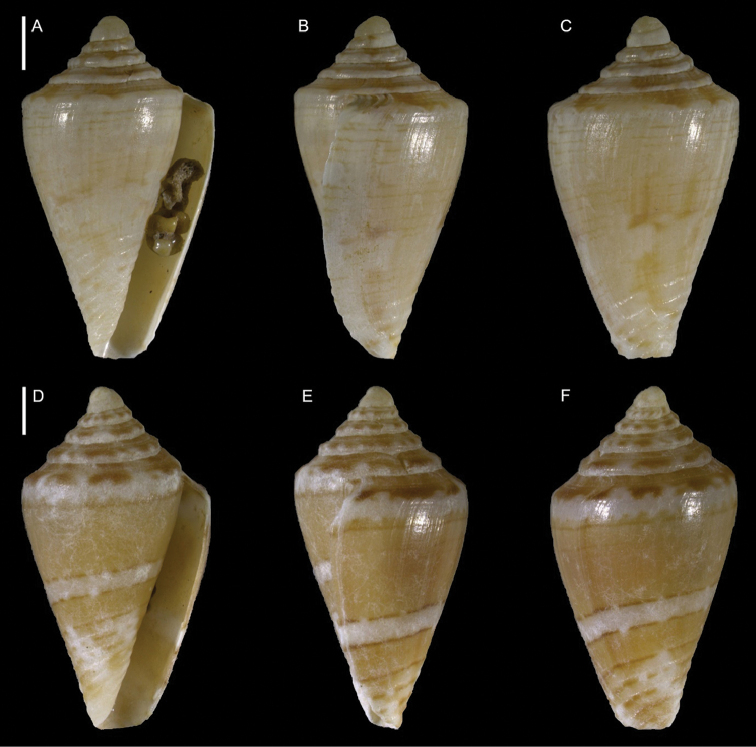
*Conus fumigatus* Hwass in Bruguière, 1792, NHMW-MO-112930/LM/0175, north of Atlit, Israel, HELM project (sample NG40_2M) **A–C** Shell 1: front (**A**), side (**B**) and back (**C**) views **D–F** Shell 2: front (**D**), side (**E**) and back (**F**) views. Scale bars: 1 mm.

Israel • 3 shs; north of Atlit; 32.7433°N, 34.9067°E; depth 40 m; 20 Sep. 2016; coarse biogenic sediment in a pool among rocks covered by coralligenous formations; grab; HELM project (sample NG40_2M); NHMW-MO-112930/LM/0175; size of the largest shell (illustrated shell 2, Figure 20D–F): H 7.2 mm, W 4.0 mm.

###### Remarks.

*Conus fumigatus* was first recorded from the Mediterranean Sea in Libya (Röckel 1986) but not recorded again for three decades until a recent report from Syria (Ammar 2018). This is the first finding in Israel, filling the distributional gap from the Suez Canal northward; only shells of juveniles have been found so far.

#### Family Murchisonellidae T.L. Casey, 1904

##### Henrya

Taxon classificationAnimaliaMurchisonellidae

(?) sp.

ADE46B1C-F6E1-59B5-B45A-36665BE5CB6D

Figure 21

###### New records.

Israel • 1 spcm; Palmachim; 31.9574°N, 34.6645°E; depth 36.2 m; 24 May 2017; soft substrate; box-corer; Shafdan project (sample 24(B)); size: H 1.6 mm, W 0.8 mm.

###### Remarks.

We were unable to assign this species to any Mediterranean or Indo-Pacific species, despite its conspicuous combination of shell characters. Our single specimen has an elongated, pupoid shell with convex whorls, a narrow but deeply incised suture, and a heterostrophic protoconch of type B (diameter: 250 µm). The surface is glossy and smooth except for densely spaced, very fine growth lines. The latter are straight, slightly prosocline on the spire, becoming orthocline near the aperture. The aperture is drop-shaped with a simple, thin lip that is slightly reflected at the columella. An umbilical chink is present. The shell is translucid-white, ornamented with a single, broad, light brown spiral color band. The shell morphology is similar to species of the murchisonellid genus *Henrya* Bartsch, 1947. However, the three currently known species of that genus were described form the tropical West Atlantic (Florida, Bahamas, and Yucatan) (Bartsch 1947), and none of them has a brown color band. For these reasons, the lack of anatomical and molecular data, and the fact that only a single specimen was available for study, we refrained from a definitive generic assignment. This species is potentially another non-indigenous one originating from the Indo-Pacific.

Among Mediterranean gastropods, the shell shape somewhat resembles the iravadiid *Hyala vitrea* (Montagu, 1803), however, the semi-immersed protoconch and brown color band of *Henrya* (?) sp. immediately set it apart. The heterobranch *Cima minima* (Jeffreys, 1858) is smaller, has a more concial shape, flexuous growth lines, and also lacks the brown band (van Aartsen 1981; Gofas et al. 2011; Scaperrotta et al. 2012; Giannuzzi-Savelli et al. 2014).

**Figure 21.**
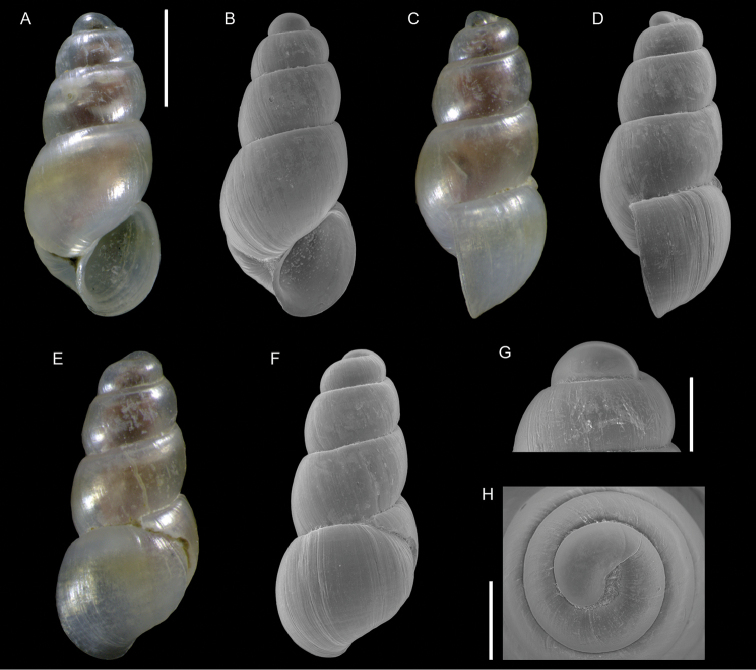
*Henrya* (?) sp., Palmachim, Israel, Shafdan project (sample 24(B)): front (**A, B**), side (**C, D**) and back (**E, F**) views, apex (**G**) and apical view of the protoconch (**H**). Scale bars: 0.5 mm (**A–F**); 0.2 mm (**G, H**).

#### Family Pyramidellidae Gray, 1840

##### Odostomia cf. dalli

Taxon classificationAnimaliaPyramidellidae

(Hornung & Mermod, 1925)

1388FE88-4223-5F68-AFB2-193E50B6C700

Figure 22

###### New records.

Israel • 4 spcms; west of Rosh HaNikra Islands; 33.0704°N, 35.0926°E; depth 12 m; 29 Oct. 2018; rocky substrate; suction sampler; HELM project (samples S52_1F, S52_2F); size: H 2.1 mm, W 1.0 mm (illustrated specimen) • 1 spcm; west of Rosh HaNikra Islands; 33.0725°N, 35.0923°E; depth 19 m; 29 Oct. 2018; rocky substrate; suction sampler; HELM project (sample S53_1F).

###### Remarks.

**Figure 22.**
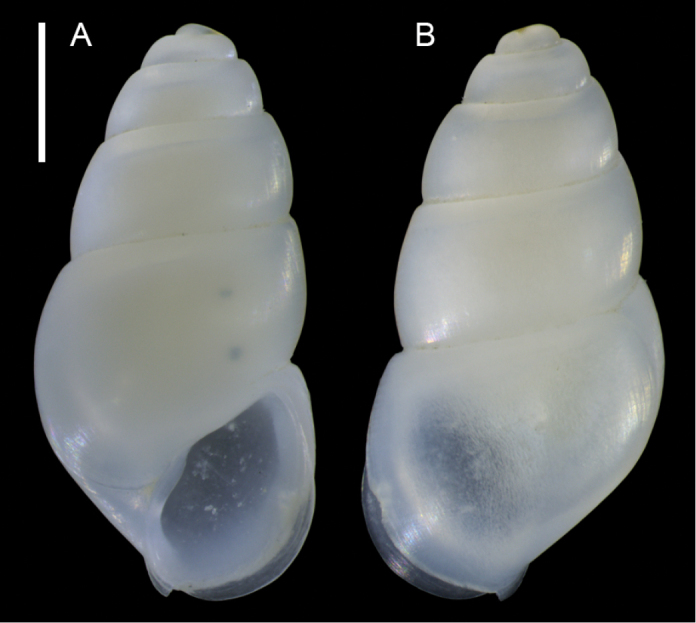
Odostomia cf. dalli (Hornung & Mermod, 1925), west of Rosh HaNikra Islands, Israel, HELM project (sample S52_2F): front (**A**) and back (**B**) views. Scale bar: 0.5 mm.

The shell of this species is white and rather solid, with convex, unkeeled whorls and a deep, narrow suture. The columellar tooth is visible in frontal view; there are no lirae inside the aperture. The outer surface appears smooth at first sight but bears numerous very fine spiral lines. The protoconch is of type A2, tending to type B. In ethanol-preserved specimens, the soft body is yellowish-white, with the eyes well visible through the shell (Figure 22A). Odostomia cf. dalli differs in its shell morphology from all known Mediterranean Odostomiinae, but bears close resemblance to the illustration of the type specimen of *Odostomia dalli* from Sarad Island (“Ile de Sarato”), Dahlak Archipelago, Eritrean Red Sea (Hornung and Mermod 1925). In contrast to our material, however, *O. dalli* was described as lacking both, spiral sculpture and a visible columellar fold, although a columellar tooth seems to be indicated in the line drawing accompanying the original description. A rigorous assessment of potential conspecificity between *O. dalli* and our material therefore awaits a thorough study of the type material of the former, but the close similarity suggests that this is a new non-indigenous species in the Mediterranean Sea. This interpretation is also supported by the lack of empty shells in death assemblages (see Discussion).

##### Odostomia

Taxon classificationAnimaliaPyramidellidae

(s.l.) sp. 1

9E3D8E90-6BEC-5445-A6A5-9CCFAD5925EE

Figure 23

###### New records.

Israel • 1 spcm; Haifa Bay; 32.8211°N, 35.0196°E; depth 11 m; 2 Aug. 2015; soft substrate; grab; NM project (sample HM27(c)); size: H 1.4 mm, W 0.7 mm (illustrated specimen) • 8 spcms; 31.9364°N, 34.6846°E; depth 20.2 m; 11 Oct. 2012; sandy substrate; grab; Via Maris project (sample VM40).

###### Remarks.

**Figure 23.**
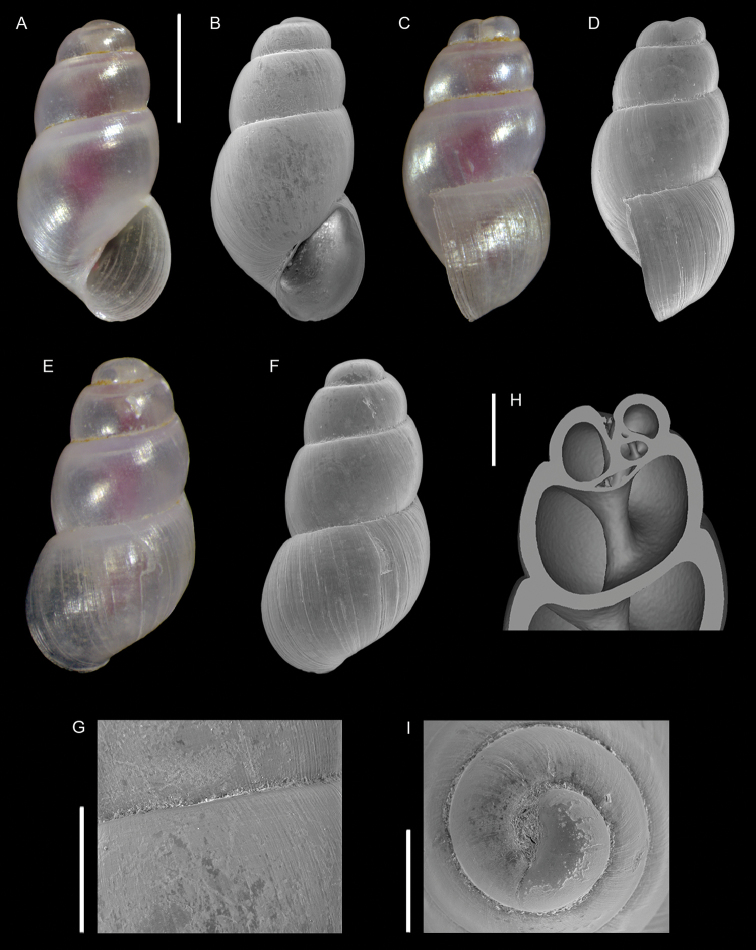
*Odostomia* (s.l.) sp. 1, Haifa Bay, Israel, NM project (sample HM27(c)): front (**A, B**), side (**C, D**) and back (**E, F**) views, detail of the adapical part of the body whorl showing the extremely faint spiral microsculpture (**G**), virtual section through the apical spire showing the columella of the intorted protoconch (**H**) and apical view of the protoconch (**I**; surface partly corroded). The pink hue is due to staining with eosin solution. Scale bars: 0.5 mm (**A–F**); 0.2 mm (**G–I**).

This species is characterized by a translucid-white, cylindrical shell with ~ 3 whorls, and an intorted protoconch of type C (Figure 23I) whose columella is oriented at an angle of ~ 160° relative to the teleoconch axis (revealed by µCT-imaging, Figure 23H and additional scans available at https://doi.org/10.6084/m9.figshare.c.5215226). The growth lines are slightly prosocline on the spire while becoming almost orthocline on the body whorl; an extremely faint spiral microsculpture is present on the apical part of the whorls, but only visible in high-magnification SEM images (Figure 23G). This species differs from Odostomia cf. dalli by its smaller size (height up to 1.4 mm), the more cylindrical shape, shallower suture, and the absence of a visible columellar tooth. Although this species, in terms of size and overall shape, somewhat resembles representatives of the fresh- and brackish water-dwelling family Hydrobiidae, the fact that numerous living specimens were found in a fully marine environment and its heterostrophic protoconch unambiguously identify it as member of the family Pyramidellidae.

*Odostomia* sp. 1 does not resemble any known Mediterranean pyramidellid; considering the great number of confirmed introductions of Indo-Pacific microgastropods to the eastern Mediterranean Sea, we therefore suspect that also this taxon might be a Lessepsian species. Among Indo-Pacific Odostomiinae, *O. bullula* Gould, 1861 (e.g., Johnson 1964; Robba et al. 2004) is similar to our specimens, but differs by its more conical shape and larger size (height to 2 mm, width to 1 mm). Another similar species, *O. decouxi* Saurin, 1959, was suggested to be a junior synonym of *O. bullula* (Robba et al. 2004).

##### Odostomia

Taxon classificationAnimaliaPyramidellidae

(s.l.) sp. 2

74254F6B-2E77-572F-9E90-FDC5EC4EDC89

Figure 24

###### New records.

Israel • 1 spcm; Soreq desalination plant; 31.9420°N, 34.6896°E; depth 17.4 m; 19 May 2015; soft substrate; grab; Soreq project (sample S027).

###### Additional material examined.

Israel • 1 sh; Neve Yam, Atlit; 32.6785°N, 34.9289°E; 6 Feb. 2006; beached; size H 1.3 mm, W 0.7 mm (illustrated shell; previously figured by Bogi and Galil (2006)).

###### Remarks.

**Figure 24.**
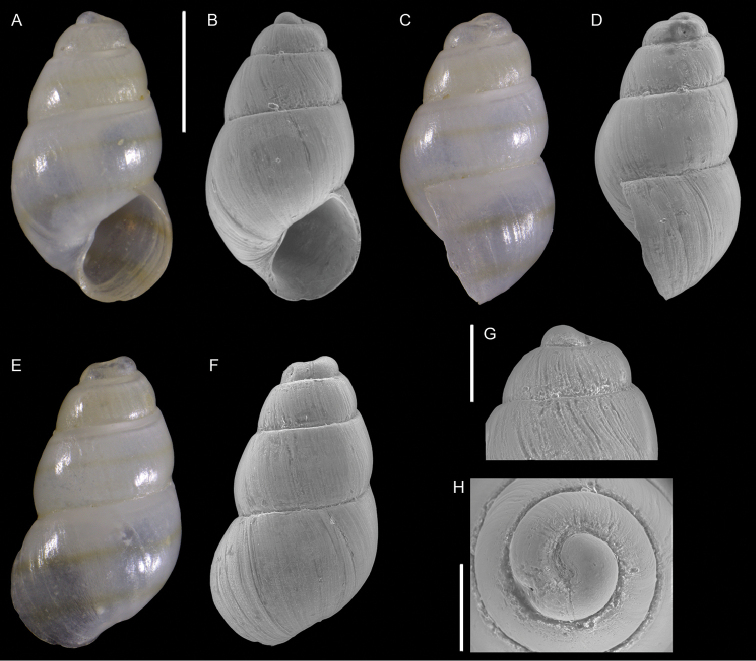
*Odostomia* (s.l.) sp. 2, Neve Yam, Atlit, Israel: front (**A, B**), side (**C, D**) and back (**E, F**) views, apex (**G**) and apical view of the protoconch (**H**). Scale bars: 0.5 mm (**A–F**); 0.2 mm (**G, H**).

The first record of this species is based on five well-preserved shells found in a shell grit sample taken in 1995 on the beach of Yumurtalik, Adana, Turkey (Giunchi et al. 2001). In 2006, another beached shell was found at Neve Yam, northern Israel (Bogi and Galil 2006, re-illustrated in Figure 24 herein) and, according to these authors, the species was also found in Israel by J.J. van Aartsen. A specimen of *Odostomia* sp. 2, from the original lot from Yumurtalik, was recently figured by Giannuzzi-Savelli et al. (2014). Here, we report the first finding of a living individual of *Odostomia* sp. 2 which was recovered from a sediment sample taken at the Soreq desalination plant, southern Israel.

Since the first finding in Turkey 25 years ago, the identity of this most likely non-indigenous species has remained unresolved. It differs from all known Mediterranean Odostomiinae at first glance by the presence of two brown spiral bands. We are unaware of any Indo-Pacific pyramidellid resembling this taxon, and it may well represent an undescribed species. To aid the further study of this taxon and raise awareness of its presence and apparent spread in the Mediterranean, we here re-illustrate the well-preserved shell from Neve Yam using light and scanning electron microscopy.

##### Oscilla virginiae

Taxon classificationAnimaliaPyramidellidae

Peñas, Rolán & Sabelli, 2020

0FAF17DC-6FDD-5EFA-B330-4E81DE1C1513

Figure 25A, B

###### New records.

Israel • 1 spcm; west of Rosh HaNikra Islands; 33.0704°N, 35.0926°E; depth 12 m; 29 Oct. 2018; rocky substrate; suction sampler; HELM project (sample S52_1F) • 1 spcm; west of Rosh HaNikra Islands; 33.0725°N, 35.0923°E; depth 19 m; 29 Oct. 2018; rocky substrate; suction sampler; HELM project (sample S53_1F); size: H 1.6 mm, W 0.9 mm (illustrated specimen).

###### Additional material examined.

*Oscilla appeliusi* (Hornung & Mermod, 1925): EGYPT • 1 sh; Sinai (Red Sea), south of Sharm-el-Sheik, Na’ama Bay; 27.8500°N, 34.2833°E; depth 0–6 m; 28 Sep. –4 Oct. 1978; rocky substrate; A.J. Ferreira leg.; LACM 1978-79.4 • 1 sh; Sinai (Red Sea), Gulf of Aqaba, Strait of Tiran, Jackson Reef ; 28.0167°N, 34.4667°E; depth 2–3 m; 31 Oct. 1985; sand and coral substrate; T. Bratcher leg.; LACM 1985-111.5 • 2 shs; Sinai (Red Sea), Gulf of Aqaba, Strait of Tiran, east side Jackson Reef; 28.000°N, 34.4667°E; depth 10 m; 9 Jul. 1988; coral rubble; J. H. Golden leg.; LACM 1988-118.1 • 4 shs; Sinai (Red Sea), off Ras Umm Sid, “Amphoras” dive site; 27.8667°N, 34.333°E; depth 18 m; 24 Jul. 1988; coral rubble; J.H. Golden leg.; LACM 1988-119.2.

Indonesia • 7 shs; Papua Province, south-east of Biak Island, east side of Auki Islet; 1.2300° S, 136.3367°E; depth 0 m; 5 Apr. 1988; rock; J.H. McLean & E. Abbott leg.; LACM 1988-48.12.

Taiwan • 7 shs; Tai-Pei County, east of Chi-lung (= Keelung), south-east side of Pitou Chiao (= Pitou Nonkow); 25.1333°N, 121.9167°E; depth 0–3 m; 10 May 1988; rocky tide pool; C.C. Coney & P.F. Liu leg.; LACM 1988-80.13.

*Miralda* sp. (*Oscilla jocosa* sensu van Aartsen et al. (1989)): Israel • 1 spcm; west of Rosh HaNikra Islands; 33.0704°N, 35.0926°E; depth 12 m; 29 Oct. 2018; rocky substrate; suction sampler; HELM project (sample S52_1F); size: H 2.0 mm, W 1.0 mm (illustrated specimen).

###### Remarks.

**Figure 25.**
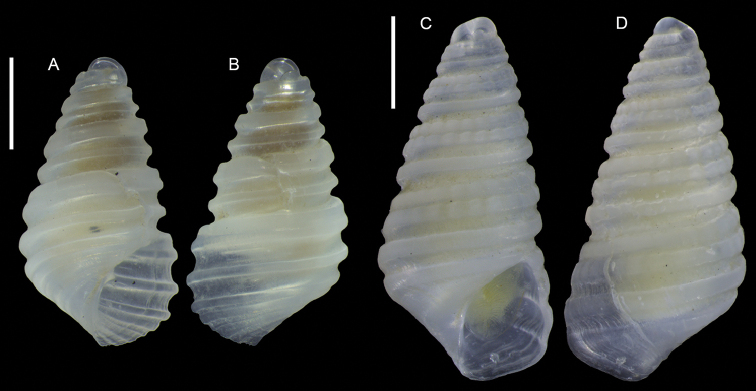
Comparison between *Oscilla virginiae* Peñas, Rolán & Sabelli, 2020 and *Miralda* sp. **A, B***Oscilla virginiae*, west of Rosh HaNikra Islands, Israel, HELM project (sample S53_1F): front (**A**) and back (**B**) views **C, D***Miralda* sp. (*Oscilla jocosa* sensu van Aartsen et al. (1989)), west of Rosh HaNikra Islands, Israel, HELM project (sample S52_1F): front (**C**) and back (**D**) views. Scale bars: 0.5 mm.

*Oscilla virginiae* is characterized by a small-sized, white, conical shell with a type A protoconch. The sculpture consists of thick, smooth spiral cords: the first and second whorl bear two cords; the upper cord is broadest and bifurcates on the third whorl, forming three cords on the last whorl, with the newly formed pair remaining positioned very close one to each other (Figure 25A, B; Peñas et al., 2020).

This species has just been described from the infralittoral of Jordan and also occurs in the Egyptian Red Sea (Peñas et al. 2020). It superficially resembles the Indo-Pacific *O. appeliusi* (Hornung & Mermod, 1925), and indeed, a juvenile shell from Dahab (Egypt) was recently figured by Blatterer (2019: plate 212, fig. 17c, d) under this name. In contrast to *O. virginiae*, however, *O. appeliusi* bears spiral cords more similar in thickness which are spaced more equidistantly and closer to each other. Already on the second whorl, three cords are present, and the uppermost cord does not evidently bifurcate (Peñas et al. 2020). Lastly, the illustration of Hornung and Mermod (1925) suggests a greater number of spiral cords on the last whorl. To date, *O. appeliusi* has not been recorded from the Mediterranean Sea.

Within the Mediterranean, *O. virginiae* is superficially similar only to two other non-indigenous pyramidellids, *Cingulina isseli* (Tryon, 1886) and *Miralda* sp. (Figure 25C, D). The latter taxon has previously been reported under the name *Oscilla jocosa* Melvill, 1904, despite recent evidence by Peñas & Rolán (2017) that it is not conspecific with Melvill’s (1904) type material. *Oscilla virginiae* differs from *C. isseli* by its broader, more conical shell, fewer whorls, smaller size (*C. isseli* reaches a height of ~ 3 mm), and the much less pronounced axial sculpture between the spiral cords. Compared to *Miralda* sp., *O. virginiae* differs by its smaller size (up to ~ 3 mm in *Miralda* sp.), the absence of beads on the two upper spiral cords (Figure 25C, D), and stronger spiral cords on the base of the shell. Another *Oscilla* present in the Mediterranean Sea is *O. galilae* Bogi, Kharan & Yokes, 2012, which can be easily distinguished from *O. virginiae* by the smaller size, the oval profile, the oblique spiral sculpture, the more prominent axial sculpture and a type C protoconch. Peñas et al. (2020) illustrated *O. galilae* (their Fig. 38A–E) under the name *O. cylindrica* (de Folin, 1879) suggesting synonymy between the two names. However, *O. cylindrica* can be easily distinguished from *O. galilae* because it bears spiral cords without interspaces, because it does not bear the fine axial lamellae typical of *O. galilae* and because of its more cylindrical profile. The Red Sea records of *O. virginiae* and *O. galilae* by Peñas et al. enable to assess that both species are non-indigenous in the Mediterranean Sea.

##### Parthenina cossmanni

Taxon classificationAnimaliaPyramidellidae

(Hornung & Mermod, 1924)

57CBF6DB-D2E5-53CA-A47B-1D904A43EEAA

Figure 26A–H

###### New records.

**Figure 26.**
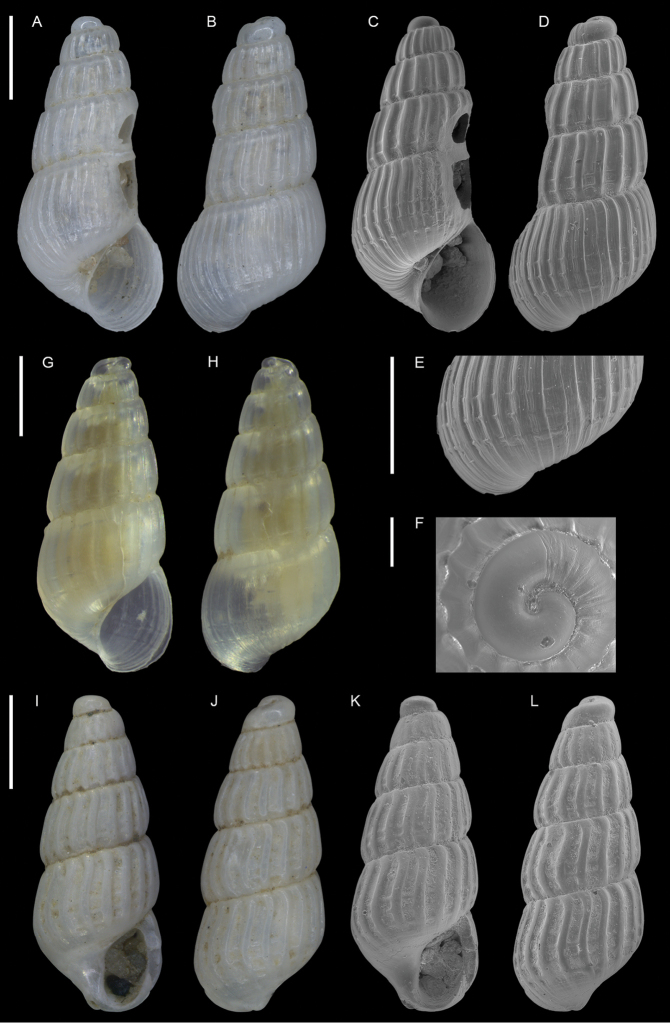
Comparison between *Parthenina cossmanni* (Hornung & Mermod, 1924) and *Parthenina indistincta* (Montagu, 1808) **A–F***Parthenina cossmanni*, north of Atlit, Israel, HELM project (sample NG30_2F): front (**A, C**) and back (**B, D**) views, detail of the spiral sculpture on the body whorl (**E**) and apical view of the protoconch (**F**) **G, H***Parthenina cossmanni*, Ashqelon, Israel, HELM project (sample SG20_4F): front (**G**) and back (**H**) views **I–L***Parthenina indistincta*, Ashqelon, Israel, HELM project (sample SC30_1_15L): front (**I, K**) and back (**J, L**) views. Scale bars: 0.5 mm (**A–E, G–L**); 0.1 mm (**F**).

Israel • 1 sh; north of Atlit; 32.7422°N, 34.9181°E; depth 30 m; 20 Sep. 2016; sand; grab; HELM project (sample NG30_2F); size: H 1.9 mm, W 0.9 mm (illustrated shell, Figure 26A–F) • 9 spcms, 2 shs; Ashqelon; 31.7002°N, 34.5498°E; depth 21 m; 18 Sep. 2016; sand; grab; HELM project (samples SG20_1F, SG20_2F, SG20_4F, SG20_5F); size: H 2.0 mm, W 0.8 mm (illustrated specimen, Figure 26G, H) • 1 spcm; Ashqelon; 31.6868°N, 34.5516°E; depth 12 m; 30 Apr. 2018; offshore rocky reef; suction sampler; HELM project (sample S12_1F).

###### Additional material examined.

*Parthenina indistincta* (Montagu, 1808): Israel • 2 shs; Ashqelon; 31.7002°N, 34.5498°E; depth 21 m; 18 Sep. 2016; sand; grab; HELM project (sample SG20_2F) • 1 sh; Ashqelon; 31.7101°N, 34.5406°E; depth 31 m; silty sand, 15 cm below sediment surface; gravity corer; HELM project (sample SC30_1_15L); size: H 1.7 mm, W 0.7 mm (illustrated shell).

###### Remarks.

*Parthenina cossmanni* has an elongated-conical shell with flat-convex whorls and a protoconch of type C. The whorls of the spire have a subangular profile, whereas the body whorl in adult specimens is more convex and evenly rounded. The axial sculpture is made of strong orthocline ribs that become slightly flexuous on the body whorl in some specimens (Figure 26A–E). The spiral sculpture on the spire consists of a single, thin, suprasutural cord; a second cord emerges on the penultimate whorl, and three cords are present on the last whorl. The columellar tooth is weak and deeply inset, and in some of the studied specimens hardly visible inside the aperture. The soft body of ethanol-preserved specimens is yellowish, with the eyes visible through the shell (Figure 26G, H).

The type material of *P. cossmanni* was collected from the Red Sea of Massawa (Eritrea) at a depth of 30 m (Hornung and Mermod 1924); the species was recently recorded from Dahab (Gulf of Aqaba, northern Egypt) by Blatterer (2019), and from Jordan by Peñas et al. (2020). Outside the Red Sea, it is known from Vietnam (Saurin 1959) and Thailand (Robba et al. 2004).

Among native Mediterranean species, *P. cossmanni* superficially resembles *Parthenina interstincta* (J. Adams, 1797). The latter species, however, has only two spiral cords on the last whorl and a more developed columellar tooth. *P. cossmanni* is further similar to *P. indistincta* (Montagu 1808) (Figure 26I–L) which has a very weak, internal columellar fold (Warén 1991a: 96, fig. 29f) and three (rarely four) spiral cords on the last whorl. Compared to *P. cossmanni*, however, the shell of *P. indistincta* is more elongated and has two spiral cords on the spire whorls.

We suspect the two shells illustrated as *P. indistincta* in Öztürk et al. (2011: fig. 10A, B) might also be *P. cossmanni*, considering their broad shape and overall morphology. Öztürk et al.’s material was collected in 2009 from a mud bottom at 9 m depth in Mersin Bay (stn. 46, 36.7167°N, 34.8667°E), south-eastern Turkey; should our hypothesis be confirmed upon re-examination of these shells, this would suggest that *P. cossmanni* likely has a wider distribution in the southeastern Mediterranean Sea. Our finding of several living specimens on the Israeli shelf, together with the relative rarity of empty shells in the samples, suggests that this species might have established locally only rather recently.

##### Parthenina typica

Taxon classificationAnimaliaPyramidellidae

(Laseron, 1959)

7818AA37-19B0-53FE-B12F-0133F80D8969

Figure 27

###### New records.

Israel • 2 spcms; Palmachim; 31.9737°N, 34.6767°E; depth 35.4 m; 2 Sep. 2015; soft substrate; box-corer; Shafdan project (sample 29(C)) • 4 spcms; Palmachim; 31.9685°N, 34.6732°E; depth 35.6 m; 2 Sep. 2015; soft substrate; box-corer; Shafdan project (sample 26(B)); size: H 2.0 mm, W 0.8 mm (illustrated specimen) • 1 sh; north of Atlit; 32.7433°N, 34.9067°E; depth 40 m; 20 Sep. 2016; coarse biogenic sediment in a pool among rocks covered by coralligenous formations; grab; HELM project (sample NG40_2M) • 1 spcm; Ashqelon; 31.7487°N, 34.4960°E; depth 41 m; 18 Sep. 2016; sandy mud; grab; HELM project (sample SG40_1F).

###### Remarks.

This species is characterized by a straight, conical profile with flat whorls, separated by a deep, canaliculate suture; the abapical part of the whorl is angulated. The sculpture consists of straight axial ribs and a prominent suprasutural spiral cord; the base is smooth except for faint continuations of the axial ribs; an internal columellar fold is present and visible inside the aperture when slightly turning the shell to the left side. The protoconch is of type C and in the illustrated specimen it has a diameter of ~ 240 μm, which is slightly smaller than 270–290 μm stated by Peñas and Rolán (2017) for this species.

*Parthenina typica* (Laseron, 1959) was described from eastern Australia (Laseron 1959) and subsequently recorded from the Solomon Islands, Fiji and the Philippines at infralittoral to bathyal depths (Peñas and Rolán 2017). To our knowledge, it has not been reported from the Indian Ocean nor from the Red Sea, however, its absence could well represent an artifact of the limited knowledge of the micromollusk fauna of these regions. Among native species, the conchologically highly variable *Parthenina interstincta* (J. Adams, 1797) and *P. monozona* (Brusina, 1869) are most similar, however, they differ by having more rounded whorls and a greater number of axial ribs.

**Figure 27.**
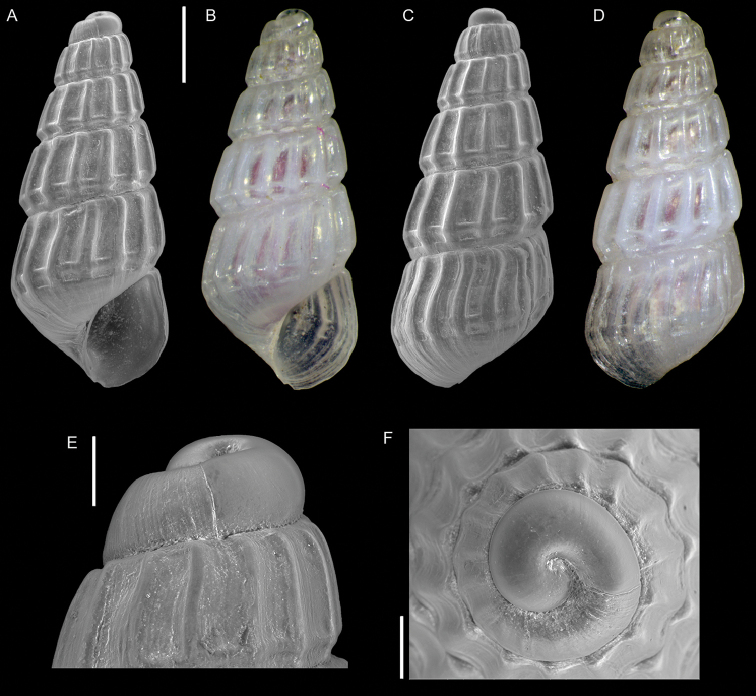
*Parthenina typica* (Laseron, 1959), Palmachim, Israel, Shafdan project: front (**A, B**) and back (**C, D**) views, apex (**E**) and apical view of the protoconch (**F**). Scale bars: 0.4 mm (**A–D**); 0.1 mm (**E, F**).

##### Pyrgulina craticulata

Taxon classificationAnimaliaPyramidellidae

(Issel, 1869)

FCC5754D-54D5-5A0F-9AAB-1C98D3BF3632

Figure 28A–I

###### New records.

Israel • 1 sh; north of Atlit; 32.7820°N, 34.9466°E; depth 10 m; 21 Sep. 2016; sand; grab; HELM project (sample NG10_1F) • 3 spcms; Ashqelon; 31.6868°N, 34.5516°E; depth 12 m; 30 Apr. 2018; offshore rocky reef; suction sampler; HELM project (samples S12_2F, S12_3F) • 17 spcms, 1 sh; same collecting data as for preceding; depth 11 m; 31 Oct. 2018; HELM project (samples S58_1F, S58_1M, S58_2F, S58_3F); size: H 1.5 mm, W 0.7 mm (illustrated specimen) • 2 spcms; Ashqelon; 31.6891°N, 34.5257°E; depth 25 m; 2 May 2018; offshore rocky reef; suction sampler; HELM project (sample S16_2F) • 1 spcm; same collecting data as for preceding; depth 28 m; 31 Oct. 2018; HELM project (sample S59_2F) • 2 spcms; west of Rosh HaNikra Islands; 33.0704°N, 35.0926°E; depth 12 m; 29 Oct. 2018; rocky substrate; suction sampler; HELM project (samples S52_2F, S52_3F) • 1 spcm; west of Rosh HaNikra Islands; 33.0725°N, 35.0923°E; depth 20 m; 1 May 2018; rocky substrate; suction sampler; HELM project (sample S13_3F) • 2 spcms; same collecting data as for preceding; depth 19 m; 29 Oct. 2018; HELM project (sample S53_1F).

###### Additional material examined.

*Pyrgulina craticulata*: EGYPT • 1 sh; Sinai (Red Sea), Gulf of Aqaba, Strait of Tiran, Jackson Reef; 28.0167°N, 34.4667°E; depth 2–3 m; 31 Oct. 1985; sand and coral substrate; T. Bratcher leg.; LACM 1985-111.4.

Madagascar • 3 shs; Antsiranana Province, south of Nosy Be, out from Hellville (= Andoany); 13.4500° S, 48.2500°E; depth 14 m; 5 Apr. 1989; coral heads and gorgonians; J.H. McLean leg.; LACM 1989-55.1.

Maldives • 1 sh; North Male Atoll, 2 km north of Baros Island; 4.3167°N, 73.4167°E; depth 25–35 m; 10 Feb. 1983; gravel; leg. A.J. Ferreira; LACM 1983-9.7.

Thailand • 1 sh; Phuket Province, east side of Kaew Yai Island; 7.7450°N, 98.3083°E; depth 0–3 m; 24–26 Mar. 1985; coral; J.H. McLean leg.; LACM 1985-5.17 • 1 sh; same collecting data as for preceding; LACM 1985-5.18 • 6 shs; Phuket Province, southern tip of Phromthep Cape; 7.7583°N, 98.3217°E; depth 0 m; 24 Mar. 1985; boulder beach with coral; J.H. McLean leg.; LACM 1985-6.4 • 4 shs; Phuket Province, east of Phuket, near Pee-Pee (= Phi Phi) Island, Hin Hmusang Rock; 7.7917°N, 98.5500°E; depth 15 m; 15 Feb. 1985; sand; A.J. Ferreira leg.; LACM 1985-14.2.

*Spiralinella incerta* (Milaschewitsch, 1916): Israel • 1 sh; north of Atlit; 32.7820°N, 34.9466°E; depth 10 m; 21 Sep. 2016; sand; grab; HELM project (sample NG10_1F) • 1sh; north of Atlit; 32.7422°N, 34.9181°E; depth 30 m; 20 Sep. 2016; sand; grab; HELM project (sample NG30_2F) • 2 shs; Ashqelon; 31.7002°N, 34.5498°E; depth 21 m; 18 Sep. 2016; sand; grab; HELM project (sample SG20_2F) • 1 sh; Ashqelon; 31.7101°N, 34.5406°E; depth 31 m; silty sand, 90 cm below sediment surface; gravity corer; HELM project (sample SC30_1_90L) • 1 sh; west of Rosh HaNikra Islands; 33.0704°N, 35.0926°E; depth 12 m; 1 May 2018; rocky substrate; suction sampler; HELM project (sample S14_4F); size: H 1.5 mm, W 0.7 mm (illustrated shell).

###### Remarks.

Issel (1869) described *Odontostomia craticulata* based on an illustration of a shell from the Red Sea by Savigny (1817: pl. 3, fig. 39); the syntype from the Savigny collection (MNHN-IM-2000-34095) was later figured by Bouchet & Danrigal (1982) and is shown on the MNHN website (https://science.mnhn.fr/institution/mnhn/collection/im/item/2000-34095?listIndex=15&listCount=44; last accessed 26 July 2020). As *Odontostomia* is considered an unjustified emendation of *Odostomia* (MolluscaBase 2020), a genus with very different conchological characters from *P. craticulata*, we follow the opinion of Peñas et al. (2020) and use the combination *Pyrgulina craticulata* (Issel, 1869).

*Pyrgulina craticulata* has been reported only from the Red Sea so far, however, a search of previously uncatalogued lots of pyramidellid shells in the LACM collection by one of us (PILF) yielded specimens from Madagascar, the Maldives and Thailand, confirming that this species has a much wider distribution in the Indian Ocean. Shells of *P. craticulata* seem indistinguishable from the illustration of *Chrysallida tribulationis* (Hedley, 1909), a taxon recorded from Australia and the western Japan Sea (Hedley 1909; Higo et al. 2001). In the light of a possible distribution of *P. craticulata* also in the West Pacific, we recommend an assessment of potential synonymy between *C. tribulationis* and *P. craticulata*.

**Figure 28.**
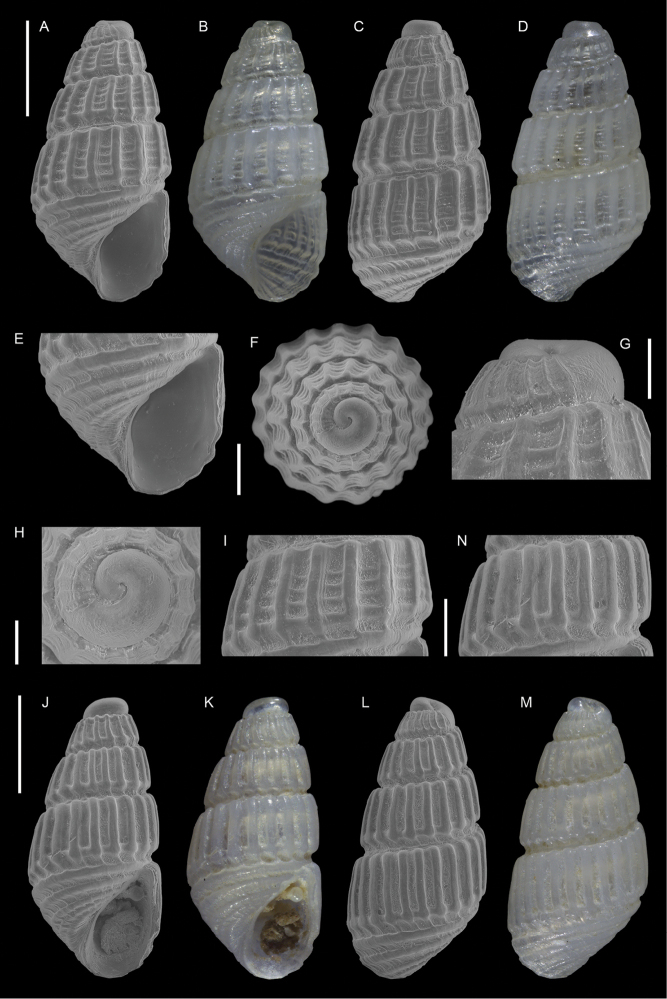
Comparison between *Pyrgulina craticulata* (Issel, 1869) and *Spiralinella incerta* (Milaschewitsch, 1916) **A–I***Pyrgulina craticulata*, Ashqelon, Israel, HELM project (sample S58_3F): front (**A, B**) and back (**C, D**) views, aperture and details of the sculpture on the base (**E**), top view (**F**), detail of the protoconch-teleoconch transition (**G**), apical view of the protoconch (**H**) and detail of the sculpture of the body whorl (**I**) **J–N***Spiralinella incerta*, west of Rosh HaNikra Islands, Israel, HELM project (sample S14_4F): front (**J, K**) and back **(L, M**) views, detail of the sculpture of the body whorl (**N**). Scale bars: 0.5 mm (**A–D, J–M**); 0.2 mm (**E, F, I, N**); 0.1 mm (**G, H**).

Here, we provide the first records of *P. craticulata* for the Mediterranean Sea. Several living specimens were found on hard substrates off southern and northern Israel, suggesting it is established in the region. In terms of shell size, shape and type of ornamentation, this species closely resembles the native *Spiralinella incerta* (Figure 28J–N), but has pronounced spiral cords in the interspaces of the axial ribs (Figure 28I vs. 28N) which enable a reliable segregation of these two species. In addition, the axial ribs are spaced more closely in *S. incerta* than in *P. craticulata*.

##### Pyrgulina nana

Taxon classificationAnimaliaPyramidellidae

Hornung & Mermod, 1924

3F66815F-57EB-5BEE-84DD-5A05F7032F1F

###### New records.

Israel • 2 spcms; Ashqelon; 31.6891°N, 34.5257°E; depth 28 m; 31 Oct 2018; offshore rocky reef; suction sampler; HELM project (sample S59_2F) • 1 spcm; west of Rosh HaNikra Islands; 33.0704°N, 35.0926°E; depth 12 m; 1 May 2018; rocky substrate; suction sampler; HELM project (sample S14_4F) • 5 spcms; same collecting data as for preceding; 29 Oct. 2018; HELM project (samples S52_1F, S52_2F).

###### Remarks.

Until now, the first record of this species from the Mediterranean was considered to be by Öztürk and van Aartsen (2006), who reported on material obtained from shallow-water sediment samples collected along the Turkish Levantine (Viransehir, Mersin Bay) and Aegean coasts (Güllük Bay) in 1997 and 2000, respectively. However, already van der Linden and Eikenboom (1992) described and illustrated *P. nana* from the Levantine Sea (page 60, Figure 41), referring to it as *Chrysallida* spec. C in the lack of a species-level identification. Their material consisted of a single individual, likely an empty shell, but not specified by the authors, from Mersin (south-eastern Turkey) with unknown collecting date, housed in the collection of J. van der Linden (The Hague, The Netherlands). Although we were unable to examine this material, the excellent and detailed line drawing provided enabled an unambiguous assignment of *Chrysallida* spec. C to *P. nana*; thus, van der Linden and Eikenboom (1992) should be regarded the first Mediterranean record. Today, the known Mediterranean distribution of *P. nana* includes Turkey, Lebanon, and Israel (Bogi and Galil 2006; Giannuzzi-Savelli et al. 2014). To our knowledge, this is the first record of living individuals of *P. nana* from Israel; here, the species occurs along both the southern (Ashqelon) and northern coasts (west of Rosh HaNikra Islands) on rocky bottoms at 12–28 m depth.

##### Turbonilla funiculata

Taxon classificationAnimaliaPyramidellidae

de Folin, 1868

F109EDBF-AB5F-541C-8EFC-008088D8BCED

Figure 29

###### New records.

Israel • 2 spcms; west of Rosh HaNikra Islands; 33.0704°N, 35.0926°E; depth 12 m; 1 May 2018; rocky substrate; suction sampler; HELM project (sample S14_4F) • 15 spcms; same collecting data as for preceding; 29 Oct. 2018; HELM project (samples S52_1F, S52_2F, S52_3F); size: H 1.8 mm, W 0.6 mm (illustrated specimen).

###### Additional material examined.

China • 10 shs; Hong Kong, Tolo Channel, cove at Hoi Sing Wan; 22.430°N, 114.2467°E; depth 0 m; 6 Apr. 1985; sand, rock and oysters; J.H. McLean leg.; LACM 1985-12.2.

Pakistan • 4 shs; Sind Province, small cove 4.8 km west of atomic power plant and 7 km west-northwest of Bulegi Point; 24.8000°N, 66.7250°E; depth 0–4 m; 19 Jan. 1979; rock and clay; C.C. Swift leg.; LACM 1979-1.3.

Sri Lanka • 1 sh; Southern Province, Tangalla, cove at Tangalla Bay Hotel; 6.0250°N, 80.8000°E; depth 0–1 m; 30–31 Jan. 1983; rock and clay; A.J. Ferreira leg.; LACM 1983-5.2.

Vietnam • 1 sh; station 1328 of the Nha-Trang Oceanography Institute: “Entre les îles des Pêcheurs, Hon-Mung, le banc du Castleragh et l’isobathe 50. (En dehors et au S. de la baie de Nha-Trang)” [between Îles des Pêcheurs, Hon-Mung, Banc du Castleragh (= Castlereagh) and isobath 50. (outside of and to the south of the Bay of Nha-Trang)]; sediment sample; dredge; MNHN-IM-2000-21843 (holotype of *Pyrgiscus mirandus* Saurin, 1959).

**Figure 29.**
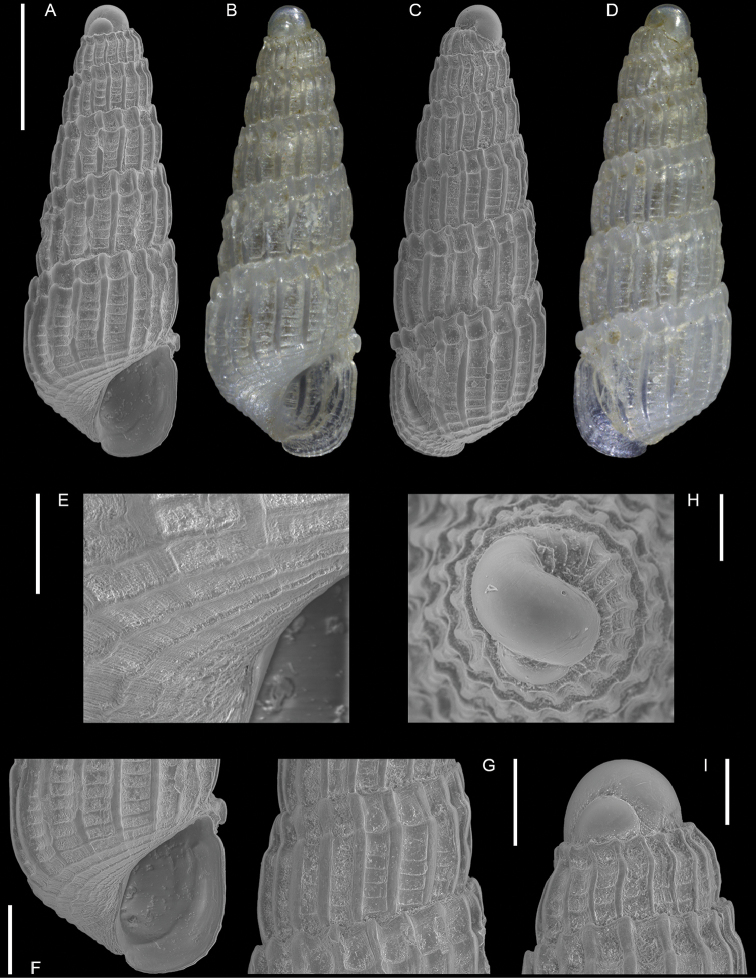
*Turbonilla funiculata* de Folin, 1868, west of Rosh HaNikra Islands, Israel, HELM project (sample S52_2F): front (**A, B**) and back (**C, D**) views, detail of the sculpture on the base (**E**), aperture (**F**), detail of the sculpture on the back of the third whorl (**G**), apical (**H**) and side (**I**) views of the protoconch. Scale bars: 0.5 mm (**A–D**); 0.1 mm (**E, H, I**); 0.2 mm (**F, G**).

###### Remarks.

Shells of *Turbonilla funiculata* are polymorphic with respect to their shape (Peñas and Rolán 2010 and our own observations) but the species can be readily distinguished from all pyramidellids in the Mediterranean – the most similar being the non-indigenous *Turbonilla edgarii* (Melvill, 1896), *Turbonilla flaianoi* Mazziotti, Agamennone, Micali & Tisselli, 2006 and *Pyrgulina fischeri* Hornung & Mermod, 1925 – by the presence of a very marked subsutural constriction running along the whorls. This constriction separates the pronounced, almost orthocline axial ribs into a larger lower and a narrow upper, crown-like, portion. The ribs extend adapically beyond the suture of the preceding whorl, giving the transition zone between subsequent whorls a wavy appearance. The interspaces of the lower portion of the ribs bear several thin spiral lines, while those between the narrow upper parts of the ribs are smooth. The protoconch is helicoid and of type A.

*Turbonilla funiculata* has been previously reported from Fiji, Hong Kong, Indonesia, New Caledonia, Thailand, the Solomon Islands and Vietnam from shore to 396 m depth (Robba et al. 2004; Peñas and Rolán 2010). As Robba et al. (2004) already pointed out, the shell figured as *Pyrgiscus microscopica* (Laseron, 1959) by Okutani (2000: 712, fig. 68) is most likely *T. funiculata*, confirming that the species also occurs in Japan. This interpretation is re-affirmed by another illustration of the very same Japanese specimen in Mazziotti et al. (2005: 81, fig. 1m, n), showing the subsutural constriction characteristic of *T. funiculata*. Shells of *T. funiculata* were found by one of us (PILF) also among hitherto unidentified lots of shells from Pakistan and Sri Lanka housed in the LACM collection, demonstrating that this species also lives in the Indian Ocean. Here, we report the first records of *T. funiculata* for the Mediterranean, where several living specimens were collected in northern Israel on hard substrates at 12 m depth.

#### Family Cylichnidae H. Adams & A. Adams, 1854

##### Cylichna collyra

Taxon classificationAnimaliaCephalaspideaCylichnidae

Melvill, 1906

58FB6303-705E-5F4E-B814-167FAC3BB7A9

Figure 30

###### New records.

Israel • 1 spcm; Palmachim; 31.9477°N, 34.6562°E; depth 37.5 m; 18 Oct. 2017; soft substrate; box-corer; Shafdan project (sample 22(A)); size: H 4.2 mm, W 1.5 mm (illustrated specimen) • 1 spcm; Palmachim; 31.9424°N, 34.6551°E; depth 36.3 m; 2 May 2018; soft substrate; box-corer; Shafdan project (sample 21(A)); size: H 7.2 mm, W 4.0 mm • 2 spcms; Palmachim; 31.9376°N, 34.6515°E; depth 36.7 m; 2 May 2018; soft substrate; box-corer; Shafdan project (sample 5(B)) • 1 spcm; Palmachim; 31.9685°N, 34.6732°E; depth 35.6 m; 2 May 2018; soft substrate; box-corer; Shafdan project (sample 26(A)) • 1 spcm; Palmachim; 31.9477°N, 34.6562°E; depth 37.5 m; 2 May 2018; soft substrate; box-corer; Shafdan project (sample 22(B)) • 3 spcms; Palmachim; 31.9424°N, 34.6551°E; depth 36.3 m; 2 May 2018; soft substrate; box-corer; Shafdan project (sample 21(C)) • 2 spcms; Palmachim; 31.9327°N, 34.6495°E; depth 36.2 m; 2 May 2018; soft substrate; box-corer; Shafdan project (sample 4(A)) • 1 spcm; Palmachim; 31.9574°N, 34.6645°E; depth 36.2 m; 2 May 2018; soft substrate; box-corer; Shafdan project (sample 24(C)) • 1 spcm; Palmachim; 31.9477°N, 34.6562°E; depth 37.5 m; 20 Oct. 2018; soft substrate; box-corer; Shafdan project (sample 22(A)).

###### Additional material examined.

Oman • 13 shs; off Muscat; 24°58'N, 56°54'E; 156 fathoms (285 m) depth; NMW.1955.158.00578 (F.W. Townsend coll.).

###### Remarks.

We record here for the first time in the Mediterranean 13 living individuals of *Cylichna collyra*, a cephalaspidean originally described from the Gulf of Oman (Melvill 1906b). *Cylichna collyra* can be distinguished from the native Mediterranean *C. cylindracea* (Pennant, 1777) by its more elongated and slender shell, the more tapering apical part, the color pattern characterized by fine brown spiral lines apically and abapically, and the smaller size (*C. cylindracea* commonly reaches 1 cm in height whereas *C. collyra* attains approximately half that size). *Cylichna villersii* (Audouin, 1826), another non-indigenous species of Red Sea origin recorded from the Mediterranean coast of Israel (Bogi and Galil 2013a), is smaller (less than 2 mm), less slender, has a more rounded base and stronger growth marks (not visible in *C. collyra*), and bears two brown bands apically and abapically instead of the fine brown lines. *Cylichna biplicata* (A. Adams in Sowerby, 1850), a species occurring on the continental platform in the Indo-West Pacific, shares with our specimens the cylindrical shape and the color pattern of reddish-brown spiral bands apically and abapically (Valdés 2008), but is larger, more elongated anteriorly, with a stronger columellar tooth, and the colored spiral bands become a compact larger band apically. *Cylichna collyra* has not been recorded from the Red Sea yet (Dekker and Orlin 2000).

**Figure 30.**
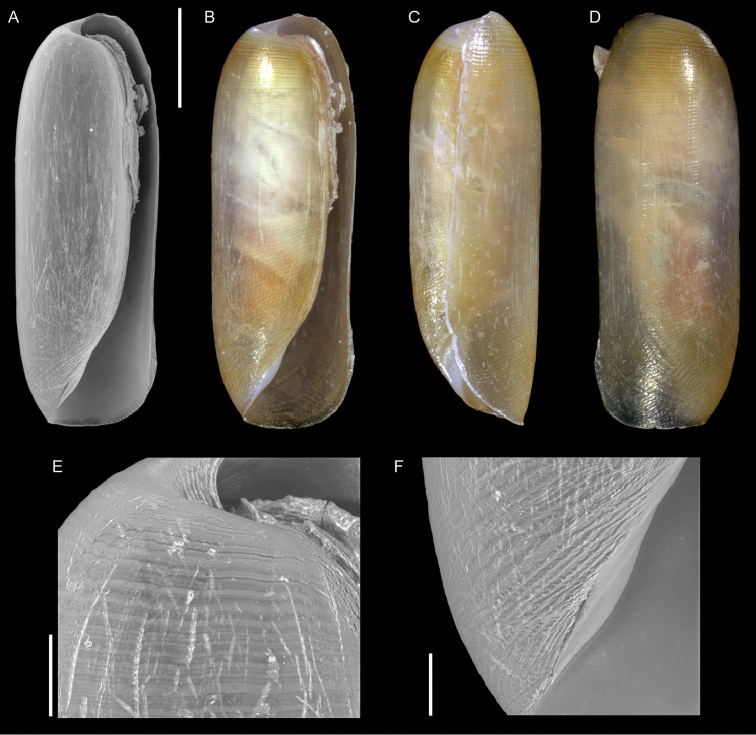
*Cylichna collyra* Melvill, 1906, Palmachim, Israel, Shafdan project (sample 22(A)): front (**A, B**), side (**C**) and back (**D**) views, and apical (**E**) and anterior (**F**) microsculpture. Scale bars: 1 mm (**A–D**); 0.2 mm (**E, F**).

#### Family Mnestiidae Oskars, Bouchet & Malaquias, 2015

##### Mnestia girardi

Taxon classificationAnimaliaCephalaspideaMnestiidae

(Audouin, 1826)

6A2831AD-6EA6-59C1-B1EE-E55C72D88C58

###### New records.

Israel • 5 spcms; west of Rosh HaNikra Islands; 33.0704°N, 35.0926°E; depth 12 m; 29 Oct. 2018; HELM project (samples S52_1F, S52_2F, S52_3F) • 1 spcm, 2 shs; west of Rosh HaNikra Islands; 33.0725°N, 35.0923°E; depth 20 m; 1 May 2018; rocky substrate; suction sampler; HELM project (samples S13_2M, S13_3F) • 12 spcms; same collecting data as for preceding; depth 19 m; 29 Oct. 2018; HELM project (samples S53_1F, S53_2F, S53_2M, S53_3F).

###### Remarks.

This species was first recorded in the Mediterranean Sea in 1974 with the finding of few empty shells in the Bardawil Lagoons in Egypt (Mienis 1976). The species has since been recorded also in Greece (Crocetta et al. 2017), Turkey (Çinar et al. 2011), Cyprus (Katsanevakis et al. 2009), and Lebanon (Crocetta et al. 2020). A single record is available from Israel based on shells collected in 2004 off Palmachim (Bogi and Galil 2006). We here report living individuals from Israel for the first time. This is also the first finding of living individuals in the Mediterranean Sea.

#### Family Haminoeidae Pilsbry, 1895

##### Atys angustatus

Taxon classificationAnimaliaCephalaspideaHaminoeidae

E.A. Smith, 1872

BDBF63B8-74DE-5D67-A451-9858397F7849

Figure 31

###### New records.

Greece • 1 sh; Crete, Plakias; 35.1796°N, 24.3957°E; depth 15 m; 21 Sep. 2017; *Posidonia oceanica* rhizomes; suction sampler (sample Rh.15_5M); size: H 2.6 mm, W not available because of broken aperture.

###### Remarks.

*Atys angustatus* was first recorded in the Mediterranean Sea in 1974, based on specimens collected at Haifa, Israel (Aartsen and Goud 2006). It has been reported from Mersin, Turkey, since 1986 (Aartsen and Goud 2006) and multiple records from Israel followed from several locations along its coast (Micali et al. 2016). To the best of our knowledge, this is the first record from Greece.

**Figure 31.**
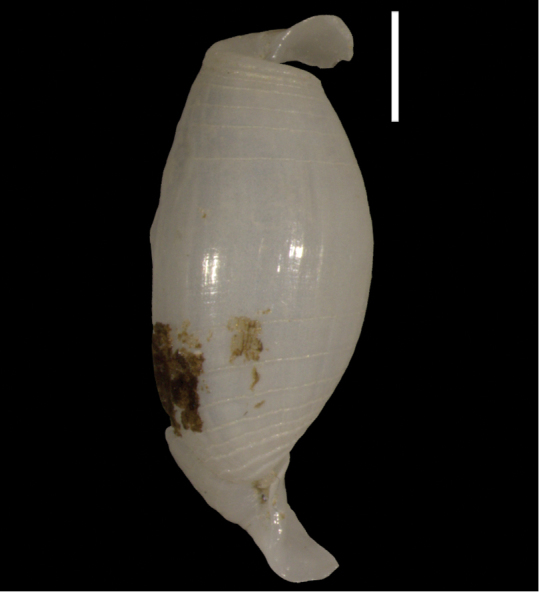
*Atys angustatus* E.A. Smith, 1872, Plakias, Crete, Greece (sample Rh.15_5M): front view. Scale bar: 0.5 mm.

### Class Bivalvia Linnaeus, 1758

#### Family Mytilidae Rafinesque, 1815

##### Arcuatula perfragilis

Taxon classificationAnimaliaMytilidaMytilidae

(Dunker, 1857)

4EFC6F1B-5BAE-526D-9E09-BBEA5C3EDA67

Figure 32

###### New records.

Israel • 1 spcm; Palmachim; 31.9574°N, 34.6645°E; depth 36.2 m; 2 May 2018; soft substrate; box-corer; Shafdan project (sample 24(C)); size: L 13 mm, H 5.3 mm • 1 spcm; Palmachim; 31.9737°N, 34.6767°E; depth 35.4 m; 2 May 2018; soft substrate; box-corer; Shafdan project (sample 29(A)).

###### Remarks.

This species was first recorded in Israel in 1960 from Bat Yam with living individuals (Barash and Danin 1973). Further living individuals were recorded in the 1960s and early 1970s from Israel and Egypt (Bardawil Lagoons) (Barash and Danin 1971, 1973, 1977). Since then, however, no further living individuals were found, questioning the persistence of its populations in the Mediterranean. We report here the recent finding of two living individuals, suggesting that the species indeed still occurs in Israel.

**Figure 32.**
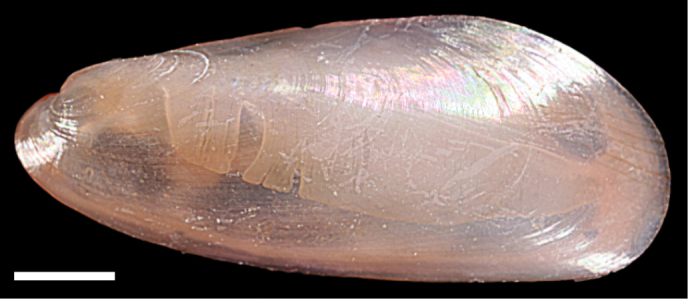
*Arcuatula perfragilis* (Dunker, 1857), Palmachim, Israel, Shafdan project. Left valve outer view. The pink hue is due to staining with eosin solution. Photograph courtesy S. Bartolini. Scale bar: 2 mm.

##### Lioberus ligneus

Taxon classificationAnimaliaMytilidaMytilidae

(Reeve, 1858)

1B4E9E63-7046-5180-94A2-F8F78A61DFD4

Figure 33A–D

###### New records.

Cyprus • 2 shs; Turkish Republic of Northern Cyprus, southern tip of Karpaz Peninsula; 35.6809°N, 34.5785°E; depth 1 m; 29 Jul. 2019; mixed substrate with sand, rocks and *Cystoseira*; P.G. Albano leg. (sample NCY7H); NHMW-MO-112930/LM/0173; size of the largest shell: L 30.2 mm, H 15.0 mm.

Israel • 1 spcm; Carmel Head; 32.8232°N, 34.9431°E; depth 12 m; 21 Apr. 2020; in a small patch of *Galaxaura rugosa* and *Cystoseira* sp.; M. Mulas leg. (sample SK2); SMNH MO83605; size: L 17.6 mm, H 10.0 mm.

###### Additional material examined.

*Lioberus ligneus*: Egypt • 19 vv; Red Sea, Suez; Jousseaume coll.; MNHN • 1 sh; Sinai (Red Sea), Dahab, dive site “Islands”; 28.4789°N, 34.5126°E; beached; Nov. 2007; H. Blatterer leg. • 1 v; same collecting data as for preceding; 2012 • 1 v; Sinai (Red Sea), Dahab, dive site “Lagoon”; depth unspecified; 2008; H. Blatterer leg. • 1 v; same collecting data as for preceding; 2009.

*Lioberus agglutinans* (Cantraine, 1835): Portugal • 1 v; Algarve, Canal of Olhão; depth 3–7m, MNHN • 2 vv; Algarve, Tavira, Pedra do Barril; depth 25 m; MNHN • 1 sh, 1 v; Algarve, Sagres, Ponta da Baleeira; depth 17–23 m; MNHN • 1 spcm; Algarve, Tavira, off Cabanas; depth 14 m; MNHN • 2 shs; Algarve, Olhão, Ilha da Barretta; depth unspecified; MNHN.

Tunisia • 1 sh, 8 vv; Gulf of Gabès, NW Boughrara Gulf; depth 10–15 m; MNHN • 11 spcms, 42 vv; Gulf of Gabès, Djerba, Ajim Canal; depth 10–32 m; MNHN.

###### Remarks.

We here report *Lioberus ligneus* for the first time from Israel and Cyprus. The Israeli live-collected specimen comes from a patch of *Galaxaura rugosa* and *Cystoseira* sp. whereas the two empty but fresh shells from Cyprus were found attached vertically to *Cystoseira* shoots. These findings suggest that this species indeed prefers vegetated habitats in the shallow subtidal. This species has been previously reported from Lebanon based on shells collected in 1999–2000 (Crocetta et al. 2013), suggesting that it has likely occurred undetected throughout the Levantine Basin for long. The distinction of *L. ligneus* from the native Mediterranean *Lioberus agglutinans* (Cantraine, 1835) is not straightforward because both species share the elongated appearance, sculpture limited to concentric striae, and brown color. Moreover, they are morphologically variable. A fairly consistent character of *L. ligneus* in the samples we inspected from the Red Sea is the darker internal color, often shading into violet. The group would deserve a taxonomic revision to unambiguously distinguish its species.

**Figure 33.**
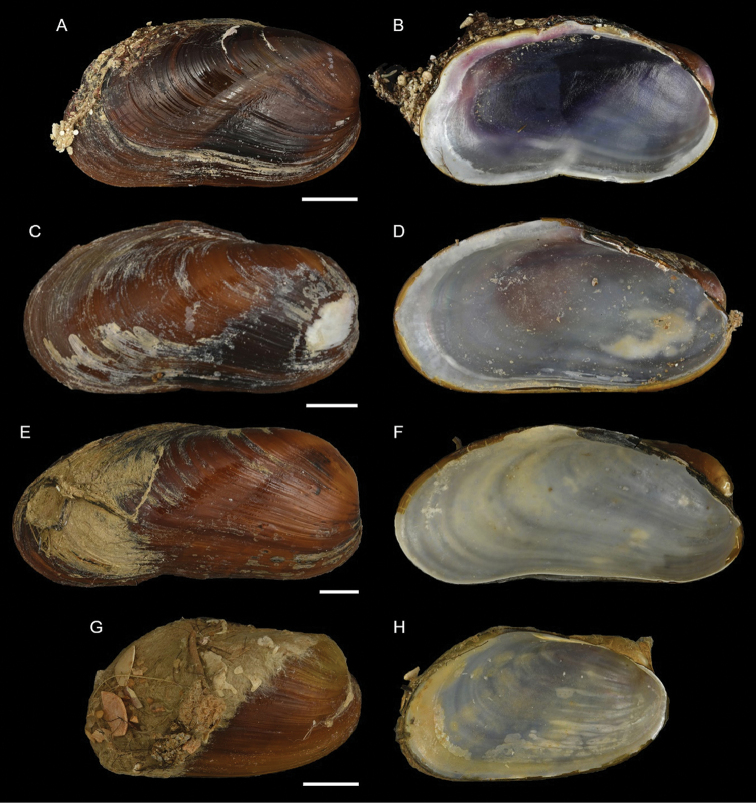
Comparison between *Lioberus ligneus* (Reeve, 1858) and *Lioberus agglutinans* (Cantraine, 1835). **A, B***Lioberus ligneus*, NHMW–MO-112930/LM/0173, Karpaz Peninsula, Turkish Republic of Northern Cyprus, Cyprus: right valve outer (**A**) and left valve inner (**B**) views **C, D***L. ligneus*, Dahab, Egypt (H. Blatterer coll.): right valve outer (**C**) and left valve inner (**D**) views **E, F***Lioberus agglutinans*, Olhão, Ilha da Barretta, Algarve, Portugal: right valve outer (**E**) and left valve inner (**F**) views **G, H***L. agglutinans*, Tavira, off Cabanas, Algarve, Portugal: right valve outer (**G**) and left valve inner (**H**) views. Scale bars: 5 mm.

##### Musculus coenobitus

Taxon classificationAnimaliaMytilidaMytilidae

(Vaillant, 1865)

93B8662F-6415-573A-B6FD-1EFCB0AF1E21

**Figure 34.**
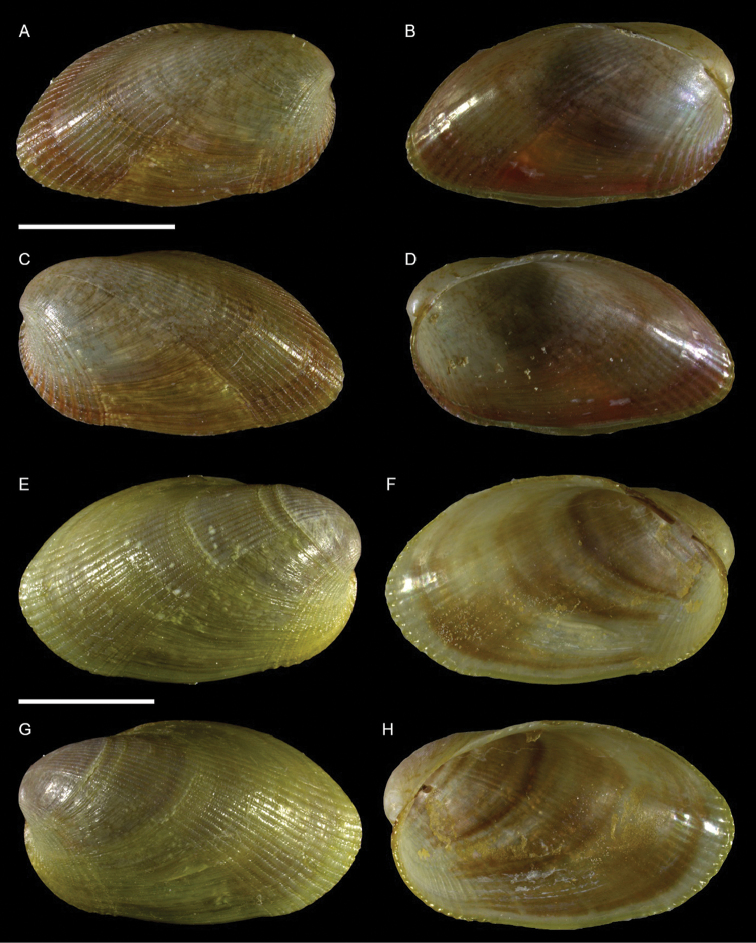
Comparison between *Musculus coenobitus* (Vaillant, 1865) and *Musculus subpictus* (Cantraine, 1835) **A–D***Musculus coenobitus*, north of Nahariyya port, Israel, HELM project (sample H3): right valve outer (**A**) and inner (**D**) views, left valve inner (**B**) and outer (**C**) views **E–H***Musculus subpictus*, off Filfla, Malta: right valve outer (**E**) and inner (**H**) views, left valve inner (**F**) and outer (**G**) views. Scale bars: 5 mm.

**Figure 35.**
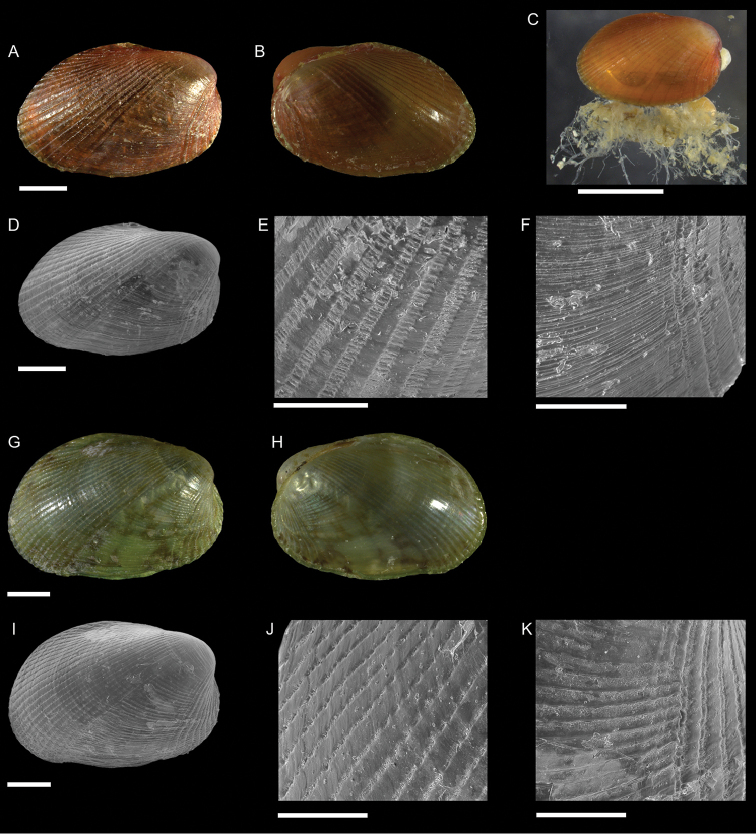
Comparison between *Musculus coenobitus* (Vaillant, 1865) and *Musculus subpictus* (Cantraine, 1835) **A–F***Musculus coenobitus*, west of Rosh HaNikra Islands, Israel, HELM project (sample S52_2L): right valve outer (**A, D**) and inner (**B**) views, living specimen with byssus (**C**), sculpture of the posterior (**E**) and anterior (**F**) area of the right valve **G–K***Musculus subpictus*, Ashqelon, Israel, HELM project (sample S12_1L): right valve outer (**G, I**) and inner (**H**) views, sculpture of the posterior (**J**) and anterior (**K**) area. Scale bars: 1 mm (**A, B, D, G–I**), 2.5 mm (**C**); 0.5 mm (**E, F, J, K**).

Figures 34A–D , 35A–F

###### New records.

Israel • 2 spcms; west of Rosh HaNikra Islands; 33.0704°N, 35.0926°E; depth 12 m; 1 May 2018; rocky substrate; suction sampler; HELM project (sample S14_4M) • 4 spcms; same collecting data as for preceding; 29 Oct. 2018; HELM project (samples S52_1M, S52_2L, S52_3M, S52_3L) • 1 sh; north of Nahariyya port; 33.0127°N, 35.0896°E; beached; 29 Oct. 2018; HELM project (sample H3); size: L 10.2 mm, H 6.0 mm (illustrated shell, Figure 34A–D).

###### Additional material examined.

*Musculus subpictus* (Cantraine, 1835): Croatia • 14 spcms; Istria, off Rovinj, western shore of Sveta Katarina; 45.0760°N, 13.6276°E; 3 Jul. 2008; dried-out fouling community collected from the surface of buoy-like floating objects dumped on the shore (J. Steger coll.).

Israel • 10 spcms, 3 vv; Ashqelon; 31.6868°N, 34.5516°E; depth 12 m; 30 Apr. 2018; offshore rocky reef; suction sampler; HELM project (samples S12_1F, S12_1M, S12_1L, S12_2F, S12_2M) • 111 spcms, 2 vv; Ashqelon; 31.6891°N, 34.5257°E; depth 25 m; 2 May 2018; offshore rocky reef; suction sampler; HELM project (samples S16_1F, S16_1M, S16_1L, S16_2F, S16_2M, S16_2L) • 13 spcms; west of Rosh HaNikra Islands; 33.0704°N, 35.0926°E; depth 12 m; 1 May 2018; rocky substrate; suction sampler; HELM project (samples S14_2F, S14_2M, S14_3F, S14_4F) • 1 v; west of Rosh HaNikra Islands; 33.0725°N, 35.0923°E; depth 20 m; 1 May 2018; rocky substrate; suction sampler; HELM project (sample S13_3M) • 1 spcm; same collecting data as for preceding; depth 19 m; 29 Oct. 2018; HELM project (sample S53_2L).

Malta • 9 spcms; off Filfla Island; on a rafting fisherman’s net; 1981 (P.G. Albano coll. ID 557).

###### Remarks.

We found several living individuals of *Musculus coenobitus* in northern Israel. It is the first record of this Red Sea species for the Mediterranean Sea. *Musculus coenobitus* is very similar to the native *M. subpictus*, which can occur sympatrically, but can be distinguished at once because of its reddish rather than greenish hue (Figure 34). It is also more elongated with a more pointed posterior margin. Juvenile specimens are bright red (Figure 35C), have interspaces between the radial riblets in the posterior part of the shell almost as large as the riblets themselves, while in *M. subpictus* the riblets are closely arranged with very narrow interspaces (Figure 35E, J). *Musculus coenobitus* also shows a fine but distinct lamellar concentric sculpture in such interspaces, whereas *M. subpictus* shows more sparse fine concentric ridges (Figure 35E and J, respectively). The relation of *M. coenobitus* with other Indo-Pacific species like *M. cumingianus* (Reeve, 1857) and *M. cuneatus* (Gould, 1861) deserves a revision (Oliver 1992).

##### Musculus aff. viridulus

Taxon classificationAnimaliaMytilidaMytilidae

(H. Adams, 1871)

A0903FAE-8EAA-5AF6-A23A-69715849109C

Figure 36A–E

###### New records.

**Figure 36.**
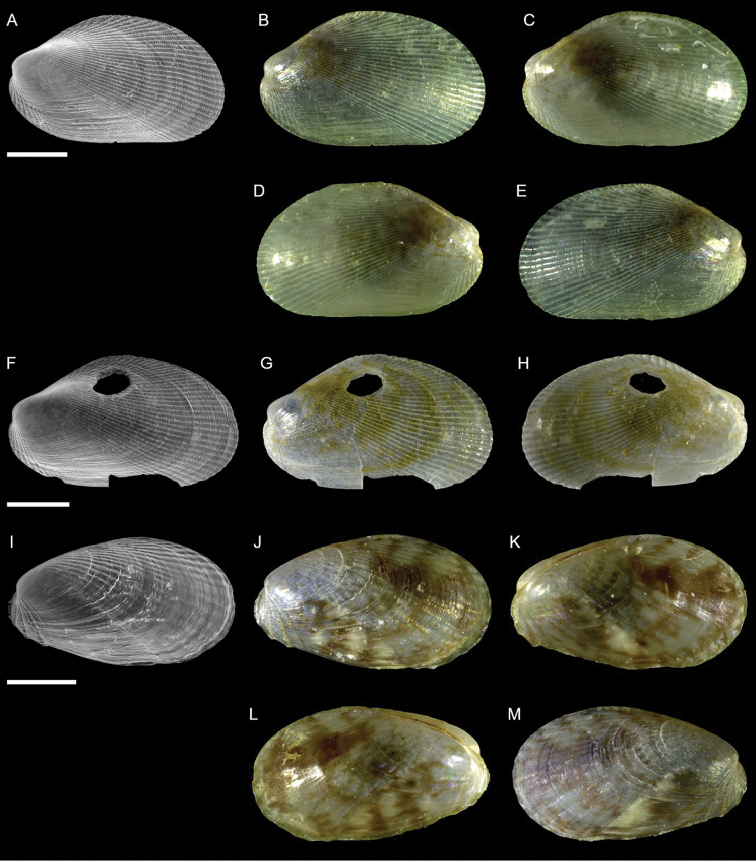
Comparison between Musculus aff. viridulus (H. Adams, 1871) and *Musculus costulatus* (Risso, 1826) **A–E**Musculus aff. viridulus, west of Rosh HaNikra Islands, Israel, HELM project (sample S52_3L): left valve outer (**A, B**) and inner (**D**) views, right valve inner (**C**) and outer (**E**) views **F–H**Musculus aff. viridulus, dive site “Caves”, Dahab, Sinai, Egypt: left valve outer (**F, G**) and inner (**H**) views **I–M***Musculus costulatus*, west of Rosh HaNikra Islands, Israel, HELM project (sample S53_2L): left valve outer (**I, J**) and inner (**L**) views, right valve inner (**K**) and outer (**M**) views. Scale bars: 1 mm.

Israel • 1 spcm; Ashqelon; 31.6891°N, 34.5257°E; depth 25 m; 2 May 2018; offshore rocky reef; suction sampler; HELM project (sample S16_2F) • 15 spcms; same collecting data as for preceding; depth 28 m; 31 Oct. 2018; HELM project (samples S59_1F, S59_1M, S59_2F, S59_2M) • 2 spcms; west of Rosh HaNikra Islands; 33.0704°N, 35.0926°E; depth 12 m; 1 May 2018; rocky substrate; suction sampler; HELM project (samples S14_3F, S14_4M) • 25 spcms; same collecting data as for preceding; 29 Oct. 2018; HELM project (samples S52_1F, S52_2F, S52_2M, S52_2L, S52_3F, S52_3M, S52_3L); size: L 3.6 mm, H 2.1 mm (illustrated specimen, Figure 36A–E) • 1 spcm; west of Rosh HaNikra Islands; 33.0725°N, 35.0923°E; depth 20 m; 1 May 2018; rocky substrate; suction sampler; HELM project (sample S13_3M) • 9 spcms; same collecting data as for preceding; depth 19 m; 29 Oct. 2018; HELM project (samples S53_1F, S53_2F, S53_2M, S53_3F, S53_3M).

###### Additional material examined.

Musculus aff. viridulus: Egypt • 1 v; Sinai (Red Sea), Dahab, dive site “Caves”; 28.416°N, 34.456°E; depth 15 m; 2015; H. Blatterer leg. • 4 vv; same collecting data as for preceding; depth 17–19 m; 2016; H. Blatterer leg. • 2 vv; same collecting data as for preceding; depth 18 m; 2017; H. Blatterer leg. • 1 v; Sinai (Red Sea), Dahab, dive site “Golden Blocks”; 28.436°N, 34.459°E; depth 18 m; 2018; H. Blatterer leg.

*Musculus costulatus* (Risso, 1826): Israel • 9 spcms, 4 vv; Ashqelon; 31.6868°N, 34.5516°E; depth 12 m; 30 Apr. 2018; offshore rocky reef; suction sampler; HELM project (samples S12_1F, S12_1M, S12_1L, S12_2F, S12_2M) • 3 spcms; Ashqelon; 31.6891°N, 34.5257°E; depth 25 m; 2 May 2018; offshore rocky reef; suction sampler; HELM project (samples S16_1F, S16_2F) • 1 spcm; same collecting data as for preceding; depth 28 m; 31 Oct. 2018; HELM project (sample S59_1M) • 5 spcms; west of Rosh HaNikra Islands; 33.0704°N, 35.0926°E; depth 12 m; 1 May 2018; rocky substrate; suction sampler; HELM project (samples S14_1L, S14_3F, S14_3L, S14_4F) • 56 spcms; same collecting data as for preceding; 29 Oct. 2018; HELM project (samples S52_1F, S52_1M, S52_1L, S52_2F, S52_2M, S52_2L, S52_3F, S52_3M, S52_3L) • 1 v; west of Rosh HaNikra Islands; 33.0725°N, 35.0923°E; depth 20 m; 1 May 2018; rocky substrate; suction sampler; HELM project (sample S13_3F) • 20 spcms; same collecting data as for preceding; depth 19 m; 29 Oct. 2018; HELM project (sample S53_2L).

###### Remarks.

We report numerous living individuals of Musculus aff. viridulus from the Mediterranean Israeli coastline. It is the first record of this Indo-Pacific species in the Mediterranean Sea. This species can be readily distinguished from the native *M. costulatus* because the latter has a more oval outline and a much smaller number of riblets at the same overall shell size (Figure 36). These riblets are also much larger compared to M. aff. viridulus. The Atlanto-Mediterranean *M. discors* (Linnaeus, 1767) has a similarly fine posterior sculpture but at the same size is much higher and at all sizes bears much more prominent riblets anteriorly. The taxonomy of *Musculus* in the Indo-Pacific province is not settled and the available images of Red Sea *M. viridulus* (Oliver 1992; Zuschin and Oliver 2003) show a more oval species, hence our dubitative identification. Still, we are confident that this is a Red Sea species because we examined indistinguishable specimens from the northern Red Sea (Figure 36F–H). Blatterer (2019) illustrated these and other similar specimens (plate 10, fig. 18a, b) as *Gregariella ehrenbergi* (Issel, 1869). We recorded a morphologically distinct species as *G. ehrenbergi* from a buoy stranded on the Israeli coastline (Steger et al. 2018; Ivkić et al. 2019). *Gregariella ehrenbergi* type material is corroded by Byne’s disease and the original description likely refers to a juvenile specimen; the identity of this species deserves further scrutiny.

#### Family Isognomonidae Woodring, 1925 (1828)

##### Isognomon aff. australica

Taxon classificationAnimaliaPterioidaIsognomonidae

(Reeve, 1858) (sensu Angelidis and Polyzoulis 2018)

F577854B-80CB-5AAD-9B6A-6E463F5F2286

Figure 37

###### New records.

**Figure 37.**
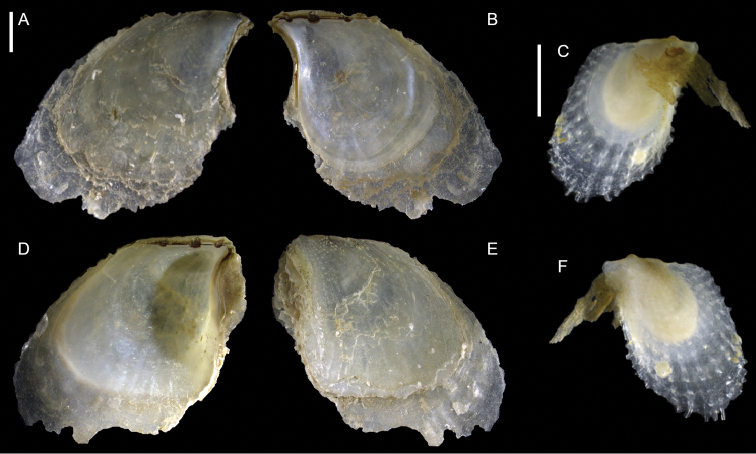
Isognomon aff. australica (Reeve, 1858) (sensu Angelidis and Polyzoulis 2018) **A, B, D, E** Esentepe, Cyprus (sample NCY3H): left valve outer (**A**) and inner (**B**) views, right valve inner (**D**) and outer (**E**) views **C, F** Plakias, Crete (sample Rh.05_4M): left (**C**) and right (**F**) valve outer views. Scale bars: 2 mm.

Cyprus • 1 sh; Turkish Republic of Northern Cyprus, Esentepe; 35.3589°N, 33.6078°E; depth 2 m; 28 Jul. 2019; rocky substrate; P.G. Albano leg. (sample NCY3H); size: L 11.8 mm, H 10.1 mm (Figure 37A, B, D, E).

Greece • 1 spcm; Crete, Plakias; 35.1796°N, 24.3957°E; depth 5 m; 24 Sep. 2017; *Posidonia oceanica* rhizomes; suction sampler (sample Rh.05_4M); size: L 4.4 mm, H 4.4 mm (Figure 37C, F).

###### Remarks.

The discrimination and identification of the species of *Isognomon* Lightfoot, 1786 is difficult due to their morphological plasticity that is related to their cryptic way of life. Still, the specimens recently reported from Astypalaia, in the Eastern Aegean Sea (Lipej et al. 2017; Angelidis and Polyzoulis 2018), show clear morphological differences from *I. legumen* (Gmelin, 1791), the established non-indigenous species in the Mediterranean Sea: the main features of the sculpture are radial rather than concentric and the shape of the shell can be very elongated rather than subquadrate. We here report a juvenile living individual and an empty shell from Greece and Cyprus (for which this is a new record), respectively, which are not distinguishable from those previously reported from Astypalaia. Juvenile shells (up to a size of ~ 5 mm) bear a sculpture of radial ribs adorned by tubular spines. We do not comment upon the choice of the *australica* name for this taxonomic entity by previous authors, lamenting the lack of a thorough revision of this genus in the Indo-Pacific province.

#### Family Lucinidae J. Fleming, 1828

##### Pegophysema cf. philippiana

Taxon classificationAnimaliaLucinidaLucinidae

(Reeve, 1850)

BEDC4EC9-EDAD-55E2-9C1D-E425AB2C4F6E

Figure 38

###### New records.

Israel • 1 spcm; Palmachim; 31.9574°N, 34.6645°E; depth 36.2 m; 2 May 2018; soft substrate; box-corer; Shafdan project (sample 24(B)); size: L 15 mm, H 13.5 mm.

###### Remarks.

*Pegophysema philippiana* was first found in the Mediterranean Sea in 2018 as a single valve from south of Tel Aviv (Mienis 2019). The specimen here reported is much smaller, and we acknowledge that the identification of juvenile individuals of this genus can only be tentative. We do not consider it conspecific to native species such as *Loripinus fragilis* (Philippi, 1836) because this latter species is much more inflated at this size, nor *Loripes orbiculatus* Poli, 1795 which has a different valve profile. If the identification is confirmed, this would be the first live collected specimen of *P. philippiana* in the Mediterranean Sea.

**Figure 38.**
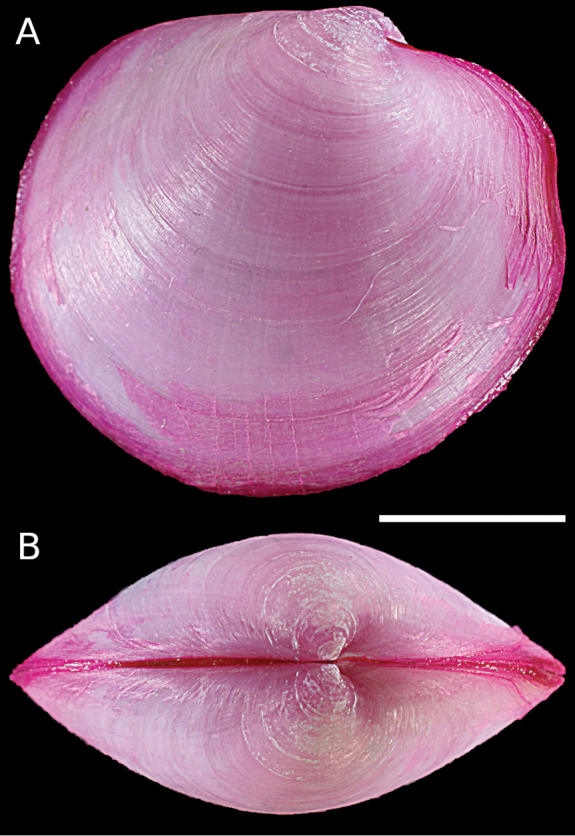
Pegophysema cf. philippiana (Reeve, 1850), Palmachim, Israel, Shafdan project (sample 24(B)): right valve outer (**A**) and dorsal (**B**) views. The pink hue is due to staining with eosin solution. Photograph courtesy S. Bartolini. Scale bar: 5 mm.

##### Chavania erythraea

Taxon classificationAnimaliaLucinidaLucinidae

(Issel, 1869)

DAAE4D2A-3661-5CFA-8B4D-02D84CDEED78

Figure 39

###### New records.

Israel • 1 spcm; Palmachim; 31.9292°N, 34.6405°E; depth 36.9 m; 29 May 2004; soft substrate; grab; NM project (station 19); size: L 3.2 mm, H 3.2 mm.

###### Remarks.

We report a juvenile but living individual of *Chavania erythraea*, a lucinid occurring in the Red Sea, the Persian (Arabian) Gulf and the Arabian Sea (Glover and Taylor 2001). This is the first record of this species from the Mediterranean Sea. Adults develop a commarginal lamellar sculpture.

**Figure 39.**
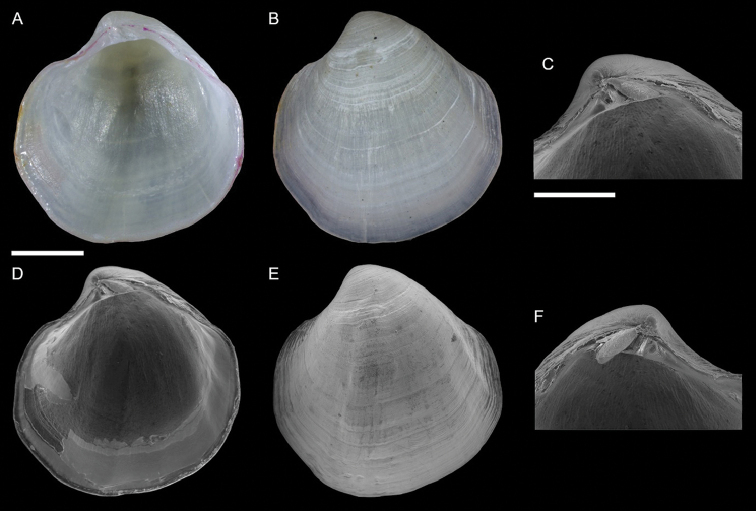
*Chavania erythraea* (Issel, 1869), Palmachim, Israel, NM project (station 19): right valve inner (**A, D**) and left valve outer (**B, E**) views, hinge of right (**C**) and left (**F**) valve. The pink hue is due to staining with eosin solution. Scale bars: 1 mm (**A, B, D, E**); 0.5 mm (**C, F**).

##### Rugalucina angela

Taxon classificationAnimaliaLucinidaLucinidae

(Melvill, 1899)

92ECED37-2D3F-57F2-9EE3-23A35CE76ED3

###### New records.

Israel • 1 spcm, 1 v; Ashqelon; 31.6868°N, 34.5516°E; depth 11 m; 31 Oct. 2018; offshore rocky reef; suction sampler; HELM project (samples S58_2M, S58_3M).

###### Remarks.

This species was first recorded as *Pillucina vietnamica* Zorina, 1978 (now in *Rugalucina* too) from the Mediterranean coast of Israel by Steger et al. (2018), who also illustrated it. A recent molecular phylogeny showed that *R. vietnamica* is distinct from *R. angela* and that the non-indigenous species in the Mediterranean belongs to this latter species, which occurs in the Red Sea and northwest Indian Ocean (Taylor and Glover 2019).

#### Family Galeommatidae Gray, 1840

##### Nudiscintilla cf. glabra

Taxon classificationAnimaliaGaleommatidaGaleommatidae

Lützen & Nielsen, 2005 (sensu Mifsud and Ovalis 2012)

E7AD6646-C860-5ED9-85B6-C70D67BFD519

Figure 40

###### New records.

**Figure 40.**
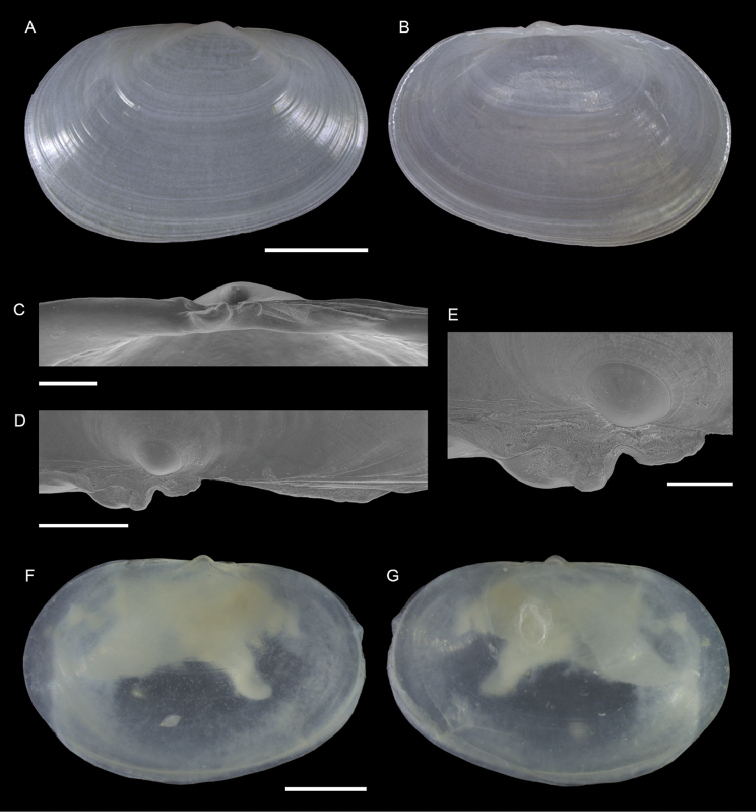
Nudiscintilla cf. glabra Lützen & Nielsen, 2005 (sensu Mifsud and Ovalis 2012) **A–E** Nahariyya, Israel, HELM project (sample D7): outer (**A**) and inner (**B**) views, detail of hinge (**C**) and hinge in dorsal view (**D, E**) of right valve **F, G** Palmachim, Israel, HELM project (sample H17): right (**F**) and left (**G**) valve outer views. Scale bars: 3 mm (**A, B**); 0.5 mm (**C, D**); 0.2 mm (**E**); 1 mm (**F, G**).

Israel • 1 v; Nahariyya, 200 m north of the entrance to the marina; 33.0149°N, 35.0890°E; depth 3–4 m; 6 Nov. 2018; pools with bioclastic sand in rocky bottom; snorkelled; J. Steger leg.; HELM project (sample D7); size: L 10.0 mm, H 6.4 mm (Figure 40A–E) • 1 spcm; Palmachim; 31.9285°N, 34.6947°E; depth 3 m; 7 Nov. 2018; attached to the lower valve of a living Lessepsian *Spondylus*; scuba diving; hand-picked; J. Steger & A. Ivkić leg.; HELM project (sample H17); size: L 4.2 mm, H 2.8 mm (Figure 40F, G).

###### Remarks.

This non-indigenous species has first been recorded in the Mediterranean Sea by Mifsud and Ovalis (2012) as Nudiscintilla cf. glabra Lützen and Nielsen, 2005, based on five living specimens collected at Yumurtalik, Adana (Turkey) in shallow water. Their tentative identification was primarily guided by the external morphology of the living animals, which had a smooth mantle surface. This feature is characteristic for the monotypic genus *Nudiscintilla* (hence the genus name), but unusual among scintilloid galeommatids in general. Although no observations on living individuals could be made by us, the shell morphology of our material well matches that of the specimen illustrated in Mifsud and Ovalis (2012: fig. 1), suggesting conspecificity. Our findings represent the first records of this species from Israel. However, the dentition of the right valve as seen in SEM images (Figure 40C–E) clearly differs from that described by Lützen and Nielsen (2005) for *Nudiscintilla*: the latter has a single cardinal tooth in each valve and no lateral teeth. However, the studied right valve – the hinge of the single live-collected specimen was not examined to avoid damage – bears what appears to be two cardinal teeth (Figure 40C) that are fused at their base (Figure 40D, E), as well as a ridge posterior to the internal ligament which most likely is a lateral tooth. This ridge seems to correspond to the left of the two swellings indicated by a pair of arrows on the right hand side of Mifsud and Ovalis (2012: fig. 1e), while the right swelling might correspond to a narrow ridge visible also on the dorsal margin of our valve. Mifsud and Ovalis (2012) interpreted these features as aberrant shell growth, however, the presence of such ridges also in our right valve (Figure 40D) speaks against this hypothesis. Furthermore, their living individuals had a small tentacle situated above the widely gaping anterior inhalant region (cf. Mifsud and Ovalis (2012: 8, fig. 2a), however, the illustration of *N. glabra* in Lützen and Nielsen (2005: 292, fig. 38a) shows a small tentacle in the posterior exhalant region of the reflected mantle. In the light of the poorly developed taxonomy and great species diversity of galeommatid bivalves in the Indo-Pacific, further observations on living specimens, thorough comparisons with the type material from Thailand and molecular analyses are required to definitely clarify the relationship of Mediterranean specimens with *N. glabra*.

##### Scintilla cf. violescens

Taxon classificationAnimaliaGaleommatidaGaleommatidae

Kuroda & Taki, 1961

9FC07CBE-A1EE-5586-97EA-8E9CE09B28FC

Figure 41

###### New records.

Israel • 1 spcm; Haifa; depth 15 m; May 1999; biogenic sediment; B.S. Galil leg.; size: L 6.9 mm, H 5.2 mm.

###### Remarks.

**Figure 41.**
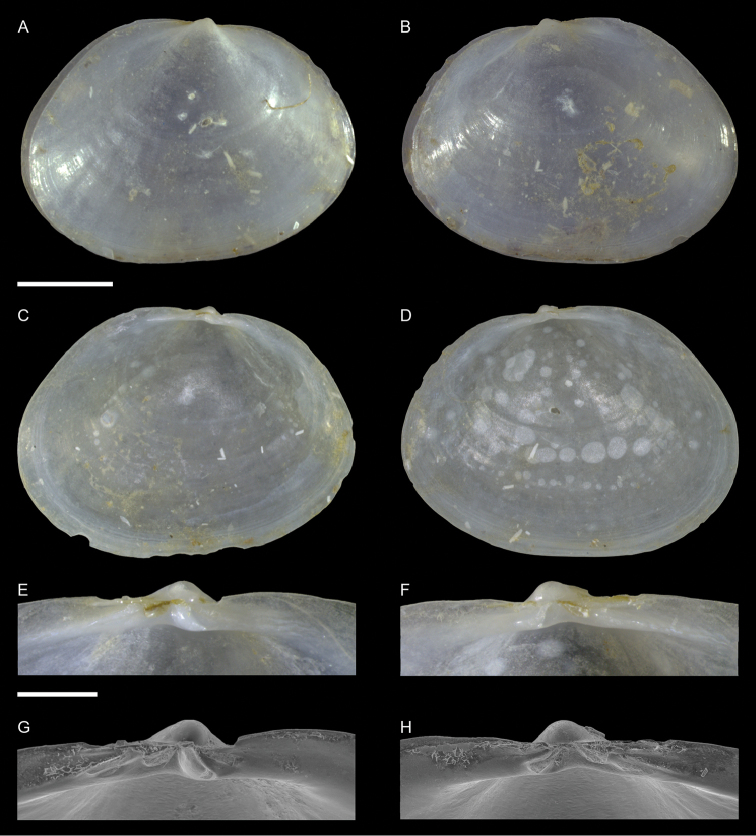
Scintilla cf. violescens Kuroda & Taki, 1961, Haifa, Israel: right (**A**) and left (**B**) valve outer views, left (**C**) and right (**D**) valve inner views, hinge of left (**E, G**) and right (**F, H**) valve. Scale bars: 2 mm (**A–D**); 0.5 mm (**E–H**).

The single shell found is trapezoid-oval (L:H ratio = 1.33), slightly higher posteriorly than anteriorly, translucid-white, and has a glossy external surface. The valves are narrowly gaping, more widely in their posterior part. The umbones are prosogyrate, pointed and submedian. The commarginal sculpture consists of fine growth lines that are slightly wavy posteriorly, as well as irregular growth marks. Flat radial ribs are present in the posterior part of the shell; they are visible, upon close examination, also on the inside of the valves in the form of shallow markings. The inner surface, particularly of the right valve, is spotted by blister-like markings (Figure 41C, D). The hinge of the right valve bears a single cardinal tooth, bent towards the anterior, and an elongated posterior lateral tooth. The left valve has two cardinals, but the anterior one is broken off (Figure 41G); a posterior lateral is present.

Lacking further material and observations on living individuals, which are of great diagnostic importance in galeommatids, we refrained from assigning a definitive specific name to our shell. However, the overall shape, hinge dentition and the presence of radial sculpture match well descriptions of *Scintilla violescens* Kuroda & Taki, 1961 (Arakawa 1961; Kuroda and Taki 1961), a species recorded from the intertidal and shallow subtidal of Thailand and Japan (Huber 2015). In contrast to our shell, however, Kuroda and Taki (1961) mention the presence of radial sculpture on the entire surface of the valves of their type material. While our Mediterranean shell is less elongated than the specimens of *S. violescens* illustrated by Okutani (2000), Lützen and Nielsen (2005), and Huber (2015), it is very similar in outline to the shell shown by Arakawa (1961: fig. 5B) (which was identified by T. Kuroda and I. Taki). *Scintilla violescens* appears to be variable also with respect to shell size and coloration: specimens from Thailand (maximum length = 10.5 mm, n = 12 spcms) all were considerably smaller than the > 15 mm-long Japanese type (Kuroda and Taki 1961) and had a whitish instead of pale violet color (Lützen and Nielsen 2005), like the Israeli shell. Considering this great plasticity in shell characters, and the differences in living animal morphology observed for Thai vs. Japanese specimens of *S. violescens* by Lützen and Nielsen (2005), the question arises whether more than one biological entity might be involved.

Irrespective of its unresolved specific affinity, the shell presented here clearly differs from all native Mediterranean galeommatids and thus cannot be confused; it can be easily distinguished from the non-indigneous Nudiscintilla cf. glabra (see above) by its less elongated shell, smaller L:H-ratio and, most notably, the presence of radial sculpture on the valves. Apart from the present shell, which was found in 1999 in Haifa Bay, we know of no other material.

#### Family Psammobiidae J. Fleming, 1828

##### Gari pallida

Taxon classificationAnimaliaCardiidaPsammobiidae

(Deshayes, 1855)

44C5DF60-A54A-54DA-9C48-D41427B3CDCE

###### New records.

Israel • 6 spcms; Ashqelon; 31.7002°N, 34.5498°E; depth 21 m; 18 Sep. 2016; sand; grab; HELM project (samples SG20_1F, SG20_3F, SG20_3M).

###### Remarks.

A single living individual of this species has been recently reported from Palmachim, along the southern Mediterranean coast of Israel, representing the first record from the Mediterranean Sea (Lubinevsky et al. 2018). We here confirm the establishment of this species by reporting six further living individuals, again from southern Israel.

#### Family Semelidae Stoliczka, 1870 (1825)

##### Ervilia scaliola

Taxon classificationAnimaliaCardiidaSemelidae

Issel, 1869

2B6A7F20-29BF-5D64-BFEA-9CDD57969DC5

Figure 42

###### New records.

Israel • 2 vv; Ashqelon; 31.7002°N, 34.5498°E; depth 21 m; 18 Sep. 2016; sand; grab; HELM project (sample SG20_2F) • 1 sh; Ashqelon; 31.6868°N, 34.5516°E; depth 11 m; 31 Oct. 2018; offshore rocky reef; suction sampler; HELM project (sample S58_3M); NHMW-MO-112930/LM/0176; size: L 3.1 mm, H 2.2 mm (illustrated shell).

###### Remarks.

**Figure 42.**
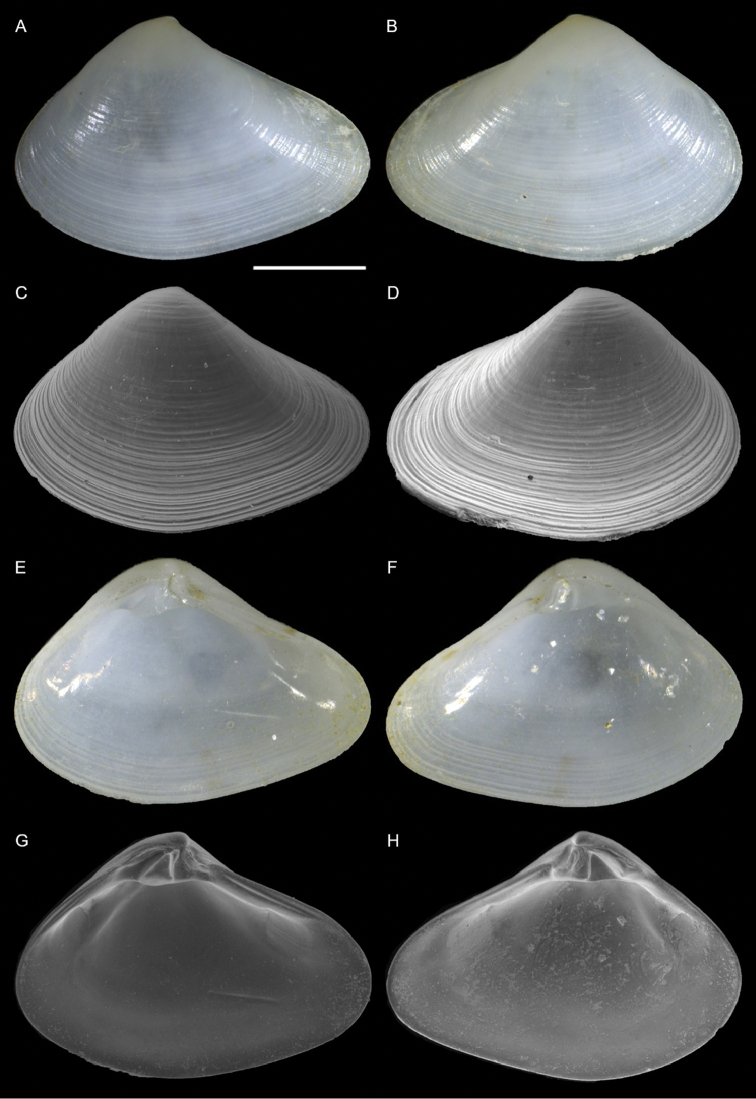
*Ervilia scaliola* Issel, 1869, NHMW-MO-112930/LM/0176, Ashqelon, Israel, HELM project (sample S58_3M): right (**A, C**) and left (**B, D**) valve outer views, left (**E, G**) and right (**F, H**) valve inner views. Scale bar: 1 mm.

*Ervilia scaliola* was first recorded from Turkey based on material collected in 2013 by Zenetos and Ovalis (2014) who correctly described the complex taxonomic status of this genus in the Indo-Pacific. We here record the species from Israel for the first time. The complete shell from Ashqelon (Figure 42) is very fresh, and thus probably originates from an extant population.

##### Iacra seychellarum

Taxon classificationAnimaliaCardiidaSemelidae

(A. Adams, 1856)

2FC16F6E-0635-5F8C-B084-C05A68760F25

Figure 43

###### New records.

GREECE • 3 vv; Kos Island; 2005–2010; A. Storm leg.; RMNH.MOL.342632; size: L 12.1 mm, H 9.8 mm.

###### Remarks.

We here report the finding of three beached valves of *Iacra seychellarum* in Kos, Greece. *Iacra seychellarum* can be readily distinguished from any Mediterranean semelid by its thick valves, large chondrophore and different sculpture in three zones: anterior with fine incised concentric lirations, median to posterior slope of fine incised oblique lines becoming strongly divaricate over the posterior slope, posterior area with closely spaced concentric incised lirations (Oliver 1992; Zuschin and Oliver 2003). It has been recorded from the northern Red Sea by Blatterer (2019) but has a broader distribution in the Indian Ocean (Oliver 1992). Because to our knowledge the finding has not been followed by others, we suggest that living individuals are needed to confirm the introduction and establishment of this species in the Mediterranean Sea.

**Figure 43.**
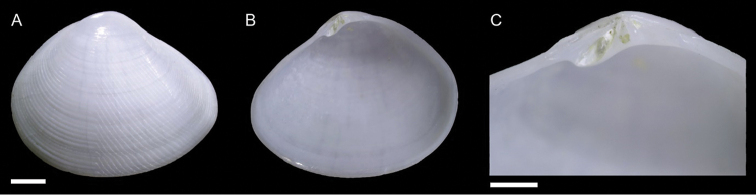
*Iacra seychellarum* (A. Adams, 1856), Kos Island, Greece, RMNH.MOL.342632: left valve outer (**A**) and inner (**B**) views, hinge (**C**). Photograph courtesy T. van Haaren. Scale bar: 2 mm (**A, B**); 1 mm (**C**).

##### Semelidae

Taxon classificationAnimaliaCardiidaSemelidae

sp.

3230B676-19B6-5AE2-9F92-0811AD290AC9

Figure 44

###### New records.

Israel • 1 spcm; Palmachim; 31.9737°N, 34.6767°E; depth 35.4 m; 24 May 2017; soft substrate; box-corer; Shafdan project (sample 29(A)); size: L 2.4 mm, H 2.0 mm.

###### Additional material examined.

*Abra alba* (W. Wood, 1802): Israel • 1 v; north of Atlit; 32.7422°N, 34.9181°E; depth 30 m; 20 Sep. 2016; sand; grab; HELM project (sample NG30_2M) • 2 vv; Ashqelon; 31.7101°N, 34.5406°E; depth 31 m; 18 Sep. 2016; sand; grab; HELM project (sample SG30_5M).

###### Remarks.

We were unable to identify this peculiar bivalve beyond family level, despite a thorough search in the literature on Mediterranean and Indo-Pacific mollusks. The shell of the single specimen found is roundly subtrigonal, fragile, with a rounded, steeply sloping anterior and a subacute to subtruncate posterior part; the ventral margin is slightly concave, the umbo submedian. The outer surface is smooth, glossy, and only sculptured by very fine growth lines. Although the small size and outline are reminiscent of certain galeommatoid genera such as *Bornia* Philippi, 1836, which is also represented in the Mediterranean Sea, the presence of both an external and an internal ligament, the latter situated on a well-developed resilifer, is typical for the family Semelidae (Oliver 1992; Beesley and Ross 1998; Huber 2010). The hinge of both valves bears two cardinals; the posterior cardinal of the left valve is becoming obsolete by encroachment of the internal ligament portion which also extends vertically beyond the hinge line. Two well-developed laterals are present in the right valve, while in the left valve, only a weak tooth-like ridge is present anteriorly, formed by the dorsal shell margin. Due to the extremely smooth and glossy interior of the valves that renders hardly visible even the adductor muscle scars, it remains unclear whether a deep pallial sinus, another feature typical of semelids, is present in the studied specimen.

*Lonoa katoi* Habe, 1976, a semelid from Japan, shares with our species the small size and irregular outline with an often concave ventral margin (related to its attached lifestyle); however, the outer shell surface of *L. katoi* bears rough lamellae and fine radial threads (Habe 1976; Okutani 2017), while that of the Israeli specimen is almost smooth. The most similar confamilial species from the Red Sea probably is *Abra aegyptiaca* Oliver and Zuschin, 2000, however, it differs from the specimen described here in shell shape, sculpture, the prosogyrate umbo, and features of the hinge such as the shape of the anterior lateral tooth of the right valve. Similar-sized juveniles of Mediterranean *Abra* spp., including *A. alba*, the most common species on the shallow Israeli shelf, have a hinge morphology comparable to our specimen, but differ in their outline and by having more protruding umbos (Scaperrotta et al. 2013). *Abra tenuis* is most similar in shape, and a teratological specimen might approach the outline of our shell; however, such a specimen would still differ from Semelidae sp. by the presence of commarginal lines on the early dissoconch (Scaperrotta et al. 2013; Oliver et al. 2020).

Until the finding of further specimens, it remains open whether the present individual is a juvenile or an adult of a small-sized species. Considering that only a single specimen was found so far, the lack of known native Mediterranean species with a similar morphology, and the geographical proximity of the Israeli coast to the Suez Canal, we suspect that this species might be another Indo-Pacific taxon introduced to the southeastern Mediterranean.

**Figure 44.**
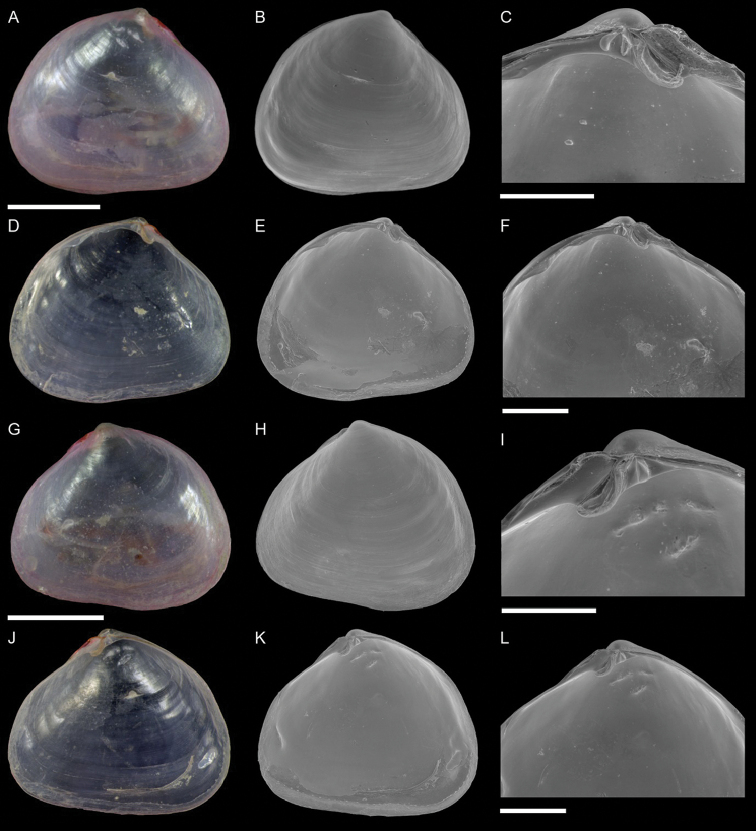
Semelidae sp., Palmachim, Israel, Shafdan project (sample 29(A)): left valve outer view (**A, B**), right valve inner view (**D, E**), hinge (**C, F**) and outer view (**G, H**), left valve inner view (**J–K**) and hinge (**I, L**). The pink hue is due to staining with eosin solution. Scale bars: 1 mm (**A, B, D, E, G, H, J, K**); 0.5 mm (**F, L**); 0.3 mm (**C, I**).

#### Family Veneridae Rafinesque, 1815

##### Clementia papyracea

Taxon classificationAnimaliaVeneridaVeneridae

(Gmelin, 1791)

666AA834-81EA-5E3E-89EC-D2EFA87239EE

###### New records.

Israel • 1 spcm; Haifa Bay; 32.8211°N, 35.0196°E; depth 11 m; 2 Aug. 2015; soft substrate; grab; NM project (station HM27).

###### Remarks.

*Clementia papyracea* has been recorded in the Mediterranean Sea since 1937, but findings of living individuals are very scarce and limited to samples collected in 1968 in El Arish, Egypt (Barash and Danin 1973), in 1975 in Haifa, Israel (Barash and Danin 1977) and in 2012 in Ashqelon, Israel (Crocetta et al. 2016). We here report a further living juvenile individual.

#### Family Corbulidae Lamarck, 1818

##### Corbula erythraeensis

Taxon classificationAnimaliaMyidaCorbulidae

H. Adams, 1871

BBF4259F-A993-502A-B04A-032DB92C6F9D

Figure 45A–K

###### New records.

**Figure 45.**
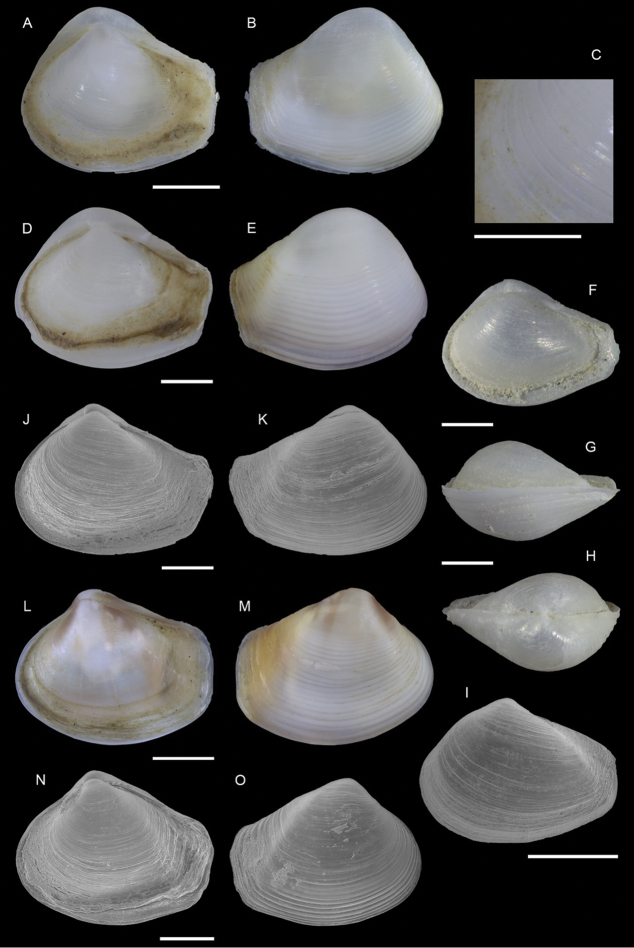
Comparison between *Corbula erythraeensis* H. Adams, 1871 and *Corbula gibba* (Olivi, 1792). **A–E***Corbula erythraeensis*, Ashqelon, Israel, HELM project (sample SG40_8M): **A–C** Specimen 1: left (**A**) and right (**B**) valve outer views, sculpture of the anterior left valve (**C**) **D, E** Specimen 2: left (**D**) and right (**E**) valve outer views. **F–H***Corbula erythraeensis*, Bay of Safaga, Egypt, NHMW-MO-106107: left valve outer (**F**), ventral (**G**) and dorsal (**H**) views. **I***Corbula erythraeensis*, same collecting data as for preceding, NHMW-MO-106100: left valve outer view **J, K***Corbula erythraeensis*, Soreq desalination plant, Israel, Soreq project: left (**J**) and right (**K**) valve outer views **L, M***Corbula gibba*, same collecting data as for **A–E**: left (**L**) and right (**M**) valve outer views **N, O***Corbula gibba*, same collecting data as for **J–K**: left (**N**) and right (**O**) valve outer views. Scale bars: 1 mm (**A, B, D, E, F–I, L, M**); 0.5 mm (**C, J, K, N, O**).

Israel • 1 spcm; Ashqelon; 31.7002°N, 34.5498°E; depth 21 m; 18 Sep. 2016; sand; grab; HELM project (sample SG20_4F) • 3 spcms; Ashqelon; 31.7487°N, 34.4960°E; depth 41 m; 27 Apr. 2017; sandy mud; grab; HELM project (sample SG40_8M); sizes: L 2.9 mm, H 2.5 mm (illustrated specimen 1, Figure 45A–C); L 3.8 mm, H 3.1 mm (illustrated specimen 2, Figure 45D, E) • 2 spcms; Soreq desalination plant; 31.94°N, 34.69°E; depth 17–22 m; 10 May 2009; soft substrate; grab; Soreq project; size: L 1.9 mm, H 1.5 mm (Figure 45J, K).

###### Additional material examined.

*Corbula erythraeensis*: Egypt • 1 sh; Bay of Safaga; 26.8142°N, 33.9653°E; depth 39 m; 31 Oct. 1994; mud; scuba diving; M. Zuschin leg.; ref. Oliver and Zuschin (2000), sample 94/4/b; NHMW-MO-106107; size: L 3.5 mm, H 2.5 mm (illustrated shell, Figure 45F–H) • 1 v; same collecting data as for preceding; NHMW-MO-106100; size: L: 2.3 mm, H: 1.6 mm (illustrated valve, Figure 45I).

*Corbula gibba* (Olivi, 1792): Israel • 271 spcms; Ashqelon; 31.7492°N, 34.4964°E; depth 41 m; 27 Apr. 2017; sandy mud; grab; HELM project (sample SG40_8M) • 300 spcms, 11 shs, 10 vv (5 right, 5 left); Soreq desalination plant; 31.94°N, 34.69°E; depth 17–22 m; 10 May 2009; soft substrate; grab; Soreq project.

###### Remarks.

*Corbula erythraeensis* is widespread in the northern Red Sea where it has been recorded from the gulfs of Suez (MacAndrew 1870; Oliver 1992) and Aqaba at Eilat (Edelman-Furstenberg and Faershtein 2010), as well as the northern Bay of Safaga (Egypt) (Zuschin and Oliver 2003); outside the Red Sea, its distribution ranges eastward to Pakistan (Huber 2010). Here we report the first findings of this species from the Mediterranean. *Corbula erythraeensis* was found sympatrically with the common native Mediterranean *Corbula gibba* but was always present in very low numbers. No empty shells have been found so far. While being similar in appearance to the morphologically variable *C. gibba*, *C. erythraeensis* has a convex anterior dorsal margin of the right valve (usually concave in *C. gibba*, particularly in smaller specimens), a more inflated umbonal region, a regular concentric sculpture on the left valve (Oliver 1992), and its color always is whitish-yellowish (*C. gibba* frequently has a rosy pattern). Juvenile individuals are more wedge-shaped than those of *C. gibba*.

## Discussion

### Massive reporting follows intensive fieldwork and broad cooperation

We have covered 52 species, reporting the finding of 23 new Lessepsian mollusks, nine additional species that, upon final identification, may turn out to be further new Lessepsian species, nine new records for Eastern Mediterranean countries and new data for eleven already recognized non-indigenous species. Such a massive report is derived from three characteristics of this study which translate into recommendations for an effective approach to non-indigenous species detection and monitoring. First, the intensive sampling effort and the effective sampling techniques of the “Historical ecology of Lessepsian migration” (HELM) project and of the IOLR monitoring programs. The HELM project in particular targeted also hard substrates, poorly explored at this taxonomic resolution in the Eastern Mediterranean, by suction sampling. This technique has repeatedly proved to be a very effective method on compact (e.g., coral rubble, pebbles (Bouchet et al. 2002; Linnane et al. 2003; Ringvold et al. 2015; Evans et al. 2018)), seagrass (Bonfitto et al. 1998; Albano and Sabelli 2012; Albano and Stockinger 2019) or hard substrates (Templado et al. 2010) and has enabled here the collection of vast amounts of living micromollusks and their shells. Indeed, 26 out of the 52 (50%) species treated here and 17 out of 32 (53%) new and potentially new NIS came from suction samples on hard substrates. The IOLR surveys covered soft substrates along the whole Israeli Mediterranean coastline with a dense station network and multi-seasonal sampling that led to the detection of 16 out of 52 (31%) treated species, notwithstanding several findings had already been published (e.g., Bogi and Galil 2013b, Aartsen et al. 2015; Lubinevsky et al. 2018). Second, the use of fine mesh sizes. The HELM samples were sieved with a 0.5 mm mesh and those of the IOLR monitoring programs were sieved with either a 0.5 mm or even a 250 μm mesh. Such small sizes enabled retaining the large majority of invertebrates, including mollusks, even those with very small and elongated shells, such as many Pyramidellidae. However, this approach requires an enormous effort when picking and sorting the samples and the availability of high-level taxonomic expertise, since most small-sized species belong to taxonomically challenging groups or represent juvenile individuals. Third, and importantly, cooperation among institutions and individuals. Despite new records of non-indigenous species are often scattered into short papers in the literature and may become difficult to trace in the long term, recent efforts have demonstrated the value of cooperation to build up large datasets (e.g., Katsanevakis et al. 2020). It is also important to highlight the role that citizen scientists had in the detection of the new non-indigenous species treated here by contributing to sample sorting, species identification and their taxonomic study.

### The challenge of recognizing and identifying tropical non-indigenous species

Taxonomic uncertainty is recognized as a major impediment to the reliable inventorying of non-indigenous species (McGeoch et al. 2012; Marchini et al. 2015; Katsanevakis and Moustakas 2018). Species whose identification is uncertain or whose taxonomic status is unresolved were suggested to be excluded from inventories (Marchini et al. 2015). Taxonomic uncertainty may also imply an uncertain non-indigenous status: a species morphologically distinct from the native species pool can either be a yet undescribed native species or a newly introduced species (which may be undescribed too). If the new species belongs to a clade not occurring in the sampled range, then the attribution of the non-indigenous status is well supported. Still, only the finding of clearly conspecific individuals from the source pool would provide final evidence of the non-indigenous status. We exemplified these cases with two new taxa described here: *Coriophora lessepsiana* Albano, Bakker & Sabelli, sp. nov. clearly belongs to a tropical clade of Triphoridae and the availability of material from the Red Sea enabled the unambiguous attribution of the species to the Red Sea pool (hence the species name *lessepsiana*). *Joculator problematicus* Albano & Steger, sp. nov. belongs to a genus absent from the Mediterranean Sea. Despite multiple similar species have been recorded in the Indo-Pacific province, we have not been able to find conspecific Red Sea material (hence the species name *problematicus*, to highlight the uncertainty in attributing the non-indigenous status when solid taxonomic and faunistic knowledge is lacking).

A more complex case is represented by species that have few diagnostic characters, hampering the unequivocal attribution to a tropical clade (e.g., within Eulimidae and Pyramidellidae). We here propose to exploit the properties of death assemblages to deliver a solid hypothesis of non-indigenous status. Death assemblages, the taxonomically identifiable, dead or discarded organic remains encountered in a landscape or seabed such as molluscan shells, accumulate species richness over time (Kidwell 2013). This property implies that death assemblages are good archives of local species diversity and that species which have long occurred in a study site (like native ones) are likely to be found in a death assemblage even if they are too rare to be regularly detected in living censuses. Indeed, the inclusion of empty shells in field surveys increased the estimations of occupancy and detectability of native land snails (Albano et al. 2015). In contrast, a species which has recently established a population in a new area would be expected to be poorly detectable in the death assemblage because not enough time has elapsed to contribute a significant number of skeletal parts to it. Consequently, newly reported non-indigenous species found alive but not, or very rarely, in the death assemblage may represent newly introduced species. This approach can be applied when living and death assemblages are sampled simultaneously with quantitative methods. The main limitation of this approach is that it is applicable only to organisms with hard skeletal parts like foraminiferans, ostracods and fishes (e.g. Agiadi and Albano 2020).

With biological invasions constituting a major element of global change, there is understandable concern on the accuracy of non-indigenous species inventories (Marchini et al. 2015; Katsanevakis and Moustakas 2018). However, also the risk of underestimating the magnitude of biological invasions must be considered. In this respect, the Lessepsian invasion is a special case. The salinity reduction of the Bitter Lakes (Galil 2006), the containment by dams of the annual Nile flood which used to modulate salinity off its delta (Rilov and Galil 2009), and the multiple enlargements of the Suez Canal (Galil et al. 2015) have certainly enhanced connectivity between the Mediterranean Sea and the Indo-Pacific province during the 20^th^ century. Additionally, in the most recent decades, rising seawater temperatures in the Mediterranean Sea (Ozer et al. 2017) make it increasingly suitable for the establishment of inherently thermophilic Red Sea species. These factors combined render very likely that an increasing number of Red Sea species manages to cross the Canal and to settle in the Mediterranean Sea. The proper understanding of this phenomenon and of its drivers requires a timely and detailed census of assemblages as well as a broad toolkit to overcome the limitations of the taxonomic challenges associated with tropical species.

## Authors’ contributions

PGA and JS conceived the study. PGA, JS, CB, MB, TGH, MFH, HL, MM, and MS contributed to fieldwork, sample sorting and data acquisition. PGA, JS, PAJB, CB, PILF, and BS identified the specimens and contributed to the taxonomic discussion. PGA, JS, and TGH contributed to the discussion. PGA, JS, PAJB, and MA prepared the figures. PGA and JS wrote the first draft of the manuscript, which then received contributions by all co-authors.

## Funding

This research has been conducted in the context of the “Historical ecology of Lessepsian migration” project funded by the Austrian Science Fund (FWF) P28983-B29 (PI: P.G. Albano). Sampling in Crete was supported by the “Kurzfristige wissenschaftliche Auslandsstipendien” program of the University of Vienna to M. Stockinger, the non-profit organization Mare Mundi, and the diving school Dive2gether. Sampling in northeastern Cyprus was conducted in the framework of the project “Classification of coastal habitats of Northern Cyprus according to EUNIS protocol” (PI: F. Huseynoglu). The Faculty of Earth Sciences of the University of Vienna funded a citizen science project conducted in October 2019. MB was supported by an Ernst Mach fellowship of the OeAD (Österreichischer Austauschdienst).

## Supplementary Material

XML Treatment for Conradia eutornisca

XML Treatment for Dikoleps micalii

XML Treatment for Skeneidae

XML Treatment for Fossarus

XML Treatment for Cycloscala hyalina

XML Treatment for Eunaticina papilla

XML Treatment for Coriophora lessepsiana

XML Treatment for Opimaphora blattereri

XML Treatment for Triphora

XML Treatment for Viriola cf. bayani

XML Treatment for Cerithiopsis sp. aff. pulvis

XML Treatment for Cerithiopsis

XML Treatment for Joculator problematicus

XML Treatment for Elachisina

XML Treatment for Iravadia aff. elongata

XML Treatment for Vitrinella aff. Vitrinella

XML Treatment for Hypermastus

XML Treatment for Hemiliostraca clandestina

XML Treatment for Melanella orientalis

XML Treatment for Parvioris aff. dilecta

XML Treatment for Sticteulima

XML Treatment for Vitreolina cf. philippi

XML Treatment for Conus fumigatus

XML Treatment for Henrya

XML Treatment for Odostomia cf. dalli

XML Treatment for Odostomia

XML Treatment for Odostomia

XML Treatment for Oscilla virginiae

XML Treatment for Parthenina cossmanni

XML Treatment for Parthenina typica

XML Treatment for Pyrgulina craticulata

XML Treatment for Pyrgulina nana

XML Treatment for Turbonilla funiculata

XML Treatment for Cylichna collyra

XML Treatment for Mnestia girardi

XML Treatment for Atys angustatus

XML Treatment for Arcuatula perfragilis

XML Treatment for Lioberus ligneus

XML Treatment for Musculus coenobitus

XML Treatment for Musculus aff. viridulus

XML Treatment for Isognomon aff. australica

XML Treatment for Pegophysema cf. philippiana

XML Treatment for Chavania erythraea

XML Treatment for Rugalucina angela

XML Treatment for Nudiscintilla cf. glabra

XML Treatment for Scintilla cf. violescens

XML Treatment for Gari pallida

XML Treatment for Ervilia scaliola

XML Treatment for Iacra seychellarum

XML Treatment for Semelidae

XML Treatment for Clementia papyracea

XML Treatment for Corbula erythraeensis

## References

[B1] Aartsen JJ van (1981) European marine Mollusca: notes on less well-known species. II. The genus *Cima* Chaster, 1896. Basteria 45: 117–119.

[B2] Aartsen JJ van Goud J (2006) Indo-Pacific migrants into the Mediterranean. 3. *Atys angustatus* Smith, 1872 (Gastropoda, Opisthobranchia). Basteria 70: 29–31.

[B3] Aartsen JJ van Barash A Carrozza F (1989) Addition to the knowledge of the Mediterranean Mollusca of Israel and Sinai. Bollettino Malacologico 25: 63–76.

[B4] Aartsen JJ van Galil B Bogi C (2015) Two alien venerid bivalves from the Eastern Mediterranean. Marine Biodiversity Records 8: e83. 10.1017/S1755267215000573

[B5] Adams A (1856) Descriptions of thirty four new species of bivalve Mollusca (*Leda*, *Nucula*, and *Pythina*) from the Cumingian collection. Proceedings of the Zoological Society of London 24: 47–53.

[B6] Adams A (1861) On some new genera and species of Mollusca from the North of China and Japan. Annals and Magazine of Natural History ser. 3, 8: 239–246. 10.1080/00222936108697411

[B7] Adams H (1871) Descriptions of twenty-six new species of shells collected by Robert M’Andrew, Esq., in the Red Sea. Proceedings of the Zoological Society of London 1870: 788–793.

[B8] Adams J (1797) The specific characters of some minute shells discovered on the coast of Pembrokeshire, with an account of a new marine animal. Transactions of the Linnean Society of London 3: 64–69. 10.1111/j.1096-3642.1797.tb00559.x

[B9] Agamennone F Micali P Siragusa F (2020a) *Melanella orientalis* n. sp. (Gastropoda: Eulimidae) from the Eastern Mediterranean. Bollettino Malacologico 56: 172–175.

[B10] Agamennone F Sbrana C Nardi N Siragusa F Germanà A (2020b) *Dikoleps micalii* n. sp. (Gastropoda: Skeneidae) from the Eastern Aegean Sea. Bollettino Malacologico 56: 91–95.

[B11] Agiadi K Albano PG (2020) Holocene fish assemblages provide baseline data for the rapidly changing eastern Mediterranean. The Holocene 30: 1438–1450. 10.1177/0959683620932969

[B12] Albano PG Sabelli B (2012) The molluscan assemblages inhabiting the leaves and rhizomes of a deep water *Posidonia oceanica* settlement in the central Tyrrhenian Sea. Scientia Marina 76: 721–732. 10.3989/scimar.03396.02C

[B13] Albano PG Bakker PAJ (2016) Annotated catalogue of the types of Triphoridae (Mollusca, Gastropoda) in the Museum für Naturkunde, Berlin, with lectotype designations. Zoosystematics and Evolution 92: 33–78. 10.3897/zse.92.5936

[B14] Albano PG Stockinger M (2019) The rhizome layer of *Posidonia oceanica*: an important habitat for Mediterranean brachiopods. Marine Biodiversity 49: 2467–2472. 10.1007/s12526-019-00968-6

[B15] Albano PG Sabelli B Bouchet P (2011) The challenge of small and rare species in marine biodiversity surveys: microgastropod diversity in a complex tropical coastal environment. Biodiversity and Conservation 20: 3223–3237. 10.1007/s10531-011-0117-x

[B16] Albano PG Bakker PAJ Sabelli B (2019) Annotated catalogue of the types of Triphoridae (Mollusca, Gastropoda) in the Natural History Museum of the United Kingdom, London. Zoosystematics and Evolution 95: 161–308. 10.3897/zse.95.32803

[B17] Albano PG Strazzari G D’Occhio P Succetti F (2015) Field estimates of detectability and site occupancy show that northern Italy forest molluscs are spatially rare and poorly detectable. Italian Journal of Zoology 82: 592–608. 10.1080/11250003.2015.1040084

[B18] Albano PG Bakker PAJ Janssen R Eschner A (2017) An illustrated catalogue of Rudolf Sturany’s type specimens in the Naturhistorisches Museum Wien, Austria (NHMW): Red Sea gastropods. Zoosystematics and Evolution 93: 45–94. 10.3897/zse.93.10039 PMC6225989 30416602

[B19] Albano PG Gallmetzer I Haselmair A Tomašových A Stachowitsch M Zuschin M (2018) Historical ecology of a biological invasion: the interplay of eutrophication and pollution determines time lags in establishment and detection. Biological Invasions 20: 1417–1430. 10.1007/s10530-017-1634-7 29805296 PMC5959955

[B20] Albano PG Azzarone M Amati B Bogi C Sabelli B Rilov G (2020) Low diversity or poorly explored? Mesophotic molluscs highlight undersampling in the Eastern Mediterranean. Biodiversity and Conservation 29: 4059–4072. 10.1007/s10531-020-02063-w 33191987 PMC7658090

[B21] Albano PG Steger J Bošnjak M Dunne B Guifarro Z Turapova E Hua Q Kaufman DS Rilov G Zuschin M (2021) Native biodiversity collapse in the Eastern Mediterranean. Proceedings of the Royal Society B: Biological Sciences 288. 10.1098/rspb.2020.2469 PMC7892420 33402072

[B22] Ammar I (2018) New record of alien species of gastropods in Syrian coast. Damascus University Journal of Basic Science 34: 95–122.

[B23] Angelidis A Polyzoulis G (2018) New distributional records of four Indo-Pacific species from Astypalaia Island, South Aegean Sea, Greece. Xenophora Taxonomy 21: 3–10.

[B24] Appolloni M Smriglio C Amati B Lugliè L Nofroni I Tringali LP Mariottini P Oliverio M (2018) Catalogue of the primary types of marine molluscan taxa described by Tommaso Allery Di Maria, Marquis of Monterosato, deposited in the Museo Civico di Zoologia, Roma. Zootaxa 4477: 1–138. 10.11646/zootaxa.4477.1.1 30313335

[B25] Arakawa KY (1961) A note on the animal of *Scintilla violescens* collected in Genkai Sea. Venus 21: 143–146.

[B26] Audouin V (1826) Explication sommaire des planches de Mollusques de l’Egypte et de la Syrie publiées par J.C. Savigny. In: Description de l’Egypte ou recueil des observations et des recherches qui ont été faites en Egypte pendant l’expédition de l’armée française, publié par les ordres de sa majesté l’empereur Napoléon le grand. Histoire Naturelle, Animaux invertébrés. Imprimerie impériale, Paris, 7–56.

[B27] Barash A Danin Z (1971) Opisthobranchia (Mollusca) from the Mediterranean waters of Israel. Israel Journal of Zoology 20: 151–200.

[B28] Barash A Danin Z (1973) Contributions to the knowledge of Suez Canal migration. The Indo-Pacific species of Mollusca in the Mediterranean and notes on a collection from the Suez Canal. Israel Journal of Zoology 21: 301–374.

[B29] Barash A Danin Z (1977) Additions to the knowledge of Indo-Pacific Mollusca in the Mediterranean. Conchiglie 13: 85–116.

[B30] Bariche M Fricke R (2020) The marine ichthyofauna of Lebanon: an annotated checklist, history, biogeography, and conservation status. Zootaxa 4775: 1–157. 10.11646/zootaxa.4775.1.1 33055597

[B31] Bartsch P (1947) A monograph of the west Atlantic mollusks of the family Aclididae. Smithsonian Miscellaneous Collections 106: 1–29.

[B32] Beesley PL Ross GJB (Eds) (1998) Mollusca: the southern synthesis. Fauna of Australia. Volume 5. CSIRO Publishing, Melbourne, part A XVI + 563 pp, part B VIII + 565 pp.

[B33] Belmaker J Brokovich E China V Golani D Kiflawi M (2009) Estimating the rate of biological introductions: Lessepsian fishes in the Mediterranean. Ecology 90: 1134–1141. 10.1890/07-1904.1 19449707

[B34] Beu AG Alloway BV Pillans BJ Naish TR Westgate JA (2004) Marine Mollusca of oxygen isotope stages of the last 2 million years in New Zealand. Part 1: Revised generic positions and recognition of warm‐water and cool‐water migrants. Journal of the Royal Society of New Zealand 34: 111–265. 10.1080/03014223.2004.9517766

[B35] Blatterer H (2019) Mollusca of the Dahab region (Gulf of Aqaba, Red Sea). Denisia 43: 1–480.

[B36] Bodilis P Arceo H Francour P (2011) Further evidence of the establishment of *Fistularia commersonii* (Osteichthyes: Fistulariidae) in the north-western Mediterranean Sea. Marine Biodiversity Records 4: E18. 10.1017/S1755267211000194

[B37] Bogi C Galil BS (1999) Nuovi ritrovamenti di immigranti lessepsiani lungo le coste israeliane. La Conchiglia 31: 29–32.

[B38] Bogi C Galil BS (2006) Nuovi ritrovamenti lungo le coste israeliane. Notiziario S.I.M. 24: 16–18.

[B39] Bogi C Galil BS (2013a) *Cylichna villersii*, an Erythraean cephalaspideid snail (Mollusca: Gastropoda: Opisthobranchia) in the eastern Mediterranean. Marine Biodiversity Records 6: e92. 10.1017/S1755267213000687

[B40] Bogi C Galil BS (2013b) *Finella pupoides* Adams A., 1860 (Gastropoda, Scaliolidae) – a population explosion underway, Mediterranean Sea. BioInvasions Records 2: 43–45. 10.3391/bir.2013.2.1.07

[B41] Bogi C Karhan SU Yokes MB (2012) Oscilla galilae, a new species of Pyramidellidae (Mollusca, Gastropoda, Heterobranchia) from the Eastern Mediterranean. Iberus 30: 1–6.

[B42] Bonfitto A Fellagara I Gillone G (1998) Sampling techniques and structure of the malacofauna associated to the rhizome zone in *Posidonia oceanica* (L.) Delile. Bollettino Malacologico 33: 83–88.

[B43] Bouchet P (1989) A review of poecilogony in gastropods. Journal of Molluscan Studies 55: 67–78. 10.1093/mollus/55.1.67

[B44] Bouchet P Danrigal F (1982) Napoleon’s Egyptian campaign (1798–1801) and the Savigny collection of shells. The Nautilus 96: 9–24.

[B45] Bouchet P Warén A (1993) Revision of the northeast Atlantic bathyal and abyssal Mesogastropoda. Bollettino Malacologico Suppl. 3: 579–840. 10.5962/bhl.title.140732

[B46] Bouchet P Lozouet P Maestrati P Heros V (2002) Assessing the magnitude of species richness in tropical marine environments: exceptionally high numbers of molluscs at a New Caledonia site. Biological Journal of the Linnean Society 75: 421–436. 10.1046/j.1095-8312.2002.00052.x

[B47] Bouchet P Rocroi J-P Bieler R Carter JG Coan EV (2010) Nomenclator of bivalve families with a classification of bivalve families. Malacologia 52: 1–184. 10.4002/040.052.0201

[B48] Bouchet P Rocroi J-P Hausdorf B Kaim A Kano Y Nützel A Parkhaev P Schrödl M Strong EE (2017) Revised classification, nomenclator and typification of gastropod and monoplacophoran families. Malacologia 61: 1–526. 10.4002/040.061.0201

[B49] Bruguière JG (1792) Encyclopédie méthodique ou par ordre de matières. Histoire naturelle des vers, volume 1. Pancoucke, Paris, 345–757. [Livraison 48] 10.5962/bhl.title.49857

[B50] Brusina S (1869) Gasteropodes nouveaux de l’Adriatique. Journal de Conchyliologie 17: 230–249.

[B51] Buzzurro G Cecalupo A (2005) Descrizione di una nuova specie di Cerithiopsidae per le coste turche. Bollettino Malacologico 40: 77–79.

[B52] Buzzurro G Cecalupo A (2006) I molluschi lessepsiani di Taşucu (Turchia sud-orientale): descrizione di *Parviturbo dibellai* n. sp. (Gastropoda: Trochoidea: Skeneidae). Bollettino Malacologico 42: 27–32.

[B53] Cantraine FJ (1835) Diagnoses ou descriptiones succinctes de quelques espèces nouvelles de mollusques. Bulletin de l’Académie Royale des Sciences et Belles-lettres de Bruxelles 2: 380–401.

[B54] Carpenter PP (1856) Description of new species and varieties of Calyptraeidae, Trochidae, and Pyramidellidae, principally in the collection of Hugh Cuming, Esq. Proceedings of the Zoological Society of London 24: 166–171.

[B55] Cecalupo A Quadri P (1994) Contributo alla conoscenza malacologica per il nord dell’Isola di Cipro (parte I). Bollettino Malacologico 30: 5–16.

[B56] Cecalupo A Villari A (1997) *Dizoniopsis micalii*. Una nuova specie per il Mediterraneo (Mesogastropoda: Cerithiopsidae). Bollettino Malacologico 32: 41–44.

[B57] Cecalupo A Perugia I (2011) The family Cerithiopsidae H. Adams & A. Adams, 1853 in the Central Philippines (Caenogastropoda: Triphoroidea). Quaderni della Civica Stazione Idrobiologica di Milano 30: 5–262.

[B58] Cecalupo A Perugia I (2013) The Cerithiopsidae (Caenogastropoda: Triphoroidea) of Espiritu Santo - Vanuatu (South Pacific Ocean). Published by the Authors, 253 pp.

[B59] Cecalupo A Perugia I (2014a) Cerithiopsidae and Newtoniellidae (Gastropoda: Triphoroidea Gray) from French Polynesia area (South Pacific Ocean). Novapex 15: 1–22.

[B60] Cecalupo A Perugia I (2014b) The Cerithiopsidae (Caenogastropoda: Triphoroidea) of South Madagascar (Indian Ocean). Bollettino Malacologico 50: 75–126.

[B61] Cecalupo A Perugia I (2016) Report on some Cerithiopsidae from Indo-Pacific area (Caenogastropoda: Triphoroidea). Bollettino Malacologico 52: 98–109.

[B62] Cecalupo A Perugia I (2017a) A new species of Cerithiopsidae (Gastropoda: Triphoroidea) from the East China Sea. Bollettino Malacologico 53: 30–32.

[B63] Cecalupo A Perugia I (2017b) Cerithiopsidae and Newtoniellidae (Gastropoda: Triphoroidea) from New Caledonia, Western Pacific. Visaya Suppl. 7: 5–175.

[B64] Cecalupo A Perugia I (2018) New species of Cerithiopsidae (Gastropoda: Triphoroidea) from Papua New Guinea (Pacific Ocean). Visaya Suppl. 11: 4–187.

[B65] Cecalupo A Perugia I (2019a) New species of Cerithiopsidae and Newtoniellidae from Okinawa (Japan - Pacific Ocean). Visaya Suppl. 12: 4–84.

[B66] Cecalupo A Perugia I (2019b) New species of Cerithiopsidae the Philippines and Samoa Islands (Caenogastropoda: Triphoroidea). Visaya 5: 19–34.

[B67] Cecalupo A Perugia I (2019c) Report from Bangka Pulau (Indonesia): Cerithiopsidae. Bollettino Malacologico 55: 11–22.

[B68] Chartosia N Anastasiadis D Bazairi H Crocetta F Deidun A Despalatović M Martino VD Dimitriou N Dragičević B Dulčić J Durucan F Hasbek D Ketsilis-Rinis V Kleitou P Lipej L Macali A Marchini A Ousselam M Piraino S Stancanelli B Theodosiou M Tiralongo F Todorova V Trkov D Yapici S (2018) New Mediterranean Biodiversity Records (July 2018). Mediterranean Marine Science 19: 398–415. 10.12681/mms.18099

[B69] Chester C Agosti D Sautter G Catapano T Martens K Gérard I Bénichou L (2019) EJT editorial standard for the semantic enhancement of specimen data in taxonomy literature. European Journal of Taxonomy 586: 1–22. 10.5852/ejt.2019.586

[B70] Çinar ME Bilecenoglu M Oztürk B Katagan T Yokes MB Aysel V Dagli E Acik S Ozcan T Erdogan H (2011) An updated review of alien species on the coasts of Turkey. Mediterranean Marine Science 12: 257–315. 10.12681/mms.34

[B71] Crocetta F Bitar G Zibrowius H Oliverio M (2013) Biogeographical homogeneity in the eastern Mediterranean Sea. II. Temporal variation in Lebanese bivalve biota. Aquatic Biology 19: 75–84. 10.3354/ab00521

[B72] Crocetta F Tringali LP Mienis HK Zenetos A (2016) *Clementia papyracea* (Gmelin, 1791) (Mollusca: Bivalvia: Veneridae): its established status in the Mediterranean Sea and the first record from Greece. Cahiers de Biologie Marine 57: 271–275.

[B73] Crocetta F Bitar G Zibrowius H Oliverio M (2020) Increase in knowledge of the marine gastropod fauna of Lebanon since the 19^th^ century. Bulletin of Marine Science 96: 22. 10.5343/bms.2019.0012

[B74] Crocetta F Gofas S Salas C Tringali LP Zenetos A (2017) Local ecological knowledge versus published literature: a review of non-indigenous Mollusca in Greek marine waters. Aquatic Invasions 12: 415–434. 10.3391/ai.2017.12.4.01

[B75] Crooks JA (2005) Lag times and exotic species: The ecology and management of biological invasions in slow-motion. Ecoscience 12: 316–329. 10.2980/i1195-6860-12-3-316.1

[B76] Daniel B Piro S Charbonnel E Francour P Letourneur Y (2009) Lessepsian rabbitfish *Siganus luridus* reached the French Mediterranean coasts. Cybium 33: 163–164.

[B77] Dekker H Orlin Z (2000) Checklist of Red Sea Mollusca. Spirula 47: 1–46.

[B78] Deshayes GP (1855) Descriptions of new shells from the collection of H. Cuming. Esq. Proceedings of the Zoological Society of London 22: 317–371. 10.1111/j.1469-7998.1854.tb07284.x

[B79] Dunker WR (1857) Mytilacea nova collectione Cumingianae. Proceedings of the Zoological Society of London 24: 358–366.

[B80] Dunker WR (1882) Index molluscorum maris Japonici. Conscriptus et tabulis iconum XVI illustratus. Cassellis Cattorum. Sumptibus Theodori Fischer, 390 pp. 10.5962/bhl.title.10277

[B81] Edelman-Furstenberg Y Faershtein G (2010) Molluscan fauna of the Gulf of Elat: indicators of ecological change. Jerusalem, 132 pp.

[B82] Ellingson RA Krug PJ (2006) Evolution of poecilogony from planktotrophy: cryptic speciation, phylogeography, and larval development in the gastropod genus *Alderia*. Evolution 60: 2293–2310. 10.1111/j.0014-3820.2006.tb01866.x 17236422

[B83] Evans J Attrill MJ Borg JA Cotton PA Schembri PJ (2018) Hidden in plain sight: species richness and habitat characterisation of sublittoral pebble beds. Marine Biology 165: 35. 10.1007/s00227-018-3292-4

[B84] Folin AGL de (1868) Quelques points de la côte septentrionale de Java (Tome 1, 1er Partie, Chapitre 14). In: Folin AGL de Périer JPL (Eds) , Les Fonds de la Mer.Étude internationale sur les particularités nouvelles des régions sous-marines. Savy, Libraire-Editeur, Paris, 54–108.

[B85] Folin AGL de (1879) Mers de Chine. Mollusques. Les Fonds de la Mer. Paris [1878], 263–267.

[B86] Forbes E Hanley SC (1850) A history of British Mollusca and their shells. Vol. 2. van Voorst., London, 481–557.

[B87] Galil BS (2006) The marine caravan – The Suez Canal and the Erythrean invasion. In: Gollasch S Galil BS Cohen AN (Eds) Bridging divides.Maritime canals as invasion corridors. Monographiae Biologicae. Springer, Dordrecht, 207–301. 10.1007/978-1-4020-5047-3_6

[B88] Galil BS (2009) Taking stock: inventory of alien species in the Mediterranean Sea. Biological Invasions 11: 359–372. 10.1007/s10530-008-9253-y

[B89] Galil BS Boero F Campbell ML Carlton JT Cook E Fraschetti S Gollasch S Hewitt CL Jelmert A Macpherson E Marchini A McKenzie C Minchin D Occhipinti-Ambrogi A Ojaveer H Olenin S Piraino S Ruiz GM (2015) ‘Double trouble’: the expansion of the Suez Canal and marine bioinva 10.1007/s10530-014-0778-y sions in the Mediterranean Sea. Biological Invasions 17: 973–976.

[B90] Giannuzzi-Savelli R Pusateri F Micali P Nofroni I Bartolini S (2014) Atlante delle conchiglie marine del Mediterraneo Vol. 5 (Heterobranchia). Edizioni Danaus, Palermo, 111 + 191 pp.

[B91] Giunchi L Micali P Tisselli M (2001) Report of two lessepsian migrants from Turkey. La Conchiglia 33: 47–48. 10.2307/25304703

[B92] Glover EA Taylor JD (2001) Systematic revision of Australian and Indo-Pacific Lucinidae (Mollusca: Bivalvia): *Pillucina*, *Wallucina* and descriptions of two new genera and four new species. Records of the Australian Museum 53: 263–292. 10.3853/j.0067-1975.53.2001.1349

[B93] Gmelin JF (1791) Vermes. In: Gmelin JF (Ed.) Caroli a Linnaei Systema Naturae per Regna Tria Naturae.G.E. Beer, Lipsiae [Leipzig], 3021–3910.

[B94] Gofas S (1990) Le genre *Gibberula* (Marginellidae) en Mediterranee. Lavori della Società Italiana di Malacologia 23: 113–140.

[B95] Gofas S (1992) Le genre *Granulina* (Marginellidae) en Mediterranee et dans l’Atlantique Oriental. Bollettino Malacologico 28: 1–26.

[B96] Gofas S Moreno D Salas C (2011) Moluscos marinos de Andalucía. Volumen II - Clase Gastropoda (Heterobranchia), clase Bivalvia, clase Scaphopoda, clase Cephalopoda, glosario e índices. Universidad de Málaga, Servicio de Publicaciones e Intercambio Científico, Málaga, 343–798.

[B97] Gould AA (1861) Description of new shells collected by the United States North Pacific Exploring Expedition. Proceedings of the Boston Society of Natural History: 7: 385–389 [January 1861], 401–409 [February 1861]; 8: 14–32 [March 1861], 33–40 [April 1861].

[B98] Guy‐Haim T Hyams‐Kaphzan O Yeruham E Almogi‐Labin A Carlton JT (2017) A novel marine bioinvasion vector: Ichthyochory, live passage through fish. Limnology and Oceanography Letters 2: 81–90. 10.1002/lol2.10039

[B99] Habe T (1976) Eight new bivalves from Japan. Venus 35: 37–46.

[B100] Hedley C (1903) Studies on Australian Mollusca. Part. 7. Proceedings of the Linnean Society of New South Wales 27: 596–619.

[B101] Hedley C (1909) Mollusca from the Hope Islands, north Queensland. Proceedings of the Linnean Society of New South Wales 34: 420–466.

[B102] Hervier J (1898) Diagnoses d’espèces nouvelles de *Triforis*, provenant de l’archipel de la Nouvelle-Calédonie (suite). Journal de Conchyliologie 45: 249–266.

[B103] Higo S Callomon P Goto Y (2001) Catalogue and bibliography of the marine shell-bearing Mollusca of Japan. Gastropoda. Bivalvia. Polyplacophora. Scaphopoda. Type figures. Elle Scientific Publications, Osaka, 196 pp.

[B104] Hinds RB (1843) Descriptions of new shells from the collection of Captain Belcher, R.N., C.B. The Annals and Magazine of Natural History 11: 16–21. 10.1080/03745484309445254

[B105] Hoagland KE Robertson R (1988) An assessment of poecilogony in marine invertebrates: phenomenon or fantasy? The Biological Bulletin 174: 109–125. 10.2307/1541778

[B106] Høisæter T (2009) . Distribution of marine, benthic, shell bearing gastropods along the Norwegian coast. Fauna norvegica, 28, 5-106. 10.5324/fn.v28i0.563

[B107] Hornung A Mermod G (1924) Mollusques de la Mer Rouge recueillis par A. Issel faisant partie des collections du Musée Civique d’Histoire Naturelle de Gênes. Première partie, Pyramidellides. Annali del Museo Civico di Storia Naturale “G. Doria” 51: 283–311.

[B108] Hornung A Mermod G (1925) Mollusques de la Mer Rouge recueillis par A. Issel faisant partie des collections du Musée Civique d’Histoire Naturelle de Gênes. Deuxième partie, Pyramidellides (fin) - Rissoinides. Annali del Museo Civico di Storia Naturale “G. Doria” 52: 20–33.

[B109] Hornung A Mermod G (1928) Mollusques de la Mer Rouge recueillis par A. Issel faisant partie des collections du Musée Civique d’Histoire Naturelle de Gênes. Quatrième partie, Rissoidés. Annali del Museo Civico di Storia Naturale “G. Doria” 52: 363–372.

[B110] Huber M (2010) Compendium of bivalves. ConchBooks, Hackenheim, 901 pp.

[B111] Huber M (2015) Compendium of bivalves 2. ConchBooks, Harxheim, 907 pp.

[B112] Issel A (1869) Malacologia del Mar Rosso. Ricerche zoologiche e paleontologiche. Biblioteca Malacologica, xi–387 pp.

[B113] Ivkić A Steger J Galil BS Albano PG (2019) The potential of large rafting objects to spread Lessepsian invaders: the case of a detached buoy. Biological Invasions 21: 1887–1893. 10.1007/s10530-019-01972-4 31148942 PMC6510832

[B114] Janssen R Zuschin M Baal C (2011) Gastropods and their habitats from the northern Red Sea (Egypt: Safaga) Part 2: Caenogastropoda: Sorbeoconcha and Littorinimorpha. Annalen des Naturhistorischen Museums in Wien 113: 373–509.

[B115] Jeffreys JG (1858) Gleanings in British Conchology. Annals and Magazine of Natural History: (3)1: 39–48, pl. 2; (3)2: 117–133, 5. 10.1080/00222935808696993

[B116] Johnson RI (1964) The Recent Mollusca of Augustus Addison Gould. Illustrations of the types described by Gould with a bibliography and catalog of his species. United States National Museum Bulletin 239: 1–182. 10.5479/si.03629236.239

[B117] Jousseaume FP (1884) Monographie des Triforidae. Bulletins de la Société Malacologique de France 1: 217–270.

[B118] Jousseaume FP (1898) Triphoridae de la Mer Rouge. Bulletin de la Société Philomathique ser. 8, 9: 71–77.

[B119] Katsanevakis S Moustakas A (2018) Uncertainty in Marine Invasion Science. Frontiers in Marine Science 5: 38. 10.3389/fmars.2018.00038

[B120] Katsanevakis S Tsiamis K Ioannou G Michailidis N Zenetos A (2009) Inventory of alien marine species of Cyprus (2009). Mediterranean Marine Science 10: 109–134. 10.12681/mms.113

[B121] Katsanevakis S Poursanidis D Hoffman R Rizgalla J Bat-Sheva Rothman S Levitt-Barmats Y Hadjioannou L Trkov D Garmendia JM Rizzo M Bartolo AG Bariche M Tomas F Kleitou P Schembri PJ Kletou D Tiralongo D Pergent C Pergent G (2020) Unpublished Mediterranean records of marine alien and cryptogenic species. BioInvasions Records 9: 165–182. 10.3391/bir.2020.9.2.01

[B122] Kay EA (1979) Hawaiian marine shells. Reef and shore fauna of Hawaii. Section 4: Mollusca. 64xviii + 1–653.

[B123] Kidwell SM (2013) Time-averaging and fidelity of modern death assemblages: building a taphonomic foundation for conservation palaeobiology. Palaeontology 56: 487–522. 10.1111/pala.12042

[B124] Kosuge S (1962) Descriptions of 10 new species and 1 new subspecies of the family Triphoridae (Mollusca) from Shiono-misaki, Kii Peninsula, Central Japan with a list of hitherto known species. Bulletin of the National Science Museum (Tokyo) 6: 78–89.

[B125] Krug PJ (1998) Poecilogony in an estuarine opisthobranch: planktotrophy, lecithotrophy, and mixed clutches in a population of the ascoglossan *Alderia modesta*. Marine Biology 132: 483–494. 10.1007/s002270050414

[B126] Kuroda T Taki I (1961) On a new species of *Scintilla* (Galeommatidae) from Japan. Venus 21: 141–142.

[B127] Laseron CF (1956) The family Cerithiopsidae (Mollusca) from the Solanderian and Dampierian zoogeographical provinces. Australian Journal of Marine and Freshwater Research 7: 151–182. 10.1071/MF9560151

[B128] Laseron CF (1958) The family Triphoridae (Mollusca) from northern Australia; also Triphoridae from Christmas Island (Indian Ocean). Australian Journal of Marine and Freshwater Research 9: 569–658. 10.1071/MF9580569

[B129] Laseron CF (1959) The family Pyramidellidae from Northern Australia. Australian Journal of Marine and Freshwater Research 10: 177–267. 10.1071/MF9590177

[B130] van der Linden J Eikenboom JCA (1992) On the taxonomy of the Recent species of the genus *Chrysallida* Carpenter from Europe, the Canary Islands and the Azores (Gastropoda, Pyramidellidae). Basteria 56: 3–63.

[B131] Linnaeus C (1758) Systema Naturae per regna tria naturae, secundum classes, ordines, genera, species, cum characteribus, differentiis, synonymis, locis. Editio decima, reformata [10^th^ revised edition]. Laurentius Salvius, Holmiae, 824 pp.

[B132] Linnaeus C (1767) Systema naturae per regna tria naturae: secundum classes, ordines, genera, species, cum characteribus, differentiis, synonymis, locis. Ed. 12. 1., Regnum Animale. 1 & 2. Laurentii Salvii, Holmiae, 1327 pp 10.5962/bhl.title.156772

[B133] Linnane A Ball B Munday B Browne R Mercer JP (2003) Faunal description of an Irish cobble site using airlift suction sampling. Biology and Environment: Proceedings of the Royal Irish Academy 103B: 41–48. 10.3318/BIOE.2003.103.1.41

[B134] Lipej L Acevedo I Akel EHK Anastasopoulou A Angelidis A Azzurro E Castriota L Çelik M Cilenti L Crocetta F Deidun A Dogrammatzi A Falautano M Fernández-Álvarez FÁ Gennaio R Insacco G Katsanevakis S Langeneck J Lombardo BM Mancinelli G Mytilineou C Papa L Pitacco V Pontes M Poursanidis D Prato E Rizkalla SI Rodríguez-Flores PC Stamouli C Tempesti J Tiralongo F Tirnetta S Tsirintanis K Turan C Yaglioglu D Zaminos G Zava B (2017) New Mediterranean biodiversity records (March 2017). Mediterranean Marine Science 18: 179–201. 10.12681/mms.2068

[B135] Lubinevsky H Galil B Bogi C (2018) First record of *Gari pallida* (Deshayes, 1855) (Mollusca: Bivalvia: Psammobiidae) in the Mediterranean Sea. BioInvasions Records 7: 415–419. 10.3391/bir.2018.7.4.10

[B136] Lützen J Nielsen C (2005) Galeommatid bivalves from Phuket, Thailand. Zoological Journal of the Linnean Society 144: 261–308. 10.1111/j.1096-3642.2005.00168.x

[B137] MacAndrew R (1870) Report on the testaceous Mollusca obtained during a dredging-excursion in the Gulf of Suez in the months of February and March 1869. Annals and Magazine of Natural History (Fourth Series) 6: 429–450. 10.1080/00222937008696289

[B138] Marchini A Galil BS Occhipinti-Ambrogi A (2015) Recommendations on standardizing lists of marine alien species: Lessons from the Mediterranean Sea. Marine Pollution Bulletin 101: 267–273. 10.1016/j.marpolbul.2015.09.054 26471066

[B139] Marshall BA (1978) Cerithiopsidae (Mollusca: Gastropoda) of New Zealand, and a provisional classification of the family. New Zealand Journal of Zoology 5: 47–120. 10.1080/03014223.1978.10423744

[B140] Mazziotti C Agamennone F Micali P Tisselli M (2005) Descrizione di *Turbonilla flaianoi* n. sp. per il Mare Adriatico. Bollettino Malacologico 41: 79–84.

[B141] McDonald KA Collin R Lesoway MP (2014) Poecilogony in the caenogastropod *Calyptraea lichen* (Mollusca: Gastropoda). Invertebrate Biology 133: 213–220. 10.1111/ivb.12057

[B142] McGeoch MA Spear D Kleynhans EJ Marais E (2012) Uncertainty in invasive alien species listing. Ecological Applications 22: 959–971. 10.1890/11-1252.1 22645824

[B143] Melvill JC (1896) Descriptions of new species of minute marine shells from Bombay. Proceedings of the Malacological Society of London 2: 108–116.

[B144] Melvill JC (1899) Notes on the Mollusca of the Arabian Sea, Persian Gulf, and Gulf of Oman, mostly dredged by Mr. F. W. Townsend, with descriptions of twenty-seven species. Annals and Magazine of Natural History ser. 7, 4: 81–101. 10.1080/00222939908678167

[B145] Melvill JC (1904) Descriptions of twelve new species and one variety of marine Gastropoda from the Persian Gulf, Gulf of Oman and Arabian sea, collected by Mr. F. W. Townsend, 1902–1904. Journal of Malacology 11: 79–85.

[B146] Melvill JC (1906a) A revision of the species of Cyclostrematidae and Liotiidae occurring in the Persian Gulf and North Arabian Sea. Proceedings of the Malacological Society of London 7: 20–30. 10.1093/oxfordjournals.mollus.a066121

[B147] Melvill JC (1906b) Descriptions of thirty-one Gastropoda and one scaphopod from the Persian Gulf and Gulf of Oman, dredged by Mr. F.W. Townsend, 1902–1904. Proceedings of the Malacological Society of London 7: 69–80. 10.1093/oxfordjournals.mollus.a066148

[B148] Melvill JC (1912) Descriptions of thirty-three new species of Gastropoda from the Persian Gulf, Gulf of Oman, and North Arabian Sea. Proceedings of the Malacological Society of London 10: 240–254.

[B149] Melvill JC (1918) Descriptions of thirty-four species of marine Mollusca from the Persian Gulf, Gulf of Oman and Arabian Sea, collected by Mr. F. W. Townsend. Annals and Magazine of Natural History Ser. 9, 1: 137–158. 10.1080/00222931808562296

[B150] Micali P Bogi C Galil BS (2016) On the occurrence of *Atys angustatus* E. A. Smith, 1872 and *Atys macandrewii* E. A. Smith, 1872 (Cephalaspidea: Haminoeidae) in the Mediterranen Sea. Iberus 34: 1–5.

[B151] Micali P Siragusa F Agamennone F Germanà A Sbrana C (2017) Karpathos Island (Greece) and its Indo-Pacific alien species, Part 1. Bollettino Malacologico 53: 40–49.

[B152] Mienis HK (1976) *Ventomnestia girardi* (Audouin, 1827) from the Mediterranean. Conchiglie 12: 209–210.

[B153] Mienis HK (2019) A first record of *Anodontia philippiana* from the Mediterranean Sea off Israel. Triton 38: 4–5.

[B154] Mifsud C Ovalis P (2012) A galeommatid bivalve new to the Mediterranean Sea. Triton 26: 6–8.

[B155] Mifsud C Ovalis P (2019) Two new species of *Sticteulima* Laseron, 1955 (Gastropoda: Eulimidae) from Turkey, eastern Mediterranean. Bollettino Malacologico 55: 68–71.

[B156] Milaschewitsch KO (1916) Mollyuski Russkikh Morey. Tom 1. Mollyuski Chernago i Azovskago Morey. Imperatorskaya Akademiya Nauk, Zoologicheskiy Muzey, Petrograd, 312 pp.

[B157] MolluscaBase (2020) MolluscaBase. http://www.molluscabase.org [March 7, 2020]

[B158] Montagu G (1803) Testacea Britannica or natural history of British shells, marine, land, and fresh-water, including the most minute: Systematically arranged and embellished with figures. J. White, London, Vol. 1: xxxvii + 291 pp.; Vol. 2: 293–606 + 16 pls. 10.5962/bhl.title.33927

[B159] Montagu G (1808) Supplement to Testacea Britannica with Additional Plates. Woolmer, Exeter, v + 183 pp.

[B160] Monterosato TA di (1874) Recherches conchyliologiques, effectuées au Cap Santo Vito, en Sicile. Journal de Conchyliologie 22: 243–282.

[B161] Monterosato TA di (1877) Notizie sulle conchiglie della rada di Civitavecchia. Annali del Museo Civico di Storia Naturale “G. Doria” 9: 407–428.

[B162] Monterosato TA di (1878) Note sur quelques coquilles draguées dans les eaux de Palerme. Journal de Conchyliologie 26: 143–160.

[B163] Occhipinti-Ambrogi A Marchini A Cantone G Castelli A Chimenz C Cormaci M Froglia C Furnari G Gambi MC Giaccone G Giangrande A Gravili C Mastrototaro F Mazziotti C Orsi-Relini L Piraino S (2011) Alien species along the Italian coasts: an overview. Biological Invasions 13: 215–237. 10.1007/s10530-010-9803-y

[B164] Okutani T (2000) Marine mollusks in Japan. Tokai University Press, Tokyo, 1173 pp.

[B165] Okutani T (2017) Marine mollusks in Japan. 2^nd^ edition. Tokai University Press, Tokyo, 1375 pp.

[B166] Oliver PG (1992) Bivalved seashells of the Red Sea. Verlag Christa Hemmen, 330 pp.

[B167] Oliver PG (2015) Old shell collection casts new light on an alien species. The dark false mussel (*Mytilopsis leucophaeata*) may have been in Britain as early as 1800! Journal of Conchology 42: 1.

[B168] Oliver PG Zuschin M (2000) Addition to the bivalve fauna of the Red Sea with descriptions of new species of Limopsidae, Tellinidae and Semelidae. Journal of Conchology 37: 17–37.

[B169] Oliver PG Holmes AM Killeen IJ Turner JA (2020) Marine bivalve shells of the British Isles. https://naturalhistory.museumwales.ac.uk/BritishBivalves/home.php

[B170] Oliverio M Buzzurro G Villa R (1994) A new Eulimid Gastropod from the Eastern Mediterranean sea (Caenogastropoda, Ptenoglossa). Bollettino Malacologico 30: 211–215.

[B171] Olivi G (1792) Zoologia Adriatica, ossia catalogo ragionato degli animali del golfo e delle lagune di Venezia. G. Remondini e fl., Bassano, 334 pp 10.5962/bhl.title.60887

[B172] Ounifi-Ben Amor K Rifi М Ghanem R Draeif I Zaouali J Ben Souissi J (2015) Update of alien fauna and new records from Tunisian marine waters. Mediterranean Marine Science 17: 124. 10.12681/mms.1371

[B173] Ozer T Gertman I Kress N Silverman J Herut B (2017) Interannual thermohaline (1979–2014) and nutrient (2002–2014) dynamics in the Levantine surface and intermediate water masses, SE Mediterranean Sea. Global and Planetary Change 151: 60–67. 10.1016/j.gloplacha.2016.04.001

[B174] Özturk B Aartsen JJ van (2006) Indo-Pacific migrants into the Mediterranean. 5 *Chrysallida micronana* nom. nov. for *Chrysallida nana* (Hornung and Mermod, 1924) (Gastropoda:Pyramidellidae). Aquatic Invasions 1: 241–244. 10.3391/ai.2006.1.4.7

[B175] Öztürk B Bitlis Bakir B (2013) *Eunaticina papilla* Gmelin, 1791) (Naticidae, Gastropoda) a new alien in the eastern Mediterranean. Triton 28: 7–8.

[B176] Öztürk B Bitlis B Filiz ME (2011) The genus *Chrysallida* Carpenter, 1856 on the Turkish coasts (Gastropoda: Heterostropha). Zoology in the Middle East 54: 53–78. 10.1080/09397140.2011.10648880

[B177] Pease WH (1861) Descriptions of forty-seven new species of shells from the Sandwich Islands, in the collection of Hugh Cuming. Proceedings of the Zoological Society of London 28: 431–438.

[B178] Pease WH (1871) Remarks on the genus *Triphoris* (Desh.), with descriptions of new species. Proceedings of the Zoological Society of London 1870: 773–777.

[B179] Peñas A Rolán E (2010) 26 Deep water Pyramidelloidea from the Central and South Pacific: *Turbonilla* and related genera. Mémoires du Muséum national d’Histoire naturelle, Paris, 436 pp.

[B180] Peñas A Rolán E (2017) Deep water Pyramidelloidea from the Central and South Pacific: the tribe Chrysallidini.

[B181] Peñas A Rolán E Sabelli B (2020) The family Pyramidellidae in the Red Sea. I. The tribe Chrysallidini. Iberus 39: 1–93.

[B182] Pennant T (1777) British Zoology, vol. IV. Crustacea Mollusca Testacea. London, i–viii, 1–154 pp.

[B183] Philippi RA (1836) Enumeratio molluscorum Siciliae cum viventium tum in tellure tertiaria fossilium, quae in itinere suo observavit. Schropp, Berlin, Vol. 1. I-XIV, 1–303. [Tab. XIII-XXVIII pp] 10.5962/bhl.title.100735

[B184] Ponder WF Fukuda H Hallan A (2014) A review of the family Clenchiellidae (Mollusca: Caenogastropoda: Truncatelloidea). Zootaxa 3872: 101–153. 10.11646/zootaxa.3872.2.1 25544076

[B185] Por FD (1978) Lessepsian migration: the influx of Red Sea biota into the Mediterranean by way of the Suez Canal. Springer-Verlag, Berlin Heidelberg, VIII + 228 pp.

[B186] Prkić J Buzzurro G (2007) A new species of *Cerithiopsis* (GastropodaCerithiopsidae) from Croatian coasts. Triton 15: 1–4.

[B187] Raitsos DE Beaugrand G Georgopoulos D Zenetos A Pancucci-Papadopoulou AM Theocharis A Papathanassiou E (2010) Global climate change amplifies the entry of tropical species into the eastern Mediterranean Sea. Limnology and Oceanography 55: 1478–1484. 10.4319/lo.2010.55.4.1478

[B188] Rayneval AG de van den Hecke EBG Ponzi G (1854) Catalogue des fossiles du Monte Mario (près Rome), recueillis par M. le Cte de Rayneval, Mgr Vanden Hecke et M. le professeur Ponzi. impr. Beau jeune, Versailles, 17 pp 10.5962/bhl.title.112311

[B189] Reeve LA (1850) Monograph of the genus *Lucina*. In: Conchologia Iconica. L. Reeve & Co., London, pl. 1–11 and unpaginated text.

[B190] Reeve LA (1858a) Monograph of the genus *Modiola*. In: Conchologia Iconica. L. Reeve & Co., London, pl. 1–11 and unpaginated text.

[B191] Reeve LA (1858b) Monograph of the genus *Perna*. In: Conchologia Iconica, or illustrations of the shells of molluscous animals. Vol. 11. L. Reeve & Co., London, 8.

[B192] Rilov G (2016) Multi-species collapses at the warm edge of a warming sea. Scientific Reports 6: 36897. 10.1038/srep36897 PMC5113072 27853237

[B193] Rilov G Galil B (2009) Marine bioinvasions in the Mediterranean Sea – History, distribution and ecology. In: Rilov G Crooks JA (Eds) Biological Invasions in Marine Ecosystems.Ecological Studies. Springer Berlin Heidelberg, Berlin, Heidelberg, 549–575. 10.1007/978-3-540-79236-9_31

[B194] Ringvold H Grytnes J-A van der Meeren GI (2015) Diver-operated suction sampling in Norwegian cobble grounds: technique and associated fauna. Crustaceana 88: 184–202. 10.1163/15685403-00003406

[B195] Risso A (1826) Histoire naturelle des principales productions de l’Europe Méridionale et particulièrement de celles des environs de Nice et des Alpes Maritimes. Tome quatrième. F.-G. Levrault, Paris, vii–439 pp. 10.5962/bhl.title.58984

[B196] Robba E Di Geronimo I Chaimanee N Negri MP Sanfilippo R (2004) Holocene and Recent shallow soft-bottom mollusks from the northern Gulf of Thailand area: Scaphopoda, Gastropoda, additions to Bivalvia. La Conchiglia 35: 5–288.

[B197] Röckel D (1986) Sensational find in the Mediterranean. La Conchiglia 18: 12.

[B198] Rolán E Gofas S (2003) The family Elachisinidae (Mollusca, Rissooidea) in the temperate and tropical Atlantic. Iberus 21: 67–90.

[B199] Rubio F Rolán E Fernández-Garcés R (2015) Revision of the genera *Parviturbo* and *Pseudorbis* (Gastropoda, Skeneidae). Iberus 33: 167–259.

[B200] Russini V Giannuzzi-Savelli R Pusateri F Prkic J Fassio G Modica MV Oliverio M (2020) Candidate cases of poecilogony in Neogastropoda: implications for the systematics of the genus *Raphitoma* Bellardi, 1847. Invertebrate Systematics 34(3): 293–318.

[B201] Saurin E (1959) Pyramidellidae de Nhatrang (Vietnam). Annales de la Faculté des Sciences (Saigon) 1959: 223–283.

[B202] Savigny JC (1817) Description de l’Egypte ou recueil des observations et des recherches qui ont été faites en Egypte pendant l’expédition de l’armée française, publié par ordre du Gouvernement. Histoire Naturelle, Planches, Tome Deuxième. Imprimerie Royale, Paris, Coquilles, pl. 1–14.

[B203] Scaperrotta M Bartolini S Bogi C (2012) Accrescimenti. Stadi di accrescimento dei molluschi marini del Mediterraneo. Volume IV. L’Informatore Piceno, 184 pp.

[B204] Scaperrotta M Bartolini S Bogi C (2013) Accrescimenti. Stadi di accrescimento dei molluschi marini del Mediterraneo. Volume V. L’Informatore Piceno, 192 pp.

[B205] Scaperrotta M Bartolini S Bogi C (2019) Accrescimenti. Stadi di accrescimento dei molluschi marini del Mediterraneo. Volume X. L’Informatore Piceno, 212 pp.

[B206] Schechter HC Mienis HK (2020) A first record of *Eunaticina linneana* from the Mediterranean Coast of Israel (Gastropoda, Naticidae). Triton 40: 4–5.

[B207] Sigovini M Keppel E Tagliapietra D (2016) Open Nomenclature in the biodiversity era. Methods in Ecology and Evolution 7: 1217–1225. 10.1111/2041-210X.12594

[B208] Smith EA (1872) Remarks on several species of Bullidae, with descriptions of some hitherto undescribed forms, and of a new species of *Planaxis*. Annals and Magazine of Natural History 4: 344–355. 10.1080/00222937208696599

[B209] Smith EA (1899) Descriptions of new species of South African marine shells. Journal of Conchology 9: 247–252.

[B210] Solow AR Costello CJ (2004) Estimating the Rate of Species Introductions from the Discovery Record. Ecology 85: 1822–1825. 10.1890/03-3102

[B211] Sowerby GBI (1844) Monograph of the genus *Scalaria*. In: Sowerby GBI (Ed.), Thesaurus Conchyliorum. Privately published, London, 83bis–108bis.

[B212] Stamouli C Akel EHK Azzurro E Bakiu R Bas AA Bitar G Boyaci Y Cakalli M Corsini-Foka M Crocetta F Dragičević B Dulčić J Durucan F El Z Erguden D Filiz H Giardina F Giovos I Gönülal O Hemida F Kassar A Kondylatos G Macali A Mancini E Ovalis P De M Pavičić M Rabaoui L Rizkalla SI Tiralongo F Turan C Vrdoljak D Yapici S Zenetos A (2017) New Mediterranean biodiversity records (December 2017). Mediterranean Marine Science 18: 534–556.

[B213] Steger J Stockinger M Ivkić A Galil B Albano PG (2018) New records of non-indigenous molluscs from the eastern Mediterranean Sea. BioInvasions Records 7: 245–257. 10.3391/bir.2018.7.3.05 30406051 PMC6218014

[B214] Sturany R (1903) Expeditionen S.M. Schiff “Pola” in das Rothe Meer, nördliche und südliche Hälfte, 1895/96 – 1897/98. Zoologische Ergebnisse XXIII. Gastropoden des Rothen Meeres. Denkschriften der Kaiserlichen Akademie der Wissenschaften, Mathematisch-Naturwissenschaftliche Classe 74: 210–283. [Preprint 1–75 pp.]

[B215] Taylor JD Glover EA (2019) Unloved, paraphyletic or misplaced: new genera and species of small to minute lucinid bivalves and their relationships (Bivalvia, Lucinidae). ZooKeys 899: 109–140. 10.3897/zookeys.899.47070 31875090 PMC6926428

[B216] Templado J Paulay G Gittenberger A Meyer C (2010) Sampling the marine realm. In: Eymann J Degreef J Häuser C Monje JC Samyn Y VandenSpiegel D (Eds) Manual on field recording techniques and protocols for all taxa biodiversity inventories.ABC Taxa, 273–302.

[B217] Tryon GW (1886) Manual of conchology, structural and systematic, with illustrations of the species. (1)8: Naticidae, Calyptraeidae, Turritellidae, Vermetidae, Caecidae, Eulimidae, Turbonillidae, Pyramidellidae. Published by the Author, Philadelphia, 461 pp.

[B218] Vaillant L (1865) Recherches sur la faune malacologique de la baie de Suez. Journal de Conchyliologie 13: 97–127.

[B219] Valdés Á (2008) Deep-sea “cephalaspidean” heterobranchs (Gastropoda) from the tropical southwest Pacific. In: Héros V, Cowie RH, Bouchet P (Eds), Tropical Deep-Sea Benthos 25. Mémoires du Muséum national d’Histoire naturelle. 587–792.

[B220] Vendetti JE Trowbridge CD Krug PJ (2012) Poecilogony and population genetic structure in *Elysia pusilla* (Heterobranchia: Sacoglossa), and reproductive data for five sacoglossans that express dimorphisms in larval development. Integrative and Comparative Biology 52: 138–150. 10.1093/icb/ics077 22659202

[B221] Warén A (1981) Revision of the genera *Apicalia* A. Adams and *Stilapex* lredale and description of two new genera (Mollusca, Prosobranchia, Eulimidae). Zoologica Scripta 10: 133–154. 10.1111/j.1463-6409.1981.tb00491.x

[B222] Warén A (1984) A generic revision of the family Eulimidae (Gastropoda, Prosobranchia). Journal of Molluscan Studies suppl. 13: 1–96. 10.1093/mollus/49.Supplement_13.1

[B223] Warén A (1991a) New and little known Mollusca from Iceland and Scandinavia. Sarsia 76: 53–124. 10.1080/00364827.1991.10413466

[B224] Warén A (1991b) Revision of *Hypermastus* Pilsbry, 1899 and *Turveria* Berry, 1956 (Gastropoda: Prosobranchia: Eulimidae), two genera parasitic on sand dollars. Records of the Australian Museum 43: 85–112. 10.3853/j.0067-1975.43.1991.42

[B225] Zenetos A Ovalis P (2014) Alien Mollusca in the Levantine Sea: an update. Occurrence of *Ervilia scaliola* Issel, 1869 along the Levantine coast of Turkey. Cahiers de Biologie Marine 55: 507–512. 10.21411/CBM.A.67AC08A4

[B226] Zenetos A Galanidi M (2020) Mediterranean non indigenous species at the start of the 2020s: recent changes. Marine Biodiversity Records 13: 10. 10.1186/s41200-020-00191-4

[B227] Zenetos A Corsini-Foka M Crocetta F Gerovasileiou V Simboura N Tsiamis K Pancucci-Papadopoulou M-A (2018) Deep cleaning of alien and cryptogenic species records in the Greek Seas (2018 update). Management of Biological Invasions 9: 209–226. 10.3391/mbi.2018.9.3.04

[B228] Zenetos A Çinar ME Crocetta F Golani D Rosso A Servello G Shenkar N Turon X Verlaque M (2017) Uncertainties and validation of alien species catalogues: The Mediterranean as an example. Estuarine, Coastal and Shelf Science 191: 171–187. 10.1016/j.ecss.2017.03.031

[B229] Zenetos A Gofas S Verlaque M Cinar ME Raso JEG Bianchi CN Morri C Azzurro E Bilecenoglu M Froglia C Siokou I Violanti D Sfriso A Martin GS Giangrande A Katagan T Ballesteros E Ramos-Espla AA Mastrototaro F Ocana O Zingone A Gambi MC Streftaris N (2010) Alien species in the Mediterranean Sea by 2010. A contribution to the application of European Union’s Marine Strategy Framework Directive (MSFD). Part I. Spatial distribution. Mediterranean Marine Science 11: 381. 10.12681/mms.87

[B230] Zorina IP (1978) New species of clams (Bivalvia) from the Tonkin Bay (South China sea). Trudy Zoologicheskogo Instituta AN SSSR 61: 193–203.

[B231] Zuschin M Oliver PG (2003) Bivalves and bivalve habitats in the northern Red Sea. The Northern Bay of Safaga (Red Sea, Egypt): An actuopalaeontological approach. VI. Bivalvia. Naturhistorisches Museum Wien, 304 pp.

